# Towards precision medicine for stress disorders: diagnostic biomarkers and targeted drugs

**DOI:** 10.1038/s41380-019-0370-z

**Published:** 2019-03-12

**Authors:** H. Le-Niculescu, K. Roseberry, D. F. Levey, J. Rogers, K. Kosary, S. Prabha, T. Jones, S. Judd, M. A McCormick, A. R. Wessel, A. Williams, P. L. Phalen, F. Mamdani, A. Sequeira, S. M. Kurian, A. B. Niculescu

**Affiliations:** 1 Department of Psychiatry, Indiana University School of Medicine, Indianapolis, IN USA; 2 Indianapolis VA Medical Center, Indianapolis, IN USA; 3 Department of Psychiatry and Human Behavior, UC Irvine, Irvine, CA USA; 4 Department of Molecular and Experimental Medicine, The Scripps Research Institute, La Jolla, CA USA; 5 Stark Neuroscience Research Institute, Indiana University School of Medicine, Indianapolis, IN USA

**Keywords:** Predictive markers, Genetics

## Abstract

The biological fingerprint of environmental adversity may be key to understanding health and disease, as it encompasses the damage induced as well as the compensatory reactions of the organism. Metabolic and hormonal changes may be an informative but incomplete window into the underlying biology. We endeavored to identify objective blood gene expression biomarkers for psychological stress, a subjective sensation with biological roots. To quantify the stress perception at a particular moment in time, we used a simple visual analog scale for life stress in psychiatric patients, a high-risk group. Then, using a stepwise discovery, prioritization, validation, and testing in independent cohort design, we were successful in identifying gene expression biomarkers that were predictive of high-stress states and of future psychiatric hospitalizations related to stress, more so when personalized by gender and diagnosis. One of the top biomarkers that survived discovery, prioritization, validation, and testing was FKBP5, a well-known gene involved in stress response, which serves as a de facto reassuring positive control. We also compared our biomarker findings with telomere length (TL), another well-established biological marker of psychological stress and show that newly identified predictive biomarkers such as NUB1, APOL3, MAD1L1, or NKTR are comparable or better state or trait predictors of stress than TL or FKBP5. Over half of the top predictive biomarkers for stress also had prior evidence of involvement in suicide, and the majority of them had evidence in other psychiatric disorders, providing a molecular underpinning for the effects of stress in those disorders. Some of the biomarkers are targets of existing drugs, of potential utility in patient stratification, and pharmacogenomics approaches. Based on our studies and analyses, the biomarkers with the best overall convergent functional evidence (CFE) for involvement in stress were FKBP5, DDX6, B2M, LAIR1, RTN4, and NUB1. Moreover, the biomarker gene expression signatures yielded leads for possible new drug candidates and natural compounds upon bioinformatics drug repurposing analyses, such as calcium folinate and betulin. Our work may lead to improved diagnosis and treatment for stress disorders such as PTSD, that result in decreased quality of life and adverse outcomes, including addictions, violence, and suicide.

## Introduction

“The conflict between the will to deny horrible events and the will to proclaim them aloud is the central dialectic of psychological trauma.”

― Judith Lewis Herman

Stress disorders, such as post-traumatic stress disorder (PTSD), are prevalent, disabling, and underdiagnosed in both the military and civilian realm [1–3]. Stress disorders consist of mental and physical over-reaction to environmental cues that are perceived as potentially harmful, engendered by past exposure to traumatic events. The persistence, intensity, dis-congruence from the environment, or congruence with excessive response are all hallmarks of clinical illness. Stress disorders affect one’s ability to do things and quality of life. Owing to stigma and lack of objective tests, they are often underdiagnosed, sub-optimally treated, and can lead to self-medication with alcohol and drugs. They may culminate in some cases with violence and suicide. Psychiatric patients may have an increased vulnerability to stress, regardless of their primary diagnosis, as well as increased reasons for stress disorders, due to their often adverse life trajectory. As such, they may be a particularly suitable population in which to try to identify blood biomarkers for stress that are generalizable and trans-diagnostic.

First, we used a powerful longitudinal within-subject design in individuals with psychiatric disorders to discover blood gene expression changes between self-reported low- and high-stress states. Second, we prioritized the list of candidate biomarkers with a Convergent Functional Genomics (CFG) approach, comprehensively integrating previous published human and animal model evidence in the field and directly citing it. Third, we validated our top biomarkers from discovery and prioritization in an independent cohort of psychiatric subjects with high scores on a clinical stress rating scale. Fourth, we tested whether the candidate biomarkers from the first three steps are able to predict high-stress state, and future psychiatric hospitalizations with stress, in another independent cohort of psychiatric subjects. We tested the biomarkers in all subjects in the independent test cohort, as well as in a more personalized fashion by gender and psychiatric diagnosis, showing increased accuracy with the personalized approach. Fifth, we assessed whether our biomarkers have evidence for involvement in other psychiatric and related disorders, as well as analyzed the biological pathways and networks they are involved in. Sixth, we identified which of our biomarkers are targets of existing drugs and thus can be used for pharmacogenomic population stratification and measuring of response to treatment. We also used the gene expression signatures of the top predictive biomarkers to interrogate the Connectivity Map database from Broad/MIT to identify drugs and natural compounds that can be repurposed for treating stress. Given the negative impact of untreated stress on quality (and quantity) of life, the current lack of objective measures to determine appropriateness of treatment, and the mixed results with existing medications, the importance of approaches such as ours cannot be overstated.

## Materials and methods

### Cohorts

**Fig. 1:**
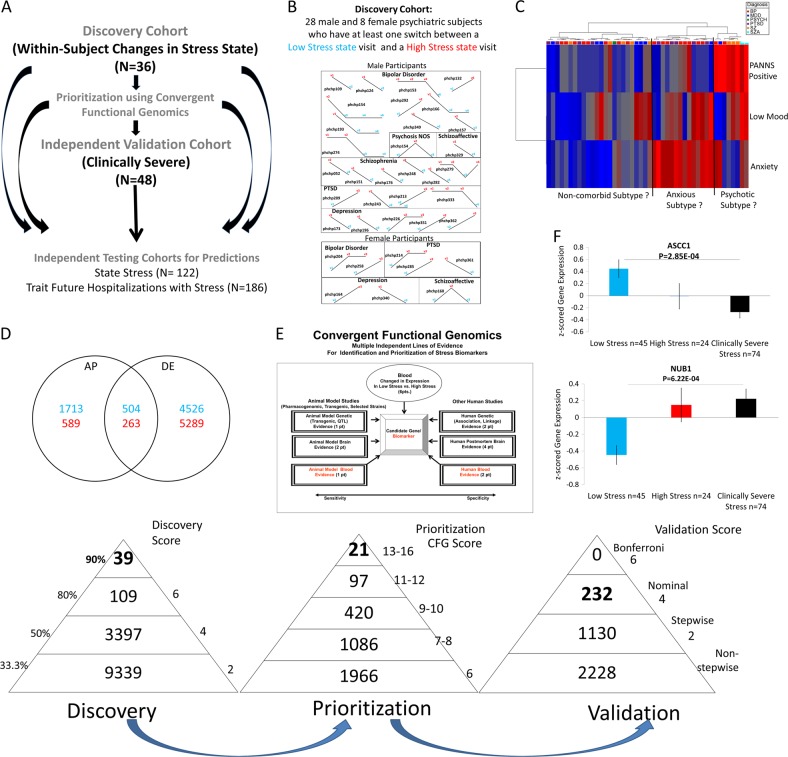
Steps 1–3: Discovery, prioritization and validation. **a** Cohorts used in study, depicting flow of discovery, prioritization, and validation of biomarkers from each step. **b** Discovery cohort longitudinal within-subject analysis. Phchp### is study ID for each subject. V# denotes visit number. **c** Discovery of possible subtypes of stress based on High Stress visits in the discovery cohort. Subjects were clustered using measures of mood and anxiety (from Simplified Affective State Scale (SASS)) [7], as well as psychosis (PANNS Positive). **d** Differential gene expression in the Discovery cohort—number of genes identified with differential expression (DE) and absent–present (AP) methods with an internal score of ≥2. Red—increased in expression in High Stress, blue—decreased in expression in High Stress. At the discovery step, probesets are identified based on their score for tracking stress with a maximum of internal points of 6 (33% (2 pt), 50% (4 pt) and 80% (6 pt)). **e** Prioritization with Convergent Functional Genomics (CFG) for prior evidence of involvement in stress. In the prioritization step, probesets are converted to their associated genes using Affymetrix annotation and GeneCards. Genes are prioritized and scored using CFG for stress evidence with a maximum of 12 external points. Genes scoring at least 6 points out of a maximum possible of 18 total internal and external scores points are carried to the validation step. **f** Validation in an independent cohort of psychiatric patients with clinically severe trait stress and high-state stress. In the validation step, biomarkers are assessed for stepwise change from the discovery groups of subjects with Low Stress, to High Stress, to Clinically Severe Stress, using analysis of variance. *N* = number of testing visits. Two hundred and thirty-two biomarkers were nominally significant, NUB1 and ASCC1 were the most significant increased and decreased biomarkers, respectively, and 1130 biomarkers were stepwise changed

We used three independent cohorts: discovery (major psychiatric disorders with changes in state stress), validation (major psychiatric disorders with clinically severe trait and state stress), and testing (an independent major psychiatric disorders cohort for predicting state stress and for predicting trait future hospitalization visits with stress as the primary reason) (Fig. 1a).

Similar to our previous studies [4–6], the psychiatric subjects are part of a larger longitudinal cohort of adults that we are continuously collecting. Subjects were recruited from the patient population at the Indianapolis VA Medical Center. All subjects understood and signed informed consent forms detailing the research goals, procedure, caveats, and safeguards, per Institutional Review Board-approved protocol. Subjects completed diagnostic assessments by an extensive structured clinical interview—Diagnostic Interview for Genetic Studies—and up to six testing visits, 3–6 months apart or whenever a new psychiatric hospitalization occurred. At each testing visit, they received a series of rating scales, including a self-report visual analog scale (1–100) for quantitatively assessing *state* stress at that particular moment in time (Simplified Stress Scale), which has 4 items (Life Stress, Financial Stress, Health Stress, and Social Stress). We also administered the PTSD Checklist—Civilian Version (PCL-C) scale, which measures clinical severity of *trait* stress symptoms over the month preceding testing. We collected whole blood (10 ml) in two RNA-stabilizing PAXgene tubes, labeled with an anonymized ID number, and stored at −80 °C in a locked freezer until the time of future processing. Whole-blood RNA was extracted for microarray gene expression studies from the PAXgene tubes, as detailed below.

For this study, our within-subject discovery cohort, from which the biomarker data were derived, consisted of 36 subjects (28 males, 8 females) with multiple testing visits, who each had at least one diametric change in stress state from low-stress state (visual analog scale (VAS) Life Stress score of ≤33/100) to a high-stress state (Life Stress score of ≥67/100), or vice versa, from one testing visit to another. We also required that at least one of the other items (Health Stress, Financial Stress, or Social Stress) must have concording low or high score with the Life Stress ((Fig. 1 and Figure S1). There were 5 subjects with 4 visits each, 9 subjects with 3 visits each, and 22 subjects with 2 visits each resulting in a total of 91 blood samples for subsequent gene expression microarray studies (Fig. 1, Table 1 and S1).

**Table 1:** Aggregate demographics

Cohorts	Number of subjects	Gender	Diagnosis	Ethnicity	Age at the time of visit, mean (SD)	*T* test for age
Discovery						
Discovery cohort (within-subject changes in life stress VAS) Low life stress VAS ≤ 33 to high life stress VAS ≥ 67 Concordance with 1 other item (health stress, financial stress, social stress)	36 (with 91 visits)	Male = 28 Female = 8	BP 14 (38) MDD 7 (15) PSYCH 1 (3) PTSD 6 (16) SZ 6 (14) SZA 2 (5)	EA = 25 AA = 10 Hispanic = 1	All = 49.8022 (10.3754) Low stress = 50.31 High stress = 49.30	
Validation						
Independent validation cohort (clinically severe stress PCL-C ≥ 50; life stress VAS ≥ 67)	48 (75 visits)	Male = 35 Female = 13	MDD = 13 BP = 8 SZ = 2 SZA = 7 PTSD = 13 MOOD = 4	EA = 37 AA = 10	48.96 (8.4)	Discovery vs. validation 0.56523437
Testing						
Independent testing cohort for predicting state (high stress state life stress VAS ≥ 67 at the time of assessment)	122 (258 visits)	Male = 95 Female = 27	BP = 53 MDD = 24 SZA = 15 SZ = 17 PTSD = 9 MOOD = 1 PSYCH = 3	EA = 89 AA = 31 Mixed = 1 Hispanic = 1	All = 45.5 (9.93) Others = 46.2 High Stress = 44.03	High stress (*n* = 38) vs. others (*N* = 220) 0.50720396
Independent testing cohort for predicting trait (future hospitalizations with stress in the first year following assessment)	162 (398 visits)	Male = 144 Female = 18	BP = 50 MDD = 27 SZA = 32 SZ = 39 PTSD = 8 MOOD = 3 PSYCH = 8	EA = 101 AA = 58 Mixed = 1 Hispanic = 2	All = 50.4 (8.19) Others = 48.6 Hosp with stress = 47.9	Hosp with stress (*n* = 32) vs. others (*n* = 366) 0.7001408
Independent testing cohort for predicting trait (future hospitalizations with stress in all years following assessment)	186 (474 visits)	Male = 166 Female = 20	BP = 56 MDD = 30 SZA = 47 SZ = 39 PTSD = 8 MOOD = 3 PSYCH = 3	EA = 119 AA = 64 Mixed = 1 Hispanic = 2	All = 50.45 (8.86) Others = 50.55 Hosp with Stress = 50.12	Hosp with stress (*n* = 113) vs. others (*n* = 361) 0.65942853

*BP* bipolar, *MDD* depression, *MOOD* mood nos., *SZ* schizophrenia, *SZA* schizoaffective, *PSYCH* psychosis nos., *PCL-C* PTSD Checklist—Civilian Version, *PTSD* post-traumatic stress disorder, *VAS* visual analog scale, *EA* European Americans, *AA* African Americans

Our independent validation cohort, in which the top biomarker findings were validated for being even more strongly changed in expression compared to our discovery cohort, consisted of 35 male and 13 female subjects with both high *trait* stress (PTSD PCL-C scale scores ≥50, indicating clinically severe stress) and high *state* stress (VAS Life Stress score of ≥67/100) (Table 1).

Our independent test cohort for predicting state high stress consisted of 95 male and 27 female subjects with psychiatric disorders, demographically matched with the discovery cohort, with one or multiple testing visits in our laboratory, with either Low Stress, Intermediate Stress, or High Stress (Fig. 1 and Table 1).

Our test cohort for predicting trait future hospitalization visits with stress symptoms, in the first year of follow-up, and all future hospitalization visits with stress symptoms (Fig. 1) consisted of 166 males and 20 female subjects for which we had longitudinal follow-up with electronic medical records. The subjects’ subsequent number of hospitalization with stress symptoms in the year following testing was tabulated from electronic medical records by a clinical researcher, who examined admission and discharge summaries.

#### Medications

The subjects in the discovery cohort were all diagnosed with various psychiatric disorders (Table 1) and had various medical co-morbidities. Their medications were listed in their electronic medical records and documented by us at the time of each testing visit. Medications can have a strong influence on gene expression. However, our discovery of differentially expressed genes was based on within-subject analyses, which factor out not only genetic background effects but also minimizes medication effects, as the subjects rarely had major medication changes between visits. Moreover, there was no consistent pattern of any particular type of medication, as our subjects were on a wide variety of different medications, psychiatric and non-psychiatric. Furthermore, the independent validation and testing cohort gene expression data was *Z*-scored by gender and diagnosis before being combined, to normalize for any such effects. Some subjects may be non-compliant with their treatment and may thus have changes in medications or drug of abuse not reflected in their medical records. That being said, our goal is to find biomarkers that track stress, regardless if the reason for it is endogenous biology or driven by substance abuse or medication non-compliance. In fact, one would expect some of these biomarkers to be direct or indirect targets of medications, as we show in this paper. Overall, the discovery, validation, and replication by testing in independent cohorts of the biomarkers, with our design, occurs despite the subjects having different genders, diagnoses, being on various different medications, and other lifestyle variables.

### Blood gene expression experiments

#### RNA extraction

Whole blood (2.5 ml) was collected into each PaxGene tube by routine venipuncture. PaxGene tubes contain proprietary reagents for the stabilization of RNA. RNA was extracted and processed as previously described, including standard globin clear and RNA quality assessment steps [4–6].

#### Microarrays

Microarray work was carried out using previously described methodology [4–7]. The dataset is available at GEO (GSE125216). 

### Telomere length (TL)

Blood was collected in EDTA blood tubes and kept at −80 °C until the time of extraction. DNA was extracted using the DNeasy Blood & Tissue Kit (Qiagen) and DNA concentration was assessed using Qubit (ThermoFisher Scientific) as per the manufacturer’s protocols. TL was determined using a relative quantitative real-time PCR method [8]. Two assays were carried out, one for the Human albumin gene (ALB), which is a single copy gene, and the other assay with primers specific to the repetitive telomeric (TEL) sequence. The primers used to amplify the single copy gene are: ALBF (CTG TCA TCT CTT GTG GGC TGT) and ALBR (GGC ATG ACA GGT TTT GCA ATA) and those for the telomeric sequence are: TEL1b (CGG TTT GTT TGG GTT TGG GTT TGG GTT TGG GTT TGG GTT) and TEL2b (GGC TTG CCT TAC CCT TAC CCT TAC CCT TAC CCT TAC CCT). A ratio of the relative quantities (TEL/ALB) was used as a quantitative measure of TL. Each sample was run in triplicate and an average of the cycle thresholds was used to calculate telomere/single copy gene (T/S) ratios.

### Biomarkers

#### Step 1: Discovery

We used the subject’s score from a VAS Life Stress, assessed at the time of blood collection (Figure S1). We analyzed gene expression differences between visits with Low Stress (defined as a score of 0–33) and visits with High Stress (defined as a score of 67–100) (Fig. 1 and S1).

We analyzed the data in two ways: an absent–present (AP) approach, and a differential expression (DE) approach, as in previous work by us on suicide biomarkers [4–6]. The AP approach may capture turning on and off of genes, and the DE approach may capture gradual changes in expression. We used a powerful within-subject design, then an across-subjects summation score for probesets. Analyses were performed as previously described [5–7]. In brief, we imported all Affymetrix microarray data as CEL. files into Partek Genomic Suites 6.6 software package (Partek Incorporated, St Louis, MI, USA). Using only the perfect match values, we ran a robust multi-array analysis (RMA) by gender and diagnosis, background corrected with quantile normalization and a median polish probeset summarization of all chips, to obtain the normalized expression levels of all probesets for each chip. Then, to establish a list of differentially expressed probesets we conducted a within-subject analysis, using a fold change in expression of at least 1.2 between high- and low-stress visits within each subject. Probesets that have a 1.2-fold change are then assigned either a 1 (increased in high stress) or a −1 (decreased in high stress) in each comparison. These values were then summed for each probeset across all the comparisons and subjects, yielding a range of raw scores. The probesets above the 33.3% of scores received an internal score of 2 points, those >50% 4 points, and those >80% 6 points [5–7]. We have developed in our laboratories R scripts to automate and conduct all these large dataset analyses in bulk, checked against human manual scoring [7].

Gene Symbol for the probesets were identified using NetAffyx (Affymetrix) for Affymetrix HG-U133 Plus 2.0 GeneChips, followed by GeneCards to confirm the primary gene symbol. In addition, for those probesets that were not assigned a gene symbol by NetAffyx, we used GeneAnnot (https://genecards.weizmann.ac.il/geneannot/index.shtml) or UCSC (https://genome.ucsc.edu) to obtain gene symbol for these uncharacterized probesets, followed by GeneCard. Genes were then scored using our manually curated CFG databases as described below (Fig. 1e).

#### Step 2: Prioritization using CFG databases

We have established in our laboratory (Laboratory of Neurophenomics, www.neurophenomics.info) manually curated databases of the human gene expression/protein expression studies (postmortem brain, peripheral tissue/fluids: cerebrospinal fluid, blood, and cell cultures), human genetic studies (association, copy number variations, and linkage), and animal model gene expression and genetic studies, published to date on psychiatric disorders. Only findings deemed significant in the primary publication, by the study authors, using their particular experimental design and thresholds, are included in our databases. Our databases include only primary literature data and do not include review papers or other secondary data integration analyses to avoid redundancy and circularity. These large and constantly updated databases have been used in our CFG cross-validation and prioritization platform (Fig. 1e). For this study, data from 354 papers on stress were present in the databases at the time of the CFG analyses (February 2018) (human genetic studies—93, human brain studies—10, human peripheral tissue/fluids—96, non-human genetic studies—17, non-human brain studies—123, non-human peripheral tissue/fluids—17). Analyses were performed as previously described [5, 6].

#### Step 3: Validation analyses

We examined which of the top candidate genes (total CFG score of ≥6) were stepwise changed in expression from the Low Stress Discovery group to the High Stress Discovery group to the Clinically Severe Stress Validation group. A CFG score of ≥6 reflects an empirical cutoff of 33.3% of the maximum possible total CFG score of 18, which permits the inclusion of potentially novel genes with maximal internal score of 6 but no external evidence score. Subjects with Low Stress as well as subjects with High Stress from the discovery cohort who did not have severe clinical stress (PCL-C < 50) were used, along with the independent validation cohort (*n* = 48 subjects).

The AP- and DE-derived lists of genes were combined, and the gene expression data corresponding to them was used for the validation analysis. The cohorts (Validation Clinically Severe Stress, alongside the Low Stress and High Stress groups in the Discovery cohort) were assembled out of Affymetrix.cel data that was RMA normalized by gender and diagnosis. We transferred the log-transformed expression data to an Excel sheet, and non-log transformed the data by taking 2 to the power of the transformed expression value. We then *Z*-scored the values by gender and diagnosis. We then imported the Excel sheets with the *Z*-scored by gender and diagnosis expression data into Partek, and statistical analyses were performed using a one-way analysis of variance (ANOVA) for the stepwise changed probesets and also attempted a stringent Bonferroni corrections for all the probesets tested (Fig. 1f). We also wrote an R script that automatically analyzes the data directly from the Excel sheet and used that to confirm our calculations.

### Biomarkers carried forward

We carried forward into testing the top biomarkers from each of the Steps 1–3. The list of candidate biomarkers includes the top biomarkers from discovery step (≥90% of raw scores, *n* = 39), the top biomarkers from the prioritization step (CFG score ≥ 13, *n* = 21), and the nominally significant biomarkers after the validation step (*n* = 232), for a total of *n* = 285 probesets (*n* = 269 genes). We then predict with the biomarkers from the list in independent cohort state (High Life Stress VAS ≥ 67/100), and trait (Future Hospitalizations with Stress), in the first year of follow-up, and in all future years of follow-up.

### Diagnostics

In Step 4, testing, the test cohort for predicting High Stress (state) and the test cohort for predicting Future Hospitalizations with Stress (trait), were assembled out of data that was RMA normalized by gender and diagnosis. The cohort was completely independent from the discovery and validation cohorts; there was no subject overlap with them. Phenomic (clinical) and gene expression markers used for predictions were *Z*-scored by gender and diagnosis, to be able to combine different markers into panels and to avoid potential artifacts due to different ranges of expression in different gender and diagnoses. Markers were combined by simple summation of the increased risk markers minus the decreased risk markers. Predictions were performed using R-studio. For cross-sectional analyses, we used marker expression levels, *Z*-scored by gender and diagnosis. For longitudinal analyses, we combined four measures: marker expression levels, slope (defined as ratio of levels at current testing visit vs. previous visit, divided by time between visits), maximum levels (at any of the current or past visits), and maximum slope (between any adjacent current or past visits). For decreased markers, we used the minimum rather than the maximum for level calculations. All four measures were *Z*-scored, then combined in an additive fashion into a single measure. The longitudinal analysis was carried out in a sub-cohort of the testing cohort consisting of subjects that had at least two test visits.

#### Predicting state: high stress

**Fig. 2:**
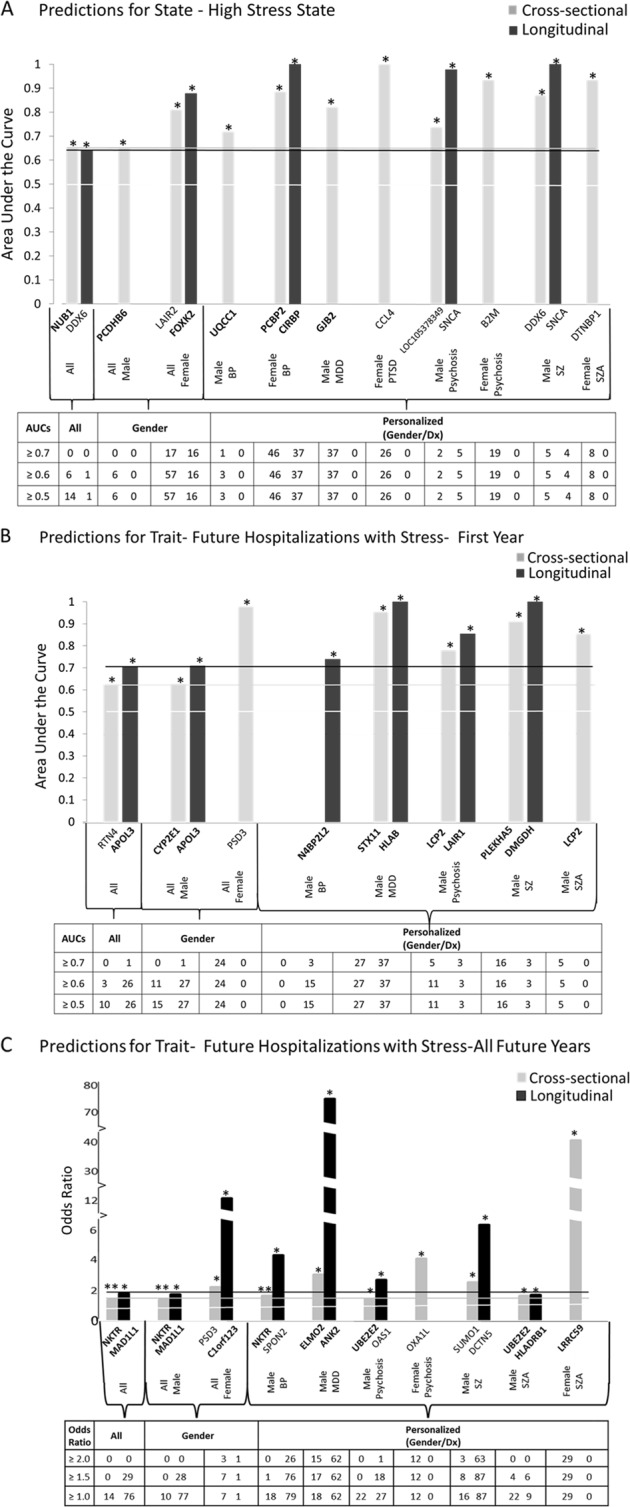
Best predictive biomarkers. From among top candidate biomarkers (*n* = 285) from Steps 1–3 (Discovery—39, Prioritization—21, Validation—232 bolded). Bar graph shows best predictive biomarkers in each group. *Nominally significant for predictions *p* < 0.05. **Bonferroni significant for the 285 biomarkers tested. Table underneath the figures displays the actual number of biomarkers for each group whose area under the receiver-operating characteristic curve *p* values (**a**, **b**) and Cox odds ratio *p* values (**c**) are at least nominally significant. Some gender and diagnosis groups are missing from the graph as they did not have any significant biomarkers. Cross-sectional is based on levels at one visit. Longitudinal is based on levels at multiple visits (integrates levels at most recent visit, maximum levels, slope into most recent visit, and maximum slope). Dividing lines represent the cutoffs for a test performing at chance levels (white) and at the same level as the best biomarkers for all subjects in cross-sectional (gray) and longitudinal (black) based predictions. All biomarkers perform better than chance. Biomarkers performed better when personalized by gender and diagnosis

Receiver-operating characteristic (ROC) analyses between marker levels and stress state were performed by assigning subjects visits with a Life Stress VAS score of ≥67 into the High Stress category. We used the pROC package of R [9] (Table 2, Fig. 2). Additionally, a one-tailed *t* test was performed between High Stress group vs. the rest, and Pearson *R* (one-tail) was calculated between Life Stress VAS scores and marker levels (Supplementary Information- Complete Datasets and Analyses).

**Table 2:** Convergent Functional Evidence (CFE) for best predictive biomarkers for stress (from Fig. 2)

Gene symbol/gene name	Probesets	Step 1	Step 2	Step 3	Step 4	Step 4	Step 4	Step 5	Step 6	CFE Polyevidence Score
		Discovery in blood	External CFG evidence for involvement in stress	Validation in blood	Best significant prediction of high stress state	Best significant prediction of first-year Hosp with stress	Best Significant predictions of all future Hosp with stress	Other psychiatric and related disorders evidence—change in same direction as stress	Pharmacogenomics drugs that modulate the biomarker in opposite direction to stress	
		(Direction of change) method/score/%	Score	ANOVA *p* value/score	ROC AUC/*p* value	ROC AUC/*p* value	OR/OR *p* value			
		6 pts	12 pts	6 pts	8 pts All; 6 pts Gender; 4 pts Gender/Dx	8 pts All; 6 pts Gender; 4 pts Gender/Dx	8 pts All; 6 pts Gender; 4 pts Gender/Dx	3 pts	3 pts	
TL Telomere Length Reference marker from literature	NA	NA	7	**NS**	**Gender/Dx** M-MDD C: (2/14) 1/1.42E-02	**All** C: (14/108) 0.72/4.82E-03 **Gender** Male C: (14/86) 0.73/3.21E-03 **Gender/Dx** M-MDD C: (4/17) 0.90/8.71E-03 M-BP C: (9/55 0.68/4.19E-02		Aging Alcohol Depression Mania Psychosis	Omega-3 fatty acids Lithium Olanzapine Mianserin	25
FKBP5 FK506 Binding Protein 5	224856_at	(D) DE/4 53.8%	12	**1.22E-02/4 Nominal**	**Gender** Female C: (13/60) 0.65/4.85E-02 **Gender/Dx** F-BP C: (6/22) 0.82/1.11E-02	**Gender/Dx** M-MDD C: (5/49) 0.75/3.72E-02 L: (2/27) 0.9/3.20E-02	**Gender/Dx** M-SZ L: (8/56) 4.6/3.94E-02	Alcohol Anxiety BP Depression MDD Pain Psychosis Unipolar Depression Suicide	Mood stabilizers	40
DDX6 DEAD-Box Helicase 6	1562836_at	(I) DE/6 83.8% (I) AP/6 90.2%	9	Not stepwise	**All** L: (13/134) 0.64/4.79E-02 **Gender** Female C: (13/60) 0.7/1.60E-02 L: (5/33) 0.79/2.23E-02 **Gender/Dx** F-BP C: (6/22) 0.82/1.11E-02 L: (2/12) 0.9/4.28E-02 M-PSYCHOSIS C: (5/47) 0.73/4.88E-02 L: (2/24) 0.95/1.84E-02 M-SZ C: (4/29) 0.87/9.64E-03 L: (2/15) 1/1.36E-02	**All** L: (14/234) 0.63/4.59E-02 **Gender** Male L: (14/206) 0.64/4.00E-02 **Gender/Dx** M-BP L: (10/77) 0.71/1.63E-02	**All** L: (62/286) 1.3/4.41E-02 **Gender** Male L: (59/253) 1.4/1.66E-02 **Gender/Dx** M-BP L: (24/91) 1.8/2.75E-05	Alcohol BP Other substances/addictions MDD Yohimbine Suicide		36
B2M Beta-2-Microglobulin	232311_at	(I) DE/6 91.2%	5	Not stepwise	**Gender/Dx** F-PSYCHOSIS C: (4/19) 0.93/4.66E-03 F-SZA C: (3/13) 0.9/2.13E-02	**Gender** Female C: (2/46) 0.94/1.78E-02	**All** C: (113/474) 1.2/3.09E-02 L: (62/286) 1.5/9.79E-03 **Gender** Female C: (7/53) 1.8/4.87E-02 Male L: (59/253) 1.5/6.83E-03 **Gender/Dx** M-BP C: (41/140) 1.4/2.02E-03 L: (24/91) 2.3/5.64E-04	Alcohol Aging Autism Eating disorder MDD Depression Pain Suicide	Omega-3 fatty acids, 4’-iodo-4’-deoxydoxorubicin	35
LAIR1 Leukocyte Associated Immunoglobulin Like Receptor 1	210644_s_at	(D) DE/6 86.2%	4	**1.12E-02/4 Nominal**	**Gender** Female L: (5/33) 0.75/3.94E-02	**Gender/Dx** M-PSYCHOSIS L: (2/95) 0.85/4.35E-02	**All** L: (62/286) 1.7/1.68E-03 **Gender** Male L: (59/253) 1.7/2.09E-03 **Gender/Dx** M-BP L: (24/91) 2/1.76E-02 M-PSYCHOSIS L: (29/121) 1.7/1.22E-02	Suicide		35
RTN4 Reticulon 4	1556049_at	(I) DE/4 54.4%	9	Not stepwise		**All** C: (32/398) 0.63/9.49E-03 **Gender** Female C: (2/46) 0.85/4.75-02 Male C: (30/352) 0.61/2.32-02	**All** C: (113/474) 1.18/2.26-02 **Gender** Male C: (106/421) 1.16/4.30-02 **Gender/Dx** M-BP C: (41/140) 1.29/4.95-02 M-MDD C: (9/57) 2.21/1.33-02 F-SZA C: (3/12) 5.4/4.76-02	Alcohol BP Suicide Pain	Omega-3 fatty acids Valproate	35
NUB1 Negative Regulator Of Ubiquitin Like Proteins 1	1560108_at (1560109_s_at)	(I) DE/4 61.8%	8	**2.34E-02/4 Nominal (6.22E-04/4 Top Nominal)**	**All** C: (38/258) 0.65/1.42E-03 **Gender** Female C: (13/60) 0.74/3.96E-03 Male C: (25/198) 0.6/4.70E-02 **Gender/Dx** F-BP C: (6/22) 0.78/2.33E-02		**Gender/Dx** M-PSYCHOSIS C: (52/201) 1.2/2.72E-02 L: (29/121) 1.5/1.37E-02 M-SZ L: (8/56) 1.6/2.20E-02	Autism Suicide	Antipsychotics	34
CIRBP Cold Inducible RNA Binding Protein	200811_at	(D) DE/4 69.2%	4	**3.66E-02/4 Nominal**	**Gender** Female C: (13/60) 0.65/4.67E-02 **Gender/Dx** F-BP C: (6/22) 0.76/3.27E-02 L: (2/12) 1/1.58E-02	**All** L: (14/234) 0.68/1.19E-02 **Gender** Male L: (14/206) 0.68/1.17E-02 **Gender/Dx** M-BP L: (10/77) 0.67/4.63E-02 M-SZ C: (3/74) 0.79/4.59E-02	**Gender/Dx** M-BP L: (24/91) 1.9/1.99E-02 M-MDD L: (4/32) 13/3.39E-02 M-SZ L: (8/56) 4.1/1.23E-02	Autism SZ		33
CYP2E1 Cytochrome P450 Family 2 Subfamily E Member 1	209976_s_at	(I) DE/2 44.1%	6	**1.57E-02/4 Nominal**	**Gender/Dx** F-BP C: (6/22) 0.78/2.33E-02 M-MDD C: (6/35) 0.77/1.98E-02	**All** C: (32/398) 0.6/3.41E-02 **Gender** Male C: (30/352) 0.63/1.09E-02 **Gender/Dx** M-PSYCHOSIS C: (8/161) 0.74/1.04E-02 M-SZA C: (5/87) 0.82/7.64E-03	**Gender** Male L: (59/253) 1.3/4.96E-02 **Gender/Dx** M-PSYCHOSIS L: (29/121) 1.6/9.44E-03 M-SZ C: (13/93) 1.4/3.85E-02 L: (8/56) 2.1/2.50E-03	Alcohol SZ Suicide		33
MAD1L1 MAD1 Mitotic Arrest Deficient Like 1	204857_at	(D) DE/4 72.3%	2	**1.47E-02/4 Nominal**	**Gender/Dx** F-PSYCHOSIS C: (4/19) 0.78/4.45E-02	**All** L: (14/236) 0.64/4.24E-02 **Gender** Male L: (14/208) 0.64/4.07E-02	**All** L: (62/288) 1.8/1.32E-03 **Gender** Male L: (59/255) 1.7/2.66E-03 **Gender/Dx** M-BP L: (24/91) 2.1/9.71E-03 M-MDD L: (4/32) 31.4/5.50E-03	Autism BP Cocaine SZ		33
OAS1 2’-5’-Oligoadenylate Synthetase 1	202869_at	(D) DE/4 56.9%	9	1.15E-01/2 Stepwise	**All** C: (38/258) 0.6/2.77E-02 **Gender** Female C: (13/60) 0.66/3.71E-02 **Gender/Dx** F-PSYCHOSIS C: (4/19) 0.8/3.59E-02		**Gender/Dx** M-PSYCHOSIS L: (29/121) 2.7/1.52E-02 M-SZ L: (8/56) 3.5/4.35E-02	Alcohol Alzheimer’s Panic disorder MDD	Mood stabilizers	33
OXA1L OXA1L, Mitochondrial Inner Membrane Protein	208717_at	(D) DE/4 56.9%	6	**6.40E-03/4 Nominal**	**Gender/Dx** F-BP C: (6/22) 0.75/3.84E-02	**Gender/Dx** M-MDD L: (2/27) 0.86/4.78E-02	**All** L: (62/288) 1.5/1.14E-02 **Gender** Male L: (59/255) 1.5/2.04E-02 **Gender/Dx** F-PSYCHOSIS C: (6/17) 4.2/3.02E-02 M-MDD L: (4/32) 3.5/4.37E-02 M-SZ L: (8/56) 4.7/2.19E-02	Autism BP Suicide SZ		33
CCL4 C-C Motif Chemokine Ligand 4	204103_at	(D) DE/6 96.9%	2	Not stepwise	**Gender/Dx** F-PTSD C: (3/7) 1/1.69E-02 M-MDD C: (6/35) 0.75/2.99E-02	**All** L: (14/234) 0.66/2.01E-02 **Gender** Male L: (14/206) 0.66/2.07E-02 **Gender/Dx** M-MDD L: (2/27) 0.94/2.08E-02	**All** L: (62/286) 1.4/3.22E-02 **Gender** Male L: (59/253) 1.6/1.01E-02 **Gender/Dx** M-BP L: (24/91) 2.2/5.34E-03 M-MDD L: (4/32) 54.5/2.12E-02	Alcohol Depression MDD SZ		31
DTNBP1 Dystrobrevin Binding Protein 1	223446_s_at	(D) DE/6 93.8%	4	Not stepwise	**Gender** Female C: (13/60) 0.7/1.33E-02 **Gender/Dx** F-PSYCHOSIS C: (4/19) 0.9/8.20E-03 F-SZA C: (3/13) 0.93/1.40E-02	**Gender/Dx** M-MDD C: (9/57) 3.1/2.45E-02	**All** L: (62/286) 1.4/2.26E-02 **Gender** Male L: (59/253) 1.5/7.76E-03 **Gender/Dx** M-BP L: (24/91) 1.9/2.78E-03 M-SZA C: (39/108) 1.5/1.55E-02	Autism Intellect Methamphetamine Psychosis SZ BP MDD Suicide		31
SPON2 Spondin 2	218638_s_at	(D) DE/6 93.8%	2	Not stepwise	**Gender/Dx** F-PTSD C: (3/7) 1/1.69E-02	**All** L: (14/234) 0.66/2.24E-02 **Gender** Male L: (14/206) 0.66/2.19E-02 **Gender/Dx** M-BP L: (10/77) 0.67/4.20E-02 M-MDD C: (5/49) 0.83/8.70E-03 L: (2/27) 0.88/3.93E-02	**All** L: (62/286) 1.6/8.58E-03 **Gender** Male L: (59/253) 1.7/4.62E-03 **Gender/Dx** M-BP L: (24/91) 4.4/9.90E-04 M-MDD L: (4/32) 14.6/1.88E-02	Autism BP Panic disorder SZ		31
ANK2 Ankyrin 2	202921_s_at	(I) DE/4 52.9%	2	**1.09E-02/4 Nominal**	**Gender** Female C: (13/60) 0.66/4.33E-02 F-BP C: (6/22) 0.75/3.84E-02 M-MDD C: (6/35) 0.72/4.81E-02	**Gender/Dx** M-MDD C: (5/49) 0.75/3.22E-02 L: (2/27) 0.96/1.66E-02	**Gender/Dx** M-MDD L: (4/32) 76.8/8.14E-03	Autism Alcohol BP Longevity ASD Chronic fatigue syndrome MDD Suicide SZ	Antidepressants	30
LAIR2 Leukocyte Associated Immunoglobulin Like Receptor 2	207509_s_at	(D) DE/6 98.5%	0	Not stepwise	**Most reproducibly predictive for state All** C: (38/258) 0.62/1.15E-02 **Gender** Female C: (13/60) 0.81/3.37E-04 L: (5/33) 0.81/1.36E-02 **Gender/Dx** F-BP C: (6/22) 0.86/4.94E-03 L: (2/12) 1/1.58E-02 F-PTSD C: (3/7) 1/1.69E-02 M-MDD C: (6/35) 0.76/2.44E-02	**Gender** Female C: (2/46) 0.97/1.36E-02	**Gender/Dx** M-BP L: (24/91) 2.6/7.13E-03 M-MDD L: (4/32) 5.5/4.21E-02	Suicide	Antidepressants	30
SUMO1 Small Ubiquitin-Like Modifier 1	208762_at	(D) DE/4 56.3%	9	Not stepwise	**Gender** Female C: (13/60) 0.70/1.46E-02 **Gender/Dx** F-BP C: (6/22) 0.75/3.84-02 L: (2/12) 0.9/4.28-02	**Gender/Dx** M-SZ C: (3/74) 0.87/1.57-02 L: (1/44) 1/4.52-02	**Gender/Dx** M-SZ C: (13/93) 2.98/2.98-02 L: (8/56) 3.26/3.07-02	Aging BP SZ		30
MKL2 MKL1/Myocardin Like 2	1562497_at	(I) AP/4 60.8%	2	**4.58E-02/4 Nominal**		**Most reproducibly predictive for trait first year All** C: (32/398) 0.59/3.79E-02 **Gender** Male C: (30/352) 0.61/2.53E-02 L: (14/206) 0.64/4.33E-02 **Gender/Dx** M-BP L: (10/77) 0.67/3.81E-02 M-MDD L: (2/27) 0.88/3.93E-02 M-PSYCHOSIS C: (8/161) 0.68/3.94E-02	**All** C: (113/474) 1.2/7.86E-03 L: (62/286) 1.4/3.45E-03 **Gender** Male C: (106/421) 1.2/1.84E-02 L: (59/253) 1.3/7.90E-03 **Gender/Dx** M-BP C: (41/140) 1.3/3.59E-03 L: (24/91) 1.6/6.70E-04 M-MDD L: (4/32) 3.3/1.73E-02	Autism SZ		29
DMGDH Dimethylglycine Dehydrogenase	231591_at	(I) DE/2 45.6%	4	**3.36E-02/4 Nominal**	**Gender/Dx** F-BP C: (6/22) 0.77/2.76E-02	**Gender/Dx** M-SZ L: (1/44) 1.0/4.52E-02	**Gender** Male L: (59/255) 1.3/4.80E-02 **Gender/Dx** M-BP L: (24/91) 1.6/2.89E-02 M-PSYCHOSIS C: (52/201) 1.3/1.69E-02 M-SZ C: (13/93) 1.4/2.67E-02 L: (8/56) 2.8/1.52E-02	Delusion Suicide		27
N4BP2L2 NEDD4 Binding Protein 2 Like 2	214388_at	(I) DE/4 69.1%	4	**4.40E-02/4 Nominal**	**Gender/Dx** F-BP C: (6/22) 0.77/2.76E-02 L: (2/12) 0.95/2.66E-02	**Gender/Dx** M-BP L: (10/77) 0.74/7.66E-03 M-SZ C: (3/74) 0.82/3.02E-02	**Gender/Dx** M-BP L: (24/91) 1.5/1.13E-02	BP MDD SZ Suicide		27
PCDHB6 Protocadherin Beta 6	239443_at	(I) DE/2 38.2%	6	**1.17E-02/4 Nominal**	**All** C: (38/258) 0.61/1.31E-02 **Gender** Male C: (25/198) 0.65/7.19E-03 **Gender/Dx** M-BP C: (10/101) 0.67/4.20E-02		**Gender/Dx** M-PSYCHOSIS L: (29/121) 1.5/1.51E-02 M-SZ L: (8/56) 1.8/1.98E-02	Suicide		27
SNCA Synuclein Alpha	215811_at	(D) AP/2 37.5%	11	Not stepwise	**Gender/Dx** M-PSYCHOSIS L: (2/24) 0.98/1.41E-02 M-SZ L: (2/15) 1/1.36E-02		**Gender/Dx** M-SZA C: (39/108) 1.6/3.62E-02	Alcohol Aggression Alzheimer’s BP MDD Methamphetamine Parkinson Suicide SZ	Omega-3 fatty acids Mood stabilizers	27
GJB2 Gap Junction Protein Beta 2	223278_at	(I) DE/2 48.5%	6	**2.42E-02/4 Nominal**	**Gender/Dx** M-MDD C: (6/35) 0.82/7.12E-03		**Gender/Dx** M-SZ L: (8/56) 2.2/2.37E-02	MDD	Antipsychotics	26
HIF1A Hypoxia Inducible Factor 1 Alpha Subunit	238869_at	(I) DE/4 54.4%	4	**1.11E-02/4 Nominal**			**Most reproducibly predictive for trait all future All** C: (113/474) 1.2/3.86E-02 L: (62/288) 1.5/1.28E-02 **Gender** Male C: (106/421) 1.2/1.42E-02 L: (59/255) 1.5/5.53E-03 **Gender/Dx** M-BP L: (24/91) 1.5/3.84E-02 M-PSYCHOSIS C: (52/201) 1.3/1.91E-02 L: (29/121) 1.7/2.57E-02 M-SZ C: (13/93) 1.7/3.44E-02 L: (8/56) 3.3/1.75E-02	Alcohol Autism BP MDD Longevity Pain SZ	EZN 2968	26
PSD3 Pleckstrin And Sec7 Domain Containing 3	218613_at	(D) AP/6 100%	2	Not stepwise		**Gender** Female C: (2/46) 0.98/1.18E-02	**Gender** Female C: (7/53) 2.2/4.42E-02	Autism Alcohol ASD BP SZ MDD Methamphetamine Chronic fatigue syndrome Suicide	Antipsychotics	26
STX11 Syntaxin 11	210190_at	(D) DE/2 49.2%	4.5	**2.74E-02/4 Nominal**	**Gender/Dx** M-MDD C: (6/35) 0.74/3.64E-02	**Gender/Dx** M-MDD C: (5/49) 0.95/4.78 -04	**Gender/Dx** M-MDD C: (9/57) 3.1/2.45E-02		Antidepressants Mood stabilizers	25.5
APOL3 Apolipoprotein L3	221087_s_at	(D) AP/4 50%	2	**2.96E-02/4 Nominal**		**All** L: (14/234) 0.7/5.34E-03 **Gender** Male L: (14/206) 0.71/4.53E-03 **Gender/Dx** M-MDD L: (2/27) 0.92/2.59E-02	**Gender/Dx** F-SZA C: (3/12) 8.1/4.33E-02 M-MDD L: (4/32) 9.6/2.59E-02	ADHD Suicide SZ		25
ELMO2 Engulfment And Cell Motility 2	220363_s_at	(D) DE/4 60.0% (D) AP/4 54.7%	2	**1.30E-02/4 Nominal**		**Gender/Dx** M-MDD C: (5/49) 0.78/2.20E-02 L: (2/27) 0.92/2.59E-02	**All** L: (62/288) 1.44/3.31E-02 **Gender** Male L: (59/255) 1.39/4.91E-02 **Gender/Dx** M-MDD C: (9/57) 3.86/8.54E-03 L: (4/32) 6.07/3.64E-02 F-PSYCHOSIS L: (6/17) 2.36/4.48E-02	Suicide		25
UBE2E2 Ubiquitin Conjugating Enzyme E2 E2	225651_at	(D) DE/4 53.8%	4	**4.41E-02/4 Nominal**	**Gender** Female C: (13/60) 0.68/2.58E-02 F-BP C: (6/22) 0.76/3.27E-02		**Gender/Dx** M-PSYCHOSIS C: (52/201) 1.4/5.21E-03 M-SZA C: (39/108) 1.6/2.83E-03	Psychosis		25
FKBP5 FK506 Binding Protein 5	224840_at	(D) DE/2 41.5%	12	Not stepwise			**Gender/Dx** M-SZ L: (8/56) 3.4/3.84E-02	Alcohol Anxiety BP Depression MDD Pain Psychosis Unipolar Depression Suicide	Mood stabilizers Psychotherapy	24
HLA-DRB1 Major Histocompatibility Complex, Class II, DR Beta 1	209312_x_at	(D) DE/2 41.5%	4	**1.22E-02/4 Nominal**			**All** L: (62/286) 1.7/5.17E-03 **Gender** Male L: (59/253) 1.6/1.21E-02 **Gender/Dx** F-PSYCHOSIS C: (6/17) 3.1/2.62E-02 F-SZA C: (3/12) 39.3/4.08E-02 M-SZA C: (39/108) 1.4/2.18E-02 L: (21/65) 1.7/4.72E-02	Alcohol BP Longevity Alzheimer’s disease SZ Pain Panic Disorder	Apolizumab	24
LCP2 Lymphocyte Cytosolic Protein 2	244251_at	(D) DE/4 53.8%	3	**2.01E-02/4 Nominal**		**Gender** Male C: (30/352) 0.61/2.19E-02 **Gender/Dx** M-SZA C: (5/87) 0.85/4.09E-03 M-PSYCHOSIS C: (8/161) 0.78/3.90E-03	**Gender/Dx** M-SZ C: (13/93) 1.46/4.14E-02 L: (8/56) 2.17/2.38E-02	MDD		24
LRRC59 Leucine Rich Repeat Containing 59	222231_s_at	(D) DE/4 61.5%	2	**3.15E-02/4 Nominal**			**All** L: (62/286) 1.35/4.50E-02 **Gender** Male L: (59/253) 1.38/3.67E-02 **Gender/Dx** F-SZA C: (3/12) 56.1/4.25E-02	SZ	Valproate	24
FOXK2 Forkhead Box K2	220696_at	(I) DE/4 58.8% (I) AP/4 72.5%	2	**1.52E-02/4 Nominal**	**Gender** Female C: (13/60) 0.68/2.18E-02 L: (5/33) 0.88/3.89E-03 **Gender/Dx** F-BP C: (6/22) 0.76/3.27E-02 L: (2/12) 1/1.58E-02 F-PTSD C: (3/7) 1/1.69E-02		**Gender/Dx** M-SZ L: (8/56) 2.2/1.09E-02	Alcohol Autism Delusions Hallucinations Suicide		23
HLA-B Major Histocompatibility Complex, Class I, B	211911_x_at	(D) DE/4 52.3%	3	**4.85E-02/4 Nominal**		**Gender/Dx** M-MDD C: (5/49) 0.85/4.99E-03 L: (2/27) 1.0/1.03E-02	**All** L: (62/288) 1.65/4.74E-03 **Gender** Male L: (59/255) 1.66/4.25E-03 **Gender/Dx** M-MDD L: (4/32) 5.35/1.09E-02 M-BP L: (24/91) 1.76/1.10E-02			23
NKTR Natural Killer Cell Triggering Receptor	243055_at	(I) DE/4 50% (I) AP/2 43.1%	4	**1.24E-02/4 Nominal**			**All** C: (113/474) 1.4/9.52E-05** **Gender** Male C: (106/421) 1.4/1.43E-04** **Gender/Dx** M-BP C: (41/140) 1.6/5.56E-05** M-PSYCHOSIS C: (52/201) 1.3/1.06E-02 M-SZ C: (13/93) 1.7/5.58E-03 L: (8/56) 1.7/4.98E-02	Alcohol BP MDD Suicide SZ		23
PLEKHA5 Pleckstrin Homology Domain Containing A5	239559_at	(I) DE/2 35.3%	4	**3.33E-02/4 Nominal**		**Gender/Dx** M-SZ C: (3/74) 0.91/8.24E-03	**Gender** Male C: (106/421) 1.2/4.50E-02 **Gender/Dx** M-BP L: (24/91) 1.6/1.15E-02	BP Suicide		23
C1orf123 Chromosome 1 Open Reading Frame 123	203197_s_at	(D) DE/4 72.3%	2	**2.92E-02/4 Nominal**			**All** L: (62/288) 1.5/1.44E-02 **Gender** Female L: (3/33) 12.3/3.35E-02 **Gender** Male L: (59/255) 1.3/4.43E-02 F-PSYCHOSIS C: (6/17) 3.5/2.00E-02 M-MDD L: (4/32) 3/3.73E-02	Suicide		21
UQCC1 Ubiquinol-Cytochrome C Reductase Complex Assembly Factor 1	217935_s_at	(D) DE/2 38.5%	4	**3.33E-02/4 Nominal**	**Gender/Dx** M-BP C: (10/101) 0.72/1.18E-02	**Gender/Dx** M-SZ C: (3/74) 0.89/1.19E-02		BP Suicide		21
PCBP2 Poly(RC) Binding Protein 2	237374_at	(I) DE/2 35.3%	4.5	2.83E-02/4 Nominal	**Gender/Dx** F-BP C: (6/22) 0.89/3.19-03 L: (2/12) 1/1.58-02 M-SZ C: (4/29) 0.8/2.89-02			BP Suicide		17.5
DCTN5 Dynactin Subunit 5	209231_s_at	(D) DE/6 90.8%	2	Not stepwise			**Gender/Dx** F-PSYCHOSIS C: (6/17) 3.3/3.22E-02 M-SZ L: (8/56) 6.5/4.80E-03	BP Suicide		15
LOC105378349 Uncharacterized LOC105378349	241143_at	(D) AP/6 90.6%	0	Not stepwise	**Gender/Dx** M-PSYCHOSIS C: (5/47) 0.74/4.22E-02		**Gender/Dx** M-BP C: (41/140) 1.4/2.00E-02 M-MDD C: (9/57) 2.4/2.68E-02			14

After Step 4 Testing in independent cohorts for state and trait predictions. Telomere length (TL) was chosen as a literature based positive control/comparator. FKBP5 is the gene with the most consistent evidence across all steps in our work and a de facto positive control based on its extensive prior evidence in the field. NUB1 has two different probesets. For Step 4 Predictions, C—cross-sectional (using levels from one visit), L—longitudinal (using levels and slopes from multiple visits). In All, by Gender, and personalized by Gender and Diagnosis (Gender/Dx). Underlined—best predictor category as depicted in Fig. 2

*(D)- decreased in expression in high stress; (I)-increased in high stress. DE* differential expression, *ANOVA* analysis of variance, *AP* absent/present, *NS* non-stepwise in validation, *M* males, *F* females, *MDD* depression, *BP* bipolar, ROC AUC area under the receiver-operating characteristic curve, *SZ* schizophrenia, *SZA* schizoaffective, *OR* odds ratio, *PSYCHOSIS* schizophrenia and schizoaffective combined, *PTSD* post-traumatic stress disorder

**Significant after Bonferroni correction for the number of biomarkers tested for predictive ability

#### Predicting trait: future psychiatric hospitalization with stress as a symptom/reason for admission

We conducted analyses for predicting future psychiatric hospitalizations with stress as a symptom/reason for admission in the first year following each testing visit in subjects who had at least 1 year of follow-up in the VA system, in which we have access to complete electronic medical records. ROC analyses between genomic and phenomic marker measures (cross-sectional, longitudinal) at a specific testing visit and future hospitalization were performed as described above, based on assigning if subjects had been admitted to the hospital due to stress or not. Additionally, a one tailed *t* test with unequal variance was performed between groups of subject visits with and without future hospitalization with stress. Pearson R (one-tail) correlation was performed between hospitalization frequency (number of hospitalizations with stress divided by duration of follow-up) and marker levels. A Cox regression was performed using the time in days from the testing visit date to first hospitalization date in the case of patients who had been hospitalized or 365 days for those who did not. The hazard ratio was calculated such that a value >1 always indicates increased risk for hospitalization, regardless if the biomarker is increased or decreased in expression.

We also conducted a Cox regression and Pearson correlation analyses for all future hospitalizations with stress, including those occurring beyond 1 year of follow-up, in the years following testing (on average 5.76 years per subject, range 0.07–11.27 years), as these calculations, unlike the ROC and *t* test, account for the actual length of follow-up, which varied from subject to subject. The ROC and *t* test might in fact, if used, under-represent the power of the markers to predict, as the more severe psychiatric patients are more likely to move geographically and/or be lost to follow-up. The Cox regression was performed using the time in days from visit date to first hospitalization date in the case of patients who had hospitalizations with stress or from visit date to last note date in the electronic medical records for those who did not.

### Biological understanding

#### Pathway analyses

IPA (Ingenuity Pathway Analysis, version 24390178, Qiagen), David Functional Annotation Bioinformatics Microarray Analysis (National Institute of Allergy and Infectious Diseases) version 6.7 (August 2016), and Kyoto Encyclopedia of Genes and Genomes (KEGG) (through DAVID) were used to analyze the biological roles, including top canonical pathways and diseases (Table 3), of the candidate genes resulting from our work. We ran the pathway analyses for the combined 220 unique genes (232 probesets) that were nominally significant after validation. For Network analysis of the 220 unique genes, we performed STRING Interaction Network (https://string-db.org) by inputting the genes into the search window and performed Multiple Proteins *Homo sapiens* analysis.

**Table 3:** Biological pathway analyses

	DAVID GO functional annotation biological processes					KEGG pathways				Ingenuity pathways (fold change)		
A. Pathways	#	Term	Count	%	*P* value	Term	Count	%	*P* value	Top canonical pathways	*P* value	Overlap
220 Stress genes (*n* = 220 genes, 232 probesets)	1	Antigen processing and presentation of exogenous peptide antigen via MHC class I, TAP-dependent	8	3.7	9.30E-06	Antigen processing and presentation	8	3.7	9.80E-05	Antigen presentation pathway	1.71E-06	15.8% 6/38
	2	Proteasome-mediated ubiquitin-dependent protein catabolic process	12	5.6	3.10E-05	Viral myocarditis	7	3.3	1.50E-04	Natural killer cell signaling	2.67E-05	6.6% 8/122
	3	Negative regulation of T cell proliferation	6	2.8	7.10E-05	Lysosome	9	4.2	3.60E-04	Autoimmune thyroid disease signaling	1.02E-04	10.4% 5/48
	4	Protein K48-linked ubiquitination	6	2.8	2.30E-04	Epstein–Barr virus infection	11	5.1	1.20E-03	Graft-vs.-host disease signaling	1.02E-04	10.4% 5/48
	5	Antigen processing and presentation of peptide antigen via MHC class I	5	2.3	4.10E-04	Graft-vs.-host disease	5	2.3	1.70E-03	Phagosome maturation	1.02E-04	5.4% 8/148

B. Diseases	David					Ingenuity pathways disease		
	#	Term	Count	%	*P* value	Diseases and disorders	*P* value	# Molecules
220 Stress genes (*n* = 220 genes, 232 probesets)	1	HIV	10	4.7	1.10E-03	Cancer	9.75E-03–2.15E-07	202
	2	Drug-induced liver injury	4	1.9	2.70E-03	Organismal injury and abnormalities	9.75E-03–2.15E-07	206
	3	HIV infections|[X]human immunodeficiency virus disease	12	5.6	3.00E-03	Infectious diseases	8.66E-03–2.33E-06	53
	4	Malaria, cerebral|malaria, falciparum	3	1.4	3.00E-03	Inflammatory response	9.75E-03–3.06E-05	61
	5	Adrenal hyperplasia, congenital	3	1.4	4.00E-03	Metabolic disease	9.75E-03–6.01E-05	50

For validated biomarkers (*n* = 232 probesets, 220 genes)

#### CFG beyond Stress: evidence for involvement in other psychiatric and related disorders

We also used a CFG approach to examine evidence from other psychiatric and related disorders, for the list of top predictive biomarkers after Step 4 testing (*n* = 41) (Table S3).

### Therapeutics

#### Pharmacogenomics

We analyzed which of our individual top predictive biomarkers from Fig. 2 (*n* = 41 genes, 42 probesets) are known to be modulated by existing drugs using our CFG databases and using Ingenuity Drugs analyses (Table 2 and Table S4).

#### New drug discovery/repurposing

We also analyzed which drugs and natural compounds are an opposite match for the gene expression profiles of panels of our top predictive biomarkers, using the Connectivity Map (https://portals.broadinstitute.org, Broad Institute, MIT) (Table 4). One hundred and forty out of the nominally validated 232 probesets from Step 3 were present in the HGU-133A array used for the Connectivity Map. Out of these, we also compiled gene expression signatures of the probesets that were predictive in Step 4 (nominally significant) for all subjects, as well as separately for males and for females, and personalized by gender and diagnosis.

**Table 4:** New drug discovery/repurposing leads

A. CMAP analysis with nominally validated biomarkers (22 increased and 118 decreased were present in HG-U133A array used by Connectivity Map)		
Rank	cmap name	Score
6100	**cefotiam**	−1
6099	proguanil	−0.991
6098	hydroxyachillin	−0.96
6097	Prestwick-682	−0.95
6096	levopropoxyphene	−0.949
6095	**isoflupredone**	−0.943
6094	ozagrel	−0.941
6093	streptozocin	−0.938
6092	cyclopenthiazide	−0.934
6091	metformin	−0.93
6090	**corticosterone**	−0.925
6089	***calcium folinate***	−0.924
6088	diphenhydramine	−0.921

B. CMAP analysis with biomarkers that are nominally predictive in all (5 increased and 52 decreased were present in HG-U133A array used by Connectivity Map)		
Rank	cmap name	Score
6100	**ambroxol**	−1
6099	ozagrel	–0.971
6098	cefotiam	−0.959
6097	xamoterol	−0.951
6096	***betulin***	−0.93
6095	isometheptene	−0.927
6094	primidone	−0.925
6092	tocainide	−0.919
6093	diloxanide	−0.919
6089	alprostadil	−0.913
6090	amphotericin B	−0.913
6087	oxolamine	−0.909

C. CMAP analysis with biomarkers that are nominally predictive in males (5 increased and 48 decreased were present in HG-U133A array used by Connectivity Map)		
Rank	cmap name	Score
6100	**ozagrel**	−1
6099	flucloxacillin	−0.981
6098	ambroxol	−0.97
6097	dapsone	−0.958
6096	tiaprofenic acid	−0.955
6095	primidone	−0.939
6094	***betulin***	−0.936
6093	proguanil	−0.929
6092	gossypol	−0.925
6091	levopropoxyphene	−0.92
6090	xamoterol	−0.917
6089	streptozocin	−0.912
6088	tocainide	−0.909

D. CMAP analysis with biomarkers that are nominally predictive in females (9 increased and 21 decreased were present in HG-U133A array used by Connectivity Map)		
rank	cmap name	Score
6100	**flecainide**	−1
6099	Prestwick-682	−0.997
6098	spiramycin	−0.98
6097	domperidone	−0.974
6096	homatropine	−0.967
6094	isoniazid	−0.964
6095	proguanil	−0.964
6093	phentolamine	−0.958
6092	sulfamonomethoxine	−0.952
6091	**fludrocortisone**	−0.951
6090	dizocilpine	−0.946
6087	adiphenine	−0.942
6088	tolnaftate	−0.942

Connectivity Map [66] (CMAP) analysis—drugs that have opposite gene expression profile effects to the signature of our validated genes (A), and out of them, those that are also significant predictive biomarkers (B–D). A score of −1 indicates the perfect opposite match, i.e., the best potential therapeutic to decrease stress. Bold—top candidates. Bold and italic—natural compounds of interest. Bold and underlined—compounds known to modulate stress, which serve as reassuring positive controls

### Convergent Functional Evidence (CFE)

For the top predictive biomarkers (*n* = 42), we tabulated into a CFE score all the evidence from discovery (up to 6 points), prioritization (up to 12 points), validation (up to 6 points), testing (state, trait first-year Hospitalization with Stress visits, trait all future Hospitalization with Stress visits—up to 8 points each if significantly predicts in all subjects, 6 points if predicts by gender, 4 points if predicts in gender/diagnosis), other psychiatric and related disorders (3 points), and drug evidence (3 points). The total score can be up to 54 points: 36 from our data and 18 from literature data. We weigh our data twice as much as the literature data. The goal is to highlight, based on the totality of our data and of the evidence in the field to date, biomarkers that have all around evidence: track stress, predict it, are reflective of stress and other pathology, and are potential drug targets. Such biomarkers merit priority evaluation in future clinical trials.

## Results

### Step 1: Discovery of biomarkers for stress

We used a powerful within-subject longitudinal discovery approach to identify genes that: (1) change in expression in blood between low stress states (Life Stress VAS ≤ 33 out of 100) and high stress states (Life Stress VAS ≥ 67 out of 100), (2) track the stress state across visits in a subject, and (3) track stress state in multiple subjects. We used a longitudinally followed cohort of psychiatric subjects that showed diametric changes in stress states between at least two testing visits (*n* = 36 subjects) (Fig. 1 and Table 1). The stress state self-report may be more reliable in this cohort, as the subjects demonstrated the aptitude and willingness to report different, and diametric, stress states. Using our 33% of maximum raw score threshold (internal score of 2 pts, we had 12,884 unique probesets (Fig. 1d). These were carried forward to the prioritization step. This represents approximately a 4-fold enrichment of the 54,625 probesets on the Affymetrix array.

We also explored in the discovery cohort whether subtypes of stress can be identified based on mental state at the time of high stress visits, using two-way hierarchical clustering with anxiety, mood, and psychosis measures. We uncovered three potential subtypes of stress: predominantly anxious (possibly reflecting increased reactivity), predominantly psychotic (possibly reflecting dis-connectivity), and non-comorbid with other psychiatric symptoms (possibly reflecting better adaptation) (Fig. 1c). These subtypes need to be further evaluated and tested in independent cohorts for practical utility, diagnostic and therapeutic. Deeper analyses of the clustering in future studies may also substantiate further parsing of the subtypes into eight instead of only three subtypes.

### Step 2: Prioritization of biomarkers based on prior evidence in the field

We used a CFG approach to prioritize the candidate biomarkers identified in the discovery step (33% cutoff, internal score of ≥2 pts) by using all the published prior independent evidence in the field (Fig. 1e). There were 3590 probesets that had a CFG score (combined internal and external score) of ≥6. These were carried forward to the validation step. This represents approximately a 15-fold enrichment of the probesets on the Affymetrix array.

### Step 3: Validation of biomarkers for severe stress state and trait

Next, we validated these prioritized candidate biomarkers (*n* = 3590), in a demographically matched cohort of psychiatric subjects with clinically severe state and trait stress, by assessing which markers were stepwise changed in expression from low stress in the discovery cohort to high stress in the discovery cohort to clinically severe in the independent validation cohort (Fig. 1f). These genes are likely involved in stress state *and* trait. Two thousand two hundred and twenty-eight probesets were non-stepwise changed, 1130 were stepwise changed, and 232 were nominally significant by ANOVA. This represents approximately a 235-fold enrichment of the probesets on the Affymetrix array. The best *p* value increased in expression (risk) biomarker was NUB1 (Negative Regulator of Ubiquitin-Like Proteins 1) (*p* = 0.00062), and the best *p* value decreased in expression (protective) biomarker was ASCC1 (*p* = 0.00028). The Bonferroni threshold was set conservatively at 0.05/3590 = 0.000014, and none of the biomarkers crossed that threshold.

### Step 4: Testing for diagnostics

We carried forward into testing the top biomarkers from each of the first three steps. The list of candidate biomarkers thus includes the top biomarkers from discovery step (≥90% of scores, *n* = 39), the top biomarkers after the prioritization step (total CFG score≥13, *n* = 21), and the nominally significant biomarkers after the validation step (*n* = 232), for a total of *n* = 285 probesets (*n* = 269 genes) (Fig. 1). The rationale for that was that there might be biomarkers that did not survive validation in our particular cohort and stringent stepwise change in expression approach but have either an abundance of evidence from the literature supporting their involvement in stress and thus are highly prioritized at Step 2 and/or have strong evidence in the discovery Step 1 and might be completely novel candidate biomarkers for stress.

We tested whether the 285 candidate biomarkers are able to predict stress severity state, and future psychiatric hospitalizations with stress, in another independent cohort of psychiatric subjects. We used biomarker levels information cross-sectionally, as well as expanded longitudinal information about biomarker levels at multiple visits, as predictors. We tested the biomarkers in all subjects in the independent test cohort, as well as in a more personalized fashion by gender and psychiatric diagnosis, showing increased accuracy with the personalized approach, in particular in women (Fig. 2). In general, the longitudinal information was more predictive than the cross-sectional information.

Across all subjects tested, NUB1, the top risk biomarker after validation, was also the best predictor for high stress state (area under the ROC curve (AUC) 65%, *p* = 0.0014). NUB1 was an even better predictor of stress state by gender in females (AUC 74%, *p* = 0.004) and by gender and diagnosis in female bipolars (AUC 78%, *p* = 0.02). NUB1, which was increased in expression in High Stress states in our studies, has previous convergent evidence for increase in expression in stress, in human brain in individuals exposed to social isolation before dying [10] and in blood in individuals exposed to combat traumas, as reported by Breen et al. [11]. It also has evidence for increase in expression in the brain of mice subjected to chronic variable stress by Nestler and colleagues [12]. Such reproducibility across studies, tissues, and populations provides strong reasons to consider it as a bona fide marker for psychological stress, and it serves as a reassuring de facto positive control for the design and power of our study. Interestingly, NUB1 is also increased in expression in our previous blood biomarker studies of suicide in both males [5, 4] and females [6] (Table S3). There is a strong clinical connection between stress and suicide.

APOL3 was the best predictor for trait first-year future hospitalizations with stress (AUC 70%, *p* = 0.0053). APOL3 was an even better predictor of first-year future hospitalizations in males (AUC 71%, *p* = 0.045) and by gender and diagnosis in male depression (AUC 92%, *p* = 0.026). It also is a good predictor of all future hospitalizations with stress in male depression (odds ratio (OR) 9.6, *p* = 0.026). APOL3 (Apolipoprotein L3), decreased in expression in High Stress states in our studies, has previous convergent evidence for decrease in expression in the brain in mice subjected to stress [13]. Interestingly, APOL3 is also decreased in expression in our previous blood biomarker studies of suicide in both males [5] and females [6] (Table S3).

MAD1L1 (Mitotic Arrest Deficient Like 1) the best predictor for trait all future hospitalizations with stress (OR 1.80, *p* = 0.0013). MAD1L1 was an even better predictor by gender and diagnosis in male bipolar (OR 2.1, *p* = 0.0097) and male depression (OR 31.4, *p* = 0.0055). MAD1L1, which is decreased in expression in High Stress states in our studies, has previous convergent evidence for decrease in expression in blood in chronic stress [14]. Of note, MAD1L1 has strong previous genetic and gene expression data for involvement in autism [15], as well as in bipolar disorder [16] and schizophrenia [17]. It may mediate the impact of stress on those disorders.

NKTR (Natural Killer Cell Triggering Receptor) (OR 1.37, *p* = 0.000095) survived Bonferroni correction for all the 285 biomarkers tested. Importantly, NKTR, increased in expression in blood in High Stress states in our studies, was also reported increased in expression in blood in studies of social isolation in humans [18] and in brain in studies of chronic variable stress in mice by Nestler and colleagues [12]. NKTR is also increased in expression in our previous blood biomarker studies of suicide in both males [5, 4] and females [6], as well as increased in expression in postmortem brain studies in depression [19] and in schizophrenia [20] (Table S3), possibly underlying the effect of stress in those disorders.

By gender, in females, FOXK2 was the best predictor for state (AUC 88%, *p* = 0.0039), PSD3 the best predictor for trait first-year hospitalizations (AUC 98%, *p* = 0.011), and C1orf123 for trait all future hospitalizations (OR 12.26, *p* = 0.033). In males, PCDHB6 was the best predictor for state (AUC 65%, *p* = 0.0072), APOL3 the best predictor for trait first-year hospitalizations (AUC 71%, *p* = 0.0045), and MAD1L1 the best predictor for trait all future hospitalizations (OR 1.7, *p* = 0.0027).

Personalized by gender and diagnosis, in female bipolar CIRBP was a strong predictor for state (AUC 100%, *p* = 0.016) and in female schizoaffective HLA-DRB1 for trait all future hospitalizations (OR 39.23, *p* = 0.041). In male schizophrenia, SNCA was a strong predictor for state (AUC 100%, *p* = 0.014), in male depression STX11 was a strong predictor for trait first-year hospitalizations (AUC 100%, *p* = 0.00047), and in male depression ANK2 was a strong predictor for trait all future hospitalizations (OR 76.81, *p* = 0.0081). Owing to the smaller size of these gender and diagnosis cohorts, these results need to be considered preliminary and interpreted with caution.

TL, used as a comparator/positive control, was a good predictor for stress state and first-year hospitalizations, particularly in males with depression (Table 2). There is an extensive prior literature documenting the effects of stress on TL from Blackburn, Epel, and colleagues [21, 22], as well as other investigators [23–25].

Across all subjects tested and in males, predictions of future hospitalizations with stress were in general somewhat stronger using phenotypic markers (such as the PTSD PCL-C scale and the VAS Stress scale) than biomarkers, but predictions were stronger using biomarkers than phenotypic markers in females and personalized by gender and diagnosis. Also, panels of the 232 validated biomarkers did not work as well as individual biomarkers, particularly when the latter are tested by gender and diagnosis, consistent with there being heterogeneity in the population and supporting the need for personalization (Supplementary Information- Complete Datasets and Analyses).

### Step 5: Biological roles

Fifth, we assessed whether our top predictive biomarkers have evidence for involvement in other psychiatric and related disorders (Table 2 and S3). A majority of our biomarkers have some evidence in other psychiatric disorders, consistent with the broad effect of stress on the brain and on mind domains/dimensions [26–29], whereas a few seem to be specific for stress, such as HLA-B (Major Histocompatibility Complex, Class I, B), LOC105378349 (Uncharacterized LOC105378349), and STX11 (Syntaxin 11). More than half of our top predictive biomarkers (26 out of the 41 genes, i.e., 63%) have prior evidence for involvement in suicide, suggesting an extensive molecular co-morbidity between stress and suicide, to go along with the clinical and phenomenological co-morbidity [5–7].

We also analyzed the biological pathways and networks our nominally validated biomarkers (*n* = 232 probesets 220 genes) are involved in. The top biological pathway is involved in antigen processing and presentation (Table 3), broadly speaking in the reaction to threats. The pathways are shared with other non-psychiatric diseases, suggesting that stress is a whole-body disease [30]. There is a network centered on HLA DRB1 that may be involved in reactivity/immune response. A second network is centered on HDAC3 and may be involved in activity/trophicity. A third network is centered on RAC1 and may be involved in connectivity/signaling. ACTR1A seems to be a nodal gene connecting these three networks (Figure S2).

### Step 6: Targeted treatments and drug repurposing

Sixth, we analyzed which of our top predictive biomarkers have evidence for being directly or indirectly modulated by existing drugs, in the opposite direction to their change in stress (Table 2 and Table S4), using our CFG literature databases. Some biomarkers are modulated by omega-3 fatty acids, some by antidepressants, some by mood stabilizers, some by antipsychotics, and some by other treatments such as psychotherapy and meditation. This opens avenues for future studies of pharmacogenomic stratification of patients with, for example, PTSD, to various treatments or treatment combinations and for objectively measuring the response to treatment.

We also used the validated biomarker signature, and out of them, the top predictive biomarkers gene expression signatures, to interrogate the Connectivity Map database from Broad/MIT to identify leads to potential drugs and natural compounds that have the opposite effects on gene expression to stress and can be repurposed for treating stress (Table 4). The top drugs and nutraceuticals identified as potential new stress therapeutics using the validated biomarkers from Step 3 are cefotiam (an antibiotic) and calcium folinate (a B vitamin). While primarily utilized for their antimicrobial activity, β-lactam antibiotics like cefotiam were found to promote the expression of the glutamate transporter GLT1 and have a neuroprotective role in vivo and in vitro when used in models of ischemic injury and motor neuron degeneration, suggesting significant neuroprotective properties [31]. A study investigating the effects of cephalosporin in a mouse model of major depressive disorder, ceftriaxone, of the cephalosporin family, was shown to exhibit antidepressant properties increasing glutamate uptake, thought to be impaired in major depressive disorder [32]. Calcium folinate is a derivative of folate. Folate has been implicated in neurotransmitter metabolism and has been suggested as a therapeutic option in depression and negative symptoms schizophrenia [33].

Additionally, ambroxol (originally a mucolytic drug, with recent evidence as sodium channel blocker with anti-pain properties [34]) and betulin (a triterpene compound from the bark of the birch tree, with evidence for anxiolytic effects [35]) were identified using the smaller list of biomarkers that are predictive in all in Step 4. Furthermore, ozagrel (an antiplatelet agent working as a thromboxane A2 synthesis inhibitor) was identified using the biomarkers that are predictive in males, and flecainide (an antiarrhythmic agent that blocks sodium channels) using the biomarkers that are predictive in females. It is not unprecedented for drugs from other fields, and natural compounds, to be repurposed for novel indications, see recent examples for aging [36, 37].

In the latter, the antibiotic minocycline was shown to enhance longevity and proteostasis in old post-stress responsive experimental model organisms (*Caenorhabditis elegans*). That work provides a geroprotective mechanism for the beneficial effects of tetracyclines in models of neurodegenerative disease [37].

### Step 7: Convergent Functional Evidence

The biomarkers with the best overall CFE across the six steps were FKBP5 (FK506 Binding Protein 5), DDX6 (DEAD-Box Helicase 6), B2M (Beta-2-Microglobulin), LAIR1 (Leukocyte Associated Immunoglobulin Like Receptor 1), RTN4 (Reticulon 4), and the previously discussed NUB1 (Table 2). FKBP5, a decreased in expression biomarker, survived discovery, prioritization, and validation. It seems to be a better predictor for state in females and for trait in males, especially personalized by diagnosis. FKBP5 has independently been described as decreased in expression in blood in World Trade Center attack survivors [38] and in a Dutch cohort with post-deployment PTSD [39], as well as in the postmortem brains from PTSD [40]. It has been previously well established as a risk gene for stress disorders by multiple groups, including seminal studies by Binder, Ressler, and colleagues [41, 42] (Table S2). FKBP5 appearance in our screen is reassuring and serves as a de facto positive control for our approach. It is also involved in multiple other psychiatric disorders, consistent with the role of stress as a trigger or precipitant of illness (Table S3). There is previous evidence for its modulation in expression in opposite direction to stress by mood stabilizers (Table S4), and interestingly, by psychotherapy [43]. DDX6, an increased in expression biomarker, has previous convergent evidence of being increased in expression in blood [44] and in amygdala [28] of mice subjected to stress. It is a strong predictor of state and trait stress across all, by gender, and by gender and diagnosis. DDX6 has also been implicated in other neuropsychiatric disorders (alcoholism, other addictions, depression, schizophrenia), as well as is an increased in expression blood biomarker for suicide in our previous studies [7]. LAIR1, a decreased in expression biomarker, survived discovery, prioritization, and validation. It has previous convergent evidence from human studies of being decreased in expression in blood in PTSD related to childhood trauma [45] and to interpersonal trauma in females [11]. It is a strong predictor of state stress in females and of trait stress across all and in males. LAIR1 is also a decreased in expression blood biomarker for suicide in our previous studies [7]. RTN4, an increased in expression biomarker, has previous convergent evidence of being increased in the nucleus accumbens (NAC) in social isolation in humans [10] and in blood in PTSD [46, 47, 45]. It is decreased in expression in blood by treatment with the nutraceutical omega-3 fatty acid DHA in stressed female mice in independent studies from our group [29], as well as by valproate in the brain of mice [48]. RTN4 is a predictor of trait future hospitalizations with stress in all, as well as separately in males and females. RTN4 has also been implicated in bipolar disorder, alcoholism, and pain, as well as is an increased in expression suicide blood biomarker in our studies [7]. B2M, an increased in expression biomarker, has previous convergent evidence of being increased in the NAC in social isolation in humans [10], and it is decreased in expression in NAC by treatment with the nutraceutical omega-3 fatty acid DHA in stressed female mice in independent studies from our group [29]. It is a strong predictor of state stress in females with psychotic disorders and of future hospitalizations with stress in both genders. B2M has also been implicated in other neuropsychiatric disorders (alcoholism, autism, depression, eating disorders, pain, as well as aging and suicide), possibly mediating the effects of stress in those disorders.

## Discussion

Biomarkers are emerging as important tools in disorders where subjective self-report of an individual and/or clinical impression of a healthcare professional are not always reliable. Recent work by our group has identified blood gene expression biomarkers that track suicidality using powerful longitudinal within-subject designs, validated them in suicide completers, and tested them in independent cohorts demonstrating their ability to predict state (suicidal ideation) and to predict trait (future hospitalizations for suicidality) [5, 2, 6, 49]. Similar to suicidality, psychological stress is a subjective feeling, with objective roots. It may reflect past or current traumatic events, their adverse consequences, and compensatory mechanisms. Metabolic and hormonal changes may be an informative but incomplete window into the underlying biology [50].

We present work describing a powerful longitudinal within-subject design [4–7, 49, 51, 52] in individuals to discover blood gene expression changes between self-reported low stress and high stress states. The longitudinal within-subject design is relatively novel in the field and has shown power with very small *N*s [4–7, 49, 51, 52], as also illustrated and discussed by Snyder and colleagues [53], as well as by Schork and colleagues [54, 55]. Human studies, particularly genetic ones that use a case–control design, are susceptible to the issue of being underpowered. We estimate, based on our previous body of work in genetics and gene expression, that gene expression studies, by integrating the effects of many SNPs and environment, are up to three orders of magnitude more powerful than genetic studies. We also estimate based on previous work that a within-subject design is up to three orders of magnitude more powerful than a case–control design. In toto, our approach may be up to 6 orders of magnitude more powerful than a genetic case-control design (GWAS), hence a cohort of ≈10^1^ for within-subject discovery may be powered to the equivalent of a GWAS with ≈10^6^ subjects. In fact, recent results described for a large GWAS of PTSD in veterans carried out by Stein, Gelernter and colleagues [56] implicate the genes CAMKV, KANSL1, possibly CRHR1, and TCF4 (as discussed in Duncan et al. 2018 [57]). Three of these genes (KANSL1, CRHR1, and TCF4) have functional evidence for tracking stress in our within-subject discovery (Step 1), passing our preset threshold (see Supplementary Information- Complete Data and Analyses), and being carried forward into prioritization (Step 2) and validation (Step 3). CRHR1 was also nominally significant after validation. This convergence of independent findings using independent approaches in independent populations is reassuring. We believe that, because: (1) we are using a within-subject design for discovery, analyzing gene expression, which is closer to the phenotype, (2) are using a CFG to prioritize findings, integrating our data with other lines of evidence in the field (from human and animal model studies), (3) are validating our biomarkers in a clinically severe population, and (4) are testing them for both state and trait predictive ability in independent cohorts, we are getting reasonably robust and reproducible results for the field to follow-up on. It has to be noted that our cohort sizes are comparable to our published gene expression studies in suicide, which had a similar design, and were successful in identifying biomarkers that were predictive [4–7] and independently discovered and/or validated by other investigators [52, 58–62].

Some of these candidate gene expression biomarkers are increased in expression in high stress states (being putative risk genes), and others are decreased in expression (being putative protective/resilience genes). We cannot readily differentiate with our observational studies which of them are a reflection of damage and which are compensatory mechanisms. However, given the fact that these biomarkers are discovered in Step 1 by tracking present/state changes in the perception of stress and not past/trait exposure, they are more likely a reflection of pathogenesis rather than adaptation.

Our systematic approach led to the identification of objective predictive biomarkers for stress, state, and trait. We present evidence for universal biomarkers for stress, as well as show evidence that personalization by gender and diagnosis enhances precision, going from AUCs >60% to AUCs >80%. Earlier studies in mice by us [28, 29] and by Yehuda and colleagues [44] had indicated as well profound sex differences in brain/blood gene expression patterns in stress. More than half of the top predictive markers we have identified overlap with markers previously identified by us in suicide, and the majority of markers have evidence in other psychiatric disorders, underlying the toxic impact of stress on mental health. These biomarkers may permit novel patient stratifications for treatments, such as the possible use of lithium in patients with changes in TL, FKBP5, OAS1, SNCA, and STX11, as well as the use of omega-3 fatty acids in patients with changes in TL, RTN4, SNCA, and B2M (Table S4). The biomarker gene expression signatures also open the door to drug repurposing approaches, including other nutraceuticals such as folate, already used in depression [63] and schizophrenia [64], both of which are disorders eminently susceptible to stress, and betulin, which also has other metabolic and cardiovascular health benefits [65]. Nutraceuticals are particularly amenable to use in preventive population-level approaches. In conclusion, our studies identified new biological underpinnings of psychological stress and provide important leads toward novel diagnostics and targeted therapeutics for devastating stress–related disorders, such as PTSD.

## Supplementary information

Supplementary Information - Figures S1-S2 and Tables S2-S4

Supplementary Information - Detailed Demographics Table S1

Supplementary Information- Complete Data and Analyses
