# Supplementary material for: Towards precision medicine for stress disorders: diagnostic biomarkers and targeted drugs
Source: Mol Psychiatry. 2019 Mar 12;25(5):918–38. doi: 10.1038/s41380-019-0370-z (PMC7192849; doi:10.1038/s41380-019-0370-z)
Supplement: Supplementary file 1 — Supplementary Information - Figures S1-S2 and Tables S2-S4 [file 41380_2019_370_MOESM1_ESM.docx]

**Supplementary Information**

**Figure S1: Simplified Stress Scale** (VAS 0-100)


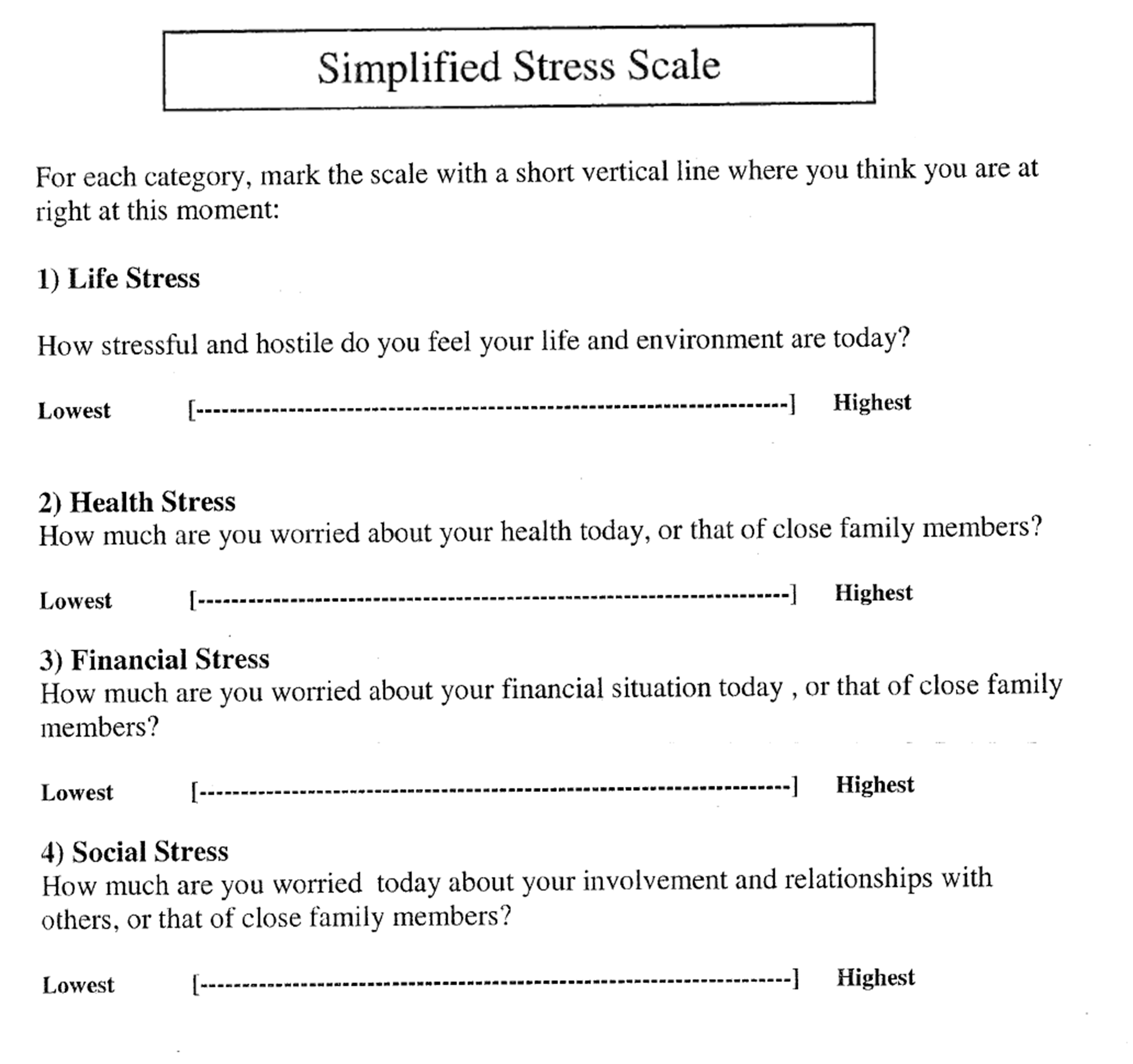


**Figure S2:** [STRING Interaction Network](http://version10.5.string-db.org/newstring_cgi/show_network_section.pl?limit=0&targetmode=proteins&caller_identity=gene_cards&network_flavor=evidence&identifiers=9606.ENSP00000414303%0d%0a9606.ENSP00000397297%0d%0a9606.ENSP00000304669%0d%0a9606.ENSP00000339007%0d%0a9606.ENSP00000353483%0d%0a9606.ENSP00000260227%0d%0a9606.ENSP00000401303%0d%0a9606.ENSP00000340698%0d%0a9606.ENSP00000263967%0d%0a9606.ENSP00000265164%0d%0a9606.ENSP00000244007%0d%0a9606.ENSP00000269141%0d%0a9606.ENSP00000358525%0d%0a9606.ENSP00000274335%0d%0a9606.ENSP00000314458%0d%0a9606.ENSP00000178640%0d%0a9606.ENSP00000321209%0d%0a9606.ENSP00000250559%0d%0a9606.ENSP00000352157%0d%0a9606.ENSP00000264554%0d%0a9606.ENSP00000269305%0d%0a9606.ENSP00000348786%0d%0a9606.ENSP00000431418%0d%0a9606.ENSP00000356056%0d%0a9606.ENSP00000308938%0d%0a9606.ENSP00000364995%0d%0a) for nominally validated biomarkers for stress (n=220 genes, 232 probesets)

**
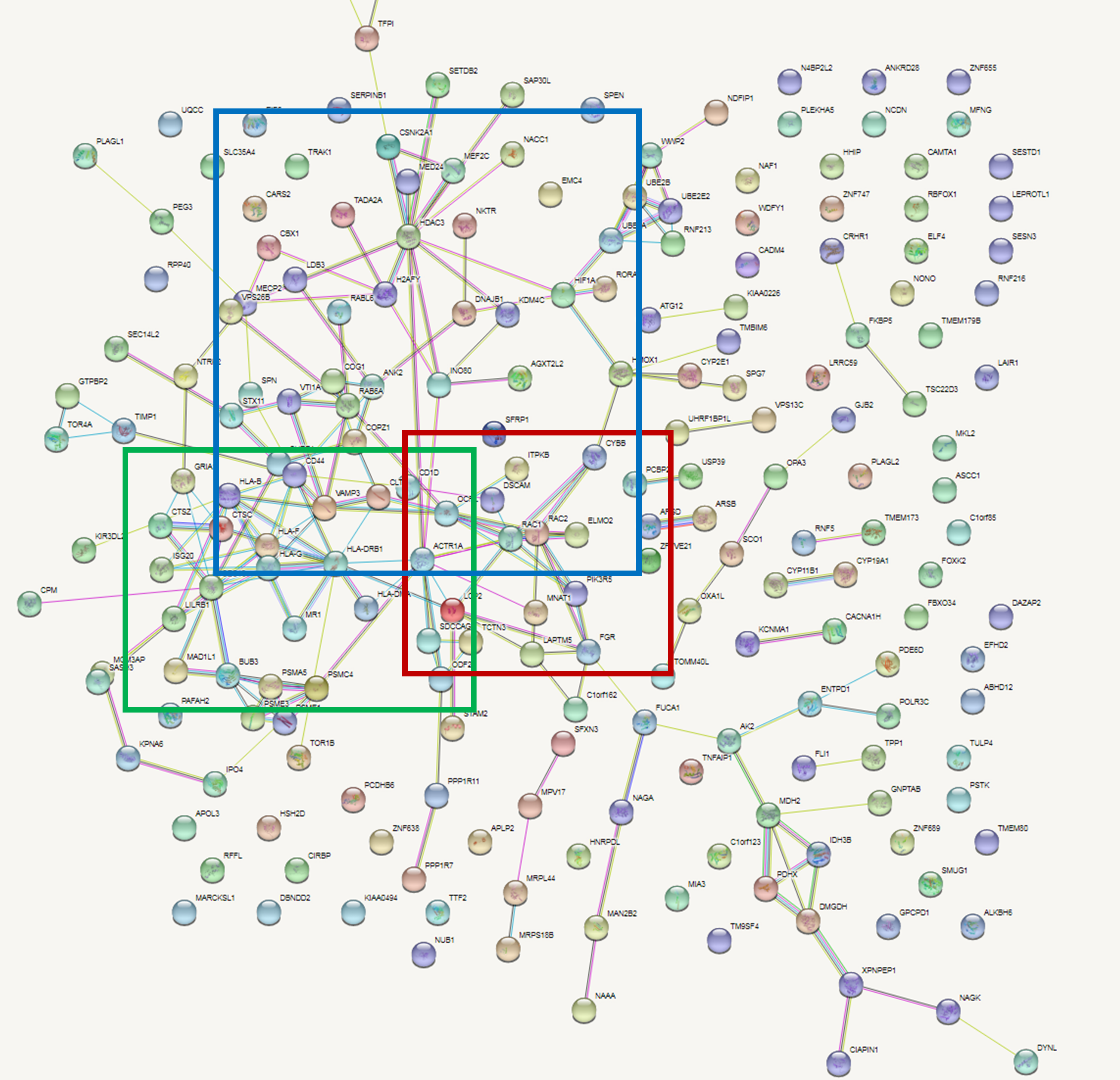
**

**Table S2. Evidence for involvement in Stress for Top Predictive Biomarkers for Stress (from Figure 2) (n=41 genes, 42 probesets).** Red- increased in expression (I) in Stress, Blue- decreased in expression (D). DE- differential expression, AP-Absent/Present.

| **Gene Symbol/ Gene Name** | **Probeset** | **Discovery** (Change) Method/Score  6pts | Prior human genetic evidence for Stress 2pts. | Prior human Brain expression evidence for Stress 4 pts | Prior human peripheral evidence for Stress | Prior Non-human genetic evidence for Stress 1pt. | Prior Non-human Brain expression evidence for Stress 2pts. | Prior Non-human peripheral evidence for Stress 1pt | **Prioritization Total CFG Score For Stress** | **Validation Anova p-value  6 pts** |
| --- | --- | --- | --- | --- | --- | --- | --- | --- | --- | --- |
| **TL**  Telomere Lenght |  | (D) | **Epigenetic PTSD and Traumatic stress**  [^1^](#_ENREF_1) |  | (D) **Trauma and PTSD Leukocytes**  [^2^](#_ENREF_2)  (D) **Veteran’s Early Trauma, Stress** severity granulocytes  [^3^](#_ENREF_3) |  | (D)  mPFC **maternal Stress** [^4^](#_ENREF_4)  (D)  Meerkats pup Tail Skin **Early-life competiction** [^5^](#_ENREF_5) | (D) **PTSD-like Ratmodel**  Blood Leukocytes [^6^](#_ENREF_6) | 7 | NS |
|  |  |  |  |  |  |  |  |  |  |  |
| **FKBP5** FK506 Binding Protein 5 | 224856_at | (D) DE/4 53.8 % | **PTSD**  [^24^](#_ENREF_24),[^27^](#_ENREF_27),[^28^](#_ENREF_28)  **Childhood Trauma** [^29^](#_ENREF_29),[^30^](#_ENREF_30)  **Psychological Stress**  [^31^](#_ENREF_31),[^32^](#_ENREF_32)  **PTSD**  [^27^](#_ENREF_27),[^33^](#_ENREF_33),[^34^](#_ENREF_34),[^35^](#_ENREF_35),[^36^](#_ENREF_36) | (D) PFC **PTSD** [^37^](#_ENREF_37) | (D)  PBMCs **Post-Deployment PTSD** [^29^](#_ENREF_29)  (I)  whole blood RNA **PTSD** [^38^](#_ENREF_38)  DE Blood **PTSD**  [^27^](#_ENREF_27)  (I) PBMC **PTSD**  [^39^](#_ENREF_39)  (D) Whole Blood **PTSD**  [^40^](#_ENREF_40)  (D) Venous Blood **PTSD**  [^14^](#_ENREF_14)  (I) Fasting Blood **PTSD**  [^40^](#_ENREF_40)  (D) PBMC **Psychological Stress**[^41^](#_ENREF_41)  (D) **Relaxation Response**  [^42^](#_ENREF_42)  (D) leukocytes **Social Isolation**  [^43^](#_ENREF_43)  (I) Blood **Psychotherapy** [^44^](#_ENREF_44) | **Stress** [^45^](#_ENREF_45) | (I) Hypothalamus **Stress** [^46^](#_ENREF_46)  (D) microglia **Stress** [^47^](#_ENREF_47)  (I) AMY **Stress** [^48^](#_ENREF_48)  (D) Cortex **PTSD** [^49^](#_ENREF_49)  (D)  PFC **Psychological Stress** [^41^](#_ENREF_41)  (I) Hippocampus **Stress**  [^50^](#_ENREF_50) | (I) Blood **Chronic Stress** [^51^](#_ENREF_51)  (I)  Blood (Females)  Stress[^8^](#_ENREF_8) | 16 | 1.22E-02/4 Nominal |
| **FKBP5** FK506 Binding Protein 5 | 224840_at | (D) DE/2 41.5% | **PTSD**  [^24^](#_ENREF_24),[^27^](#_ENREF_27),[^28^](#_ENREF_28)  **Childhood Trauma** [^29^](#_ENREF_29),[^30^](#_ENREF_30)  **Psychological Stress**  [^31^](#_ENREF_31),[^32^](#_ENREF_32)  **PTSD**  [^27^](#_ENREF_27),[^33^](#_ENREF_33),[^34^](#_ENREF_34),[^35^](#_ENREF_35),[^36^](#_ENREF_36) | (D) PFC **PTSD** [^37^](#_ENREF_37) | (D)  PBMCs **Post-Deployment PTSD**  [^29^](#_ENREF_29)  (I)  whole blood RNA **PTSD**  [^38^](#_ENREF_38)  DE Blood **PTSD**  [^27^](#_ENREF_27)  (I) PBMC **PTSD**  [^39^](#_ENREF_39)  (D) Whole Blood **PTSD**  [^40^](#_ENREF_40)  (D) Venous Blood **PTSD**  [^14^](#_ENREF_14)  (I) Fasting Blood **PTSD**  [^40^](#_ENREF_40)  (D) PBMC **Psychological Stress**[^41^](#_ENREF_41)  (D) **Relaxation Response** [^42^](#_ENREF_42)  (D) leukocytes **Social Isolation** [^43^](#_ENREF_43)  (I) Blood **Psychotherapy** [^44^](#_ENREF_44) | **Stress** [^45^](#_ENREF_45) | (I) Hypothalamus **Stress** [^46^](#_ENREF_46)  (D) microglia **Stress** [^47^](#_ENREF_47)  (I) AMY **Stress** [^48^](#_ENREF_48)  (D) Cortex **PTSD** [^49^](#_ENREF_49)  (D)  PFC **Psychological Stress** [^41^](#_ENREF_41)  (I) Hippocampus **Stress**  [^50^](#_ENREF_50) | (I) Blood **Chronic Stress** [^51^](#_ENREF_51) | 14 | Not Stepwise |
| **OAS1** 2'-5'-Oligoadenylate Synthetase 1 | 202869_at | (D) DE/4 56.9% |  | (I) DLPFC (BA 46) **PTSD** 18690294 | (I) Blood **Combat-traumas** [^12^](#_ENREF_12)  (D) Blood **PTSD**  [^22^](#_ENREF_22)  (I) Peripheral blood leukocytes **PTSD** [^57^](#_ENREF_57)  (I) PBMC **PTSD**  [^58^](#_ENREF_58)  (D) Leukocytes **Social Isolation** [^43^](#_ENREF_43) |  | (I) NAC **Chronic Stress** [^59^](#_ENREF_59)  (D) NAC **Chronic Stress** [^59^](#_ENREF_59) | (D) Blood **PTSD** [^56^](#_ENREF_56)  (D) Lymphocytes (females) DBP **KO-Stressed** mice, Omega-3 fatty acids [^11^](#_ENREF_11)  (I) Blood (Males) Stress[^8^](#_ENREF_8) | 13 | 1.15E-01/2 Stepwise |
| **SNCA** Synuclein Alpha | 215811_at | (D) AP/2 37.5% | PTSD and alcohol use[^61^](#_ENREF_61) | (I) NAC **Social Isolation** [^10^](#_ENREF_10) | (D)  Blood **Post-Deployment** **PTSD** [^57^](#_ENREF_57)  (D) Blood **Combat-traumas** [^12^](#_ENREF_12)  (D)  Blood **Female specific interpersonal-traumas** [^12^](#_ENREF_12)  (I) Blood **Combat-traumas** [^57^](#_ENREF_57) |  | (D) AMY (males) **Stress** [^7^](#_ENREF_7)  (I) Hippocampus **Stress** [^62^](#_ENREF_62) | (D) Lymphocytes (males) **Stress** [^7^](#_ENREF_7)  (I) Lymphocytes (males) DBP **KO-Stressed** mice, Omega-3 fatty acids [^11^](#_ENREF_11)  (D)  Blood (Males)  Stress[^8^](#_ENREF_8)  (I)  Blood (Females)  Stress[^8^](#_ENREF_8) | 13 | Not Stepwise |
| **RTN4**  Reticulon 4 | 1556049_at | (I) DE/4 54.4% |  | (I) NAC **Social Isolation** [^10^](#_ENREF_10) | PBMCs (I) ( PTSD, ER Trauma survivors) [^39^](#_ENREF_39)  monocytes (I) ( **PTSD**, Assault Trauma) [^13^](#_ENREF_13)  Blood (I) ( **PTSD**, Childhood Trauma) [^22^](#_ENREF_22) |  | (I) Female  PFC, **Chronic Variable Stress**  [^26^](#_ENREF_26) | (I) Lymphocytes (females) **DBP KO-Stressed mice, Omega-3 fatty acids** [^11^](#_ENREF_11) | 13 | Not Stepwise |
| **SUMO1**  Small Ubiquitin-Like Modifier 1 | 208762_at | (D) DE/4 56.3% |  | (I) NAC **Social Isolation** [^10^](#_ENREF_10) | (D PBMC **PTSD** [^39^](#_ENREF_39) |  | (D)  AMY **Chronic Stress** [^63^](#_ENREF_63) | (D)  Blood (Males)  **Stress**[^8^](#_ENREF_8) | 13 | Not Stepwise |
| **NUB1** Negative Regulator Of Ubiquitin Like Proteins 1 | 1560108_at | (I) DE/4 61.8% |  | (I) NAC **Social Isolation** [^10^](#_ENREF_10) | (I) Blood **Combat-traumas** [^12^](#_ENREF_12) |  | (I) Female PFC **Chronic Variable Stress** [^26^](#_ENREF_26) |  | 12 | 2.34E-02/4 Nominal |
| **B2M** Beta-2-Microglobulin | 232311_at | (I) DE/6 91.2% |  | (I) NAC **Social Isolation** [^10^](#_ENREF_10) |  |  | (D)  NAC (female) DBP **KO**-**Stressed** mice, Omega-3 fatty acids [^11^](#_ENREF_11) |  | 11 | Not Stepwise |
| **LAIR1**  Leukocyte Associated Immunoglobulin Like Receptor 1 | 210644_s_at | (D) DE/6 86.2% |  |  | (D)  Blood **PTSD Female specific interpersonal-traumas** [^12^](#_ENREF_12)  (D)  Blood **PTSD Childhood Traum**) [^22^](#_ENREF_22) |  | (I) Ventral Striatum **PTSD**  [^9^](#_ENREF_9) |  | 10.010 | 1.12E-02/4 Nominal |
| **DTNBP1** Dystrobrevin Binding Protein 1 | 223446_s_at | (D) DE/6 93.8% | **PTSD**  genetic database  [^24^](#_ENREF_24) |  | (D) Blood **PTSD**  [^22^](#_ENREF_22)  (I)  PBMC **PTSD**  [^25^](#_ENREF_25) |  |  |  | 10 | Not Stepwise |
| **OXA1L** OXA1L, Mitochondrial Inner Membrane Protein | 208717_at | (D) DE/4 56.9% |  | (D) DLPFC (BA4) **PTSD** [^23^](#_ENREF_23) |  |  | (D) Female NAC **Chronic Variable Stress** [^26^](#_ENREF_26) |  | 10 | 6.40E-03/4 Nominal |
| **DDX6** DEAD-Box Helicase 6 | 1562836_at | (I) DE/6 83.8% (I) AP/6 90.2% |  |  |  |  | (D) PFC (males) **Stress**  [^7^](#_ENREF_7)  (I) AMY (males) **Stress** [^7^](#_ENREF_7) | (I) Blood (Males) Stress[^8^](#_ENREF_8) | 9 | Not Stepwise |
| **CCL4** C-C Motif Chemokine Ligand 4 | 204103_at | (D) DE/6 96.9% |  |  | (I) Venous Blood **PTSD**  [^14^](#_ENREF_14)  (I) Plasma **PTSD**  [^15^](#_ENREF_15)  (D) Peripheral blood monocytes **Chronic Stress** [^16^](#_ENREF_16) |  |  |  | 8 | Not Stepwise |
| **CIRBP** Cold Inducible RNA Binding Protein | 200811_at | (D) DE/4 69.2% |  |  | (D) Blood Female **specific interpersonal-traumas**  [^12^](#_ENREF_12)  (D) Monocytes **Combat-traumas** [^13^](#_ENREF_13) |  | (D) Hippocampus **Stress** [^17^](#_ENREF_17)  (D) Hippocampus **Physical and Cognitive stimulation** [^18^](#_ENREF_18) |  | 8 | 3.66E-02/4 Nominal |
| **CYP2E1** Cytochrome P450 Family 2 Subfamily E Member 1 | 209976_s_at | (I) DE/2 44.1% | **Psychological Stress** [^19^](#_ENREF_19) |  | (I) PBMC **Relaxation Response** [^20^](#_ENREF_20) |  | (D) NAC **Chronic Stress** [^21^](#_ENREF_21) |  | 8 | 1.57E-02/4 Nominal |
| **DCTN5** Dynactin Subunit 5 | 209231_s_at | (D) DE/6 90.8% |  |  | (I) Blood **Childhood Trauma** [^22^](#_ENREF_22) |  |  |  | 8 | Not Stepwise |
| **GJB2** Gap Junction Protein Beta 2 | 223278_at | (I) DE/2 48.5% |  | (I) NAC **Social Isolation** [^10^](#_ENREF_10) |  |  | (I)  **PTSD** [^9^](#_ENREF_9) |  | 8 | 2.42E-02/4 Nominal |
| **HIF1A** Hypoxia Inducible Factor 1 Alpha Subunit | 238869_at | (I) DE/4 54.4% |  |  | (I) Peripheral Blood cells **Psychological Stress** [^52^](#_ENREF_52)  (I) Leukocyte **Stress** [^53^](#_ENREF_53) |  | (I) Hippocampus **Early Life** **Stress** [^54^](#_ENREF_54) |  | 8 | 1.11E-02/4 Nominal |
| **N4BP2L2**  NEDD4 Binding Protein 2 Like 2 | 214388_at | (I) DE/4 69.1% |  |  | (D)  Blood **PTSD Male specific interpersonal-traumas** [^12^](#_ENREF_12)  (D)  Blood **PTSD Childhood Traum)** [^22^](#_ENREF_22) |  | (I) Male  NAC **Chronic Variable Stress** [^26^](#_ENREF_26)  (I)  ST **PTSD** [^9^](#_ENREF_9)  (D) Hippocampus, AMY, Medial PFC, hemibrain **PTSD** [^56^](#_ENREF_56) |  | 8 | 4.40E-02/4 Nominal |
| **NKTR** Natural Killer Cell Triggering Receptor | 243055_at | (I) DE/4 50% (I) AP/2 43.1% |  |  | (I) leukocytes **Social Isolation** [^43^](#_ENREF_43) |  | (I) Male  NAC **Chronic Variable Stress** [^26^](#_ENREF_26)  (D) hippocampus **Stress** [^17^](#_ENREF_17)  (I) Hippocampus (males) DBP **KO-Stressed** mice, Omega-3 fatty acids [^11^](#_ENREF_11)  (I)  PFC (female) DBP **KO-Stressed** mice, Omega-3 fatty acids [^11^](#_ENREF_11) |  | 8 | 1.24E-02/4 Nominal |
| **PCDHB6** Protocadherin Beta 6 | 239443_at | (I) DE/2 38.2% |  | (I) NAC **Social Isolation** [^10^](#_ENREF_10) |  |  | (I) Hippocampus **Stress**  [^60^](#_ENREF_60) |  | 8 | 1.17E-02/4 Nominal |
| **PSD3** Pleckstrin And Sec7 Domain Containing 3 | 218613_at | (D) AP/6 100% |  |  |  |  | (D) Ventral Striatum **PTSD** [^9^](#_ENREF_9)  (I) Hippocampus, Amygdala, Medial PFC, hemibrain **PTSD** [^56^](#_ENREF_56)  (D) AMY (males) **Stress** [^7^](#_ENREF_7) |  | 8 | Not Stepwise |
| **SPON2** Spondin 2 | 218638_s_at | (D) DE/6 93.8% |  |  | (D) Venous Blood **PTSD** [^14^](#_ENREF_14) |  |  |  | 8 | Not Stepwise |
| **UBE2E2** Ubiquitin Conjugating Enzyme E2 E2 | 225651_at | (D) DE/4 53.8% | **PTSD** [^57^](#_ENREF_57) |  | (D) Blood **Post-Deployment PTSD** [^57^](#_ENREF_57) |  |  |  | 8 | 4.41E-02/4 Nominal |
| **HLA-B**  Major Histocompatibility Complex, Class I, B | 211911_x_at | (D) DE/4  52.3% |  |  | (D) PBMC  Stress[^20^](#_ENREF_20) |  |  | (I) Blood (Females) Stress[^8^](#_ENREF_8) | 7 | 4.85E-02/4 Nominal |
| **LCP2** Lymphocyte Cytosolic Protein 2 | 244251_at | (D) DE/4  53.8% |  |  |  |  | (D) Female  NAC **Chronic Variable Stress**  [^26^](#_ENREF_26) | (D) Blood (Males) Stress[^8^](#_ENREF_8) | 7 | 2.01E-02/4 Nominal |
| **PCBP2**  Poly(RC) Binding Protein 2 | 237374_at | (I) DE/2 35.3% |  |  | (I) Blood **PTSD** [^14^](#_ENREF_14) |  | (I)  AMY (Males)  **Stress**[^8^](#_ENREF_8) | Lymphocytes (males) (I) (Treatments, DBP KO-**Stressed mice**, Omega-3 fatty acids) [^11^](#_ENREF_11) | 6.5 | 2.83E-02/4 Nominal |
| **STX11** Syntaxin 11 | 210190_at | (D) DE/2 49.2% |  |  | (I) PBMCs **PTSD ER Trauma survivors)** [^39^](#_ENREF_39)  (D)  Blood **Interpersonal traumas** [^22^](#_ENREF_22) |  | (D) Hippocampus **PTSD** [^9^](#_ENREF_9)  (I) MPFC **PTSD** [^9^](#_ENREF_9)  (I) Hippocampus, Amygdala, Medial PFC, hemibrain **PTSD** [^56^](#_ENREF_56) | (D) Lymphocytes (females) DBP **KO-Stressed** mice, Omega-3 fatty acids [^11^](#_ENREF_11) | 6.5 | 2.74E-02/4 Nominal |
| **ANK2** Ankyrin 2 | 202921_s_at | (I) DE/4 52.9% |  |  |  |  | (I) PFC (males) **Stress** [^7^](#_ENREF_7)  (D)  AMY(Females)  **Stress**[^8^](#_ENREF_8) |  | 6 | 1.09E-02/4 Nominal |
| **APOL3** Apolipoprotein L3 | 221087_s_at | (D) AP/4 50% |  |  |  |  | (D)  **PTSD** [^9^](#_ENREF_9)  (I) MPFC **PTSD** [^9^](#_ENREF_9) |  | 6 | 2.96E-02/4 Nominal |
| **C1orf123** Chromosome 1 Open Reading Frame 123 | 203197_s_at | (D) DE/4 72.3% |  |  | (D) Blood Male specific **interpersonal-traumas** [^12^](#_ENREF_12)  (D) monocytes **Assault Trauma** [^13^](#_ENREF_13) |  |  |  | 6 | 2.92E-02/4 Nominal |
| **DMGDH** Dimethylglycine Dehydrogenase | 231591_at | (I) DE/2 45.6% |  | (D)  DLPFC (BA 46) **PTSD** [^23^](#_ENREF_23) |  |  |  |  | 6 | 3.36E-02/4 Nominal |
| **ELMO2** Engulfment And Cell Motility 2 | 220363_s_at | (D) DE/4  60.0%  (D) AP/4  54.7% |  |  |  |  | (D) Female  PFC, **Chronic Variable Stress**  [^26^](#_ENREF_26)  (D) Male  PFC, NAC **Chronic Variable Stress**  [^26^](#_ENREF_26) |  | 6 | 1.30E-02/4 Nominal |
| **FOXK2** Forkhead Box K2 | 220696_at | (I) DE/4 58.8% (I) AP/4 72.5% |  |  | (I) PBMC **PTSD** [^39^](#_ENREF_39) |  |  |  | 6 | 1.52E-02/4 Nominal |
| **HLA-DRB1** Major Histocompatibility Complex, Class II, DR Beta 1 | 209312_x_at | (D) DE/2 41.5% |  |  | (D) Whole Blood **PTSD** [^40^](#_ENREF_40)  (I) Fasting Blood **PTSD** [^40^](#_ENREF_40)  (D) PBMC **Relaxation Response** [^20^](#_ENREF_20)  (I) Leukocytes **Social Isolation** [^43^](#_ENREF_43) |  | (D) Hypothalamus (PVN) **Stress** [^55^](#_ENREF_55) |  | 6 | 1.22E-02/4 Nominal |
| **LAIR2** Leukocyte Associated Immunoglobulin Like Receptor 2 | 207509_s_at | (D) DE/6 98.5% |  |  |  |  |  |  | 6 | Not Stepwise |
| **LOC105378349** Uncharacterized LOC105378349 | 241143_at | (D) AP/6 90.6% |  |  |  |  |  |  | 6 | Not Stepwise |
| **LRRC59**  Leucine Rich Repeat Containing 59 | 222231_s_at | (D) DE/4  61.5% |  |  |  |  | (D) Male  NAC **Chronic Variable Stress**  [^26^](#_ENREF_26) |  | 6 | 3.15E-02/4 Nominal |
| **MAD1L1** MAD1 Mitotic Arrest Deficient Like 1 | 204857_at | (D) DE/4 72.3% |  |  | (D) PBMC **Chronic Stress** [^16^](#_ENREF_16) |  |  |  | 6 | 1.47E-02/4 Nominal |
| **MKL2** MKL1/Myocardin Like 2 | 1562497_at | (I) AP/4 60.8% |  |  |  |  | (I) Hippocampus **PTSD** [^9^](#_ENREF_9) |  | 6 | 4.58E-02/4 Nominal |
| **PLEKHA5** Pleckstrin Homology Domain Containing A5 | 239559_at | (I) DE/2 35.3% |  |  | (I) Blood **Combattraumas** [^12^](#_ENREF_12) |  | (I) Male  PFC **Chronic Variable Stress** [^26^](#_ENREF_26)  (D) Hippocampus **PTSD** [^9^](#_ENREF_9) |  | 6 | 3.33E-02/4 Nominal |
| **UQCC1** Ubiquinol-Cytochrome C Reductase Complex Assembly Factor 1 | 217935_s_at | (D) DE/2 38.5% |  | (I) NAC **Social Isolation** [^10^](#_ENREF_10) |  |  |  |  | 6 | 3.33E-02/4 Nominal |

**Table S3. Evidence for involvement in other psychiatric and related disorders for Top Predictive Biomarkers for Stress (from Figure 2) (n=41 genes, 42 probesets).** In the same direction of expression as stress**.** Red- increased in expression (I) in Stress, Blue- decreased in expression (D). DE- differential expression, AP-Absent/Present. MDD- Depression; BP- bipolar; SZ- schizophrenia.

| **Gene Symbol/ Gene Name** | **Probeset** | **Discovery (Change) Method/Score  6pts** | **Prioritization Total CFG Score For Stress** | **Validation Anova p-value  6 pts** | **Prior human genetic evidence for other Disorder 2pts.** | **Prior human Brain expression evidence for other Disorder 4 pts** | **Prior human peripheral evidence for other Disorder**  **2pts.** | **Prior Non-human genetic evidence for other Disorder 1pt.** | **Prior Non-human Brain expression evidence for other Disorder 2pts.** | **Prior Non-human peripheral evidence for other Disorder 1pt** | **External CFG for Other Dx** |
| --- | --- | --- | --- | --- | --- | --- | --- | --- | --- | --- | --- |
| **TL**  Telomere Lenght |  | (D) |  | **0.238473**  **NS** | **Epigenetic Aging rates**  [^64^](#_ENREF_64) |  | (D) Leukocyte  **Chronic Alcohol dependence** [^65^](#_ENREF_65)  (D) **Serum**  **BP, Aging**  [^66^](#_ENREF_66)  (D) Leukocyte **Mania BP**  [^67^](#_ENREF_67)  (D)  Leucocyte **SZ** [**^68^**](#_ENREF_68) |  | (D) Hippocampus **Depression-Like** [^69^](#_ENREF_69) | (D) serum **Depression-Like** [^69^](#_ENREF_69) | 7 |
|  |  |  |  |  |  |  |  |  |  |  |  |
| **FKBP5** FK506 Binding Protein 5 | 224856_at | (D) DE/4 53.8% | 16 | 1.22E-02/4 Nominal | **Psychosis** [^70^](#_ENREF_70)  **BP** [^71^](#_ENREF_71)  **Depression**  [^72^](#_ENREF_72), [^73^](#_ENREF_73)  **MDD** [^32^](#_ENREF_32),[^74^](#_ENREF_74),[^75^](#_ENREF_75)   **Unipolar Depression** [^76^](#_ENREF_76)  **MSK Pain** [^77^](#_ENREF_77)  **Pain**  [^78^](#_ENREF_78)  **Suicide, BP** [^71^](#_ENREF_71)  **Suicide**  [^79^](#_ENREF_79),[^80^](#_ENREF_80),[^81^](#_ENREF_81),[^82^](#_ENREF_82)^,^[^83^](#_ENREF_83) | (D) ACC **MDD** [^84^](#_ENREF_84)  (D) AMY **Suicide** [^85^](#_ENREF_85) | (D) Blood **Alcohol** [^86^](#_ENREF_86)  (D) Blood **Female Sucide** [^87^](#_ENREF_87)  (D) Blood **Male Suicide** [^88^](#_ENREF_88) | **Anxiety** [^89^](#_ENREF_89) | (D) NAC **Alcohol** [^90^](#_ENREF_90) |  | 11 |
| **FKBP5** FK506 Binding Protein 5 | 224840_at | (D) DE/2 41.5% | 14 | Not Stepwise | **Psychosis** [^70^](#_ENREF_70)  **BP** [^71^](#_ENREF_71)  **Depression**  [^72^](#_ENREF_72), [^73^](#_ENREF_73)  **MDD** [^32^](#_ENREF_32),[^74^](#_ENREF_74),[^75^](#_ENREF_75)   **Unipolar Depression** [^76^](#_ENREF_76)  **MSK Pain** [^77^](#_ENREF_77)  **Pain**  [^78^](#_ENREF_78)  **Suicide, BP** [^71^](#_ENREF_71)  **Suicide**  [^79^](#_ENREF_79),[^80^](#_ENREF_80),[^81^](#_ENREF_81),[^82^](#_ENREF_82)^,^[^83^](#_ENREF_83) | (D) ACC **MDD** [^84^](#_ENREF_84)  (D) AMY **Suicide** [^85^](#_ENREF_85) | (D) Blood **Alcohol** [^86^](#_ENREF_86)  (D) Blood **Female Sucide** [^87^](#_ENREF_87)  (D) Blood **Male Suicide** [^88^](#_ENREF_88) | **Anxiety** [^89^](#_ENREF_89) | (D) NAC **Alcohol** [^90^](#_ENREF_90) |  | 11 |
| **PSD3** Pleckstrin And Sec7 Domain Containing 3 | 218613_at | (D) AP/6 100% | 8 | Not Stepwise | **Autism** [^91^](#_ENREF_91)  **Alcohol** [^92^](#_ENREF_92)  **Methamphetamine** [^93^](#_ENREF_93)  **ASD** [^94^](#_ENREF_94)  **SZ**  [^95^](#_ENREF_95),[^96^](#_ENREF_96)  **MDD** [^97^](#_ENREF_97)  **Chronic Fatigue Syndrome** [^98^](#_ENREF_98) | (D) Cerebellum **MDD**  [^84^](#_ENREF_84)  (D) prefrontal cortical parvalbumin neurons cells (PV cells) **SZ** [^99^](#_ENREF_99) | (D) Blood **Male Suicide** [^88^](#_ENREF_88)  (D) Blood **Male-BP Suicide** [^100^](#_ENREF_100)  (D) Blood **Suicide** [^100^](#_ENREF_100) | **Alcohol** [^101^](#_ENREF_101) | (D) Ventral tegmental area **Alcohol** [^102^](#_ENREF_102)  (D) AMY (males) **BP** [^7^](#_ENREF_7) |  | 11 |
| **SNCA** Synuclein Alpha | 215811_at | (D) AP/2 37.5% | 13 | Not Stepwise | **Alcohol** [^103^](#_ENREF_103),[^104^](#_ENREF_104), [^61^](#_ENREF_61)  **Methamphetamine** [^105^](#_ENREF_105)  **Aggression** [^61^](#_ENREF_61)  **Parkinson** [^106^](#_ENREF_106) | (D) AMY **SZ** [^107^](#_ENREF_107)  (D) AMY ACC **MDD** [^84^](#_ENREF_84)  (D) Brain **BP** [^108^](#_ENREF_108)  (D) frontal cortex **Alzheimer's Disease** [^109^](#_ENREF_109)  (D) Frontal, motor cortex **Alcohol** [^110^](#_ENREF_110)  (D) superior frontal cortex **Alcohol** [^111^](#_ENREF_111)  (D) DLPFC **MDD** [^112^](#_ENREF_112) | (D) Blood **SZ** [^113^](#_ENREF_113), [^114^](#_ENREF_114)  (D) Blood **Male-BP Suicide** [^100^](#_ENREF_100) |  | (D) AMY,NAC,FC,CP,Hippocampus **Alcohol** [^115^](#_ENREF_115)  (D) Caudate Putamen **Alcohol** [^115^](#_ENREF_115)  (D) Frontal Cortex **Alcohol** [^115^](#_ENREF_115)  (D) ventral tegmental area **BP**  [^116^](#_ENREF_116)  (D) AMY (males) **BP** [^7^](#_ENREF_7) | (D) Blood **Methamphetamine** [^117^](#_ENREF_117)  (D) Lymphocytes (males) **BP**  [^7^](#_ENREF_7) | 11 |
| **PCBP2**  Poly(RC) Binding Protein 2 | 237374_at | (I) DE/2 35.3% | 4.5 | 2.83E-02/4 Nominal |  | (I) Brain **BP** [^108^](#_ENREF_108) | (I) Blood **Female Sucide** [^87^](#_ENREF_87)    (I) Blood **Male Suicide** [^88^](#_ENREF_88)  (I) Blood **Male-BP Suicide** [^100^](#_ENREF_100)  (I) Blood **Suicide** [^100^](#_ENREF_100) |  |  |  |  |
| **RTN4**  Reticulon 4 | 1556049_at | (I) DE/4 54.4% | 9 | Not Stepwise |  | (I)  PFC  BP[^118^](#_ENREF_118) | (I) Blood **Male Suicide** [^88^](#_ENREF_88)  (I) Blood **Male-BP Suicide** [^100^](#_ENREF_100)  (I) Vertebral Disc **Pain** [^119^](#_ENREF_119) |  | (I)  AMY  Alcohol[^120^](#_ENREF_120)  (I)  AMY **PCP**[^121^](#_ENREF_121)  (I) AMY (males) **BP** [^7^](#_ENREF_7) | (I)  Blood **PCP**[^121^](#_ENREF_121) |  |
| **SUMO1**  Small Ubiquitin-Like Modifier 1 | 208762_at | (D) DE/4 56.3% | 9 | Not Stepwise | Longevity[^122^](#_ENREF_122) | (D)  Brain  BP[^108^](#_ENREF_108)    (D)  Thalamus  SZ  [^123^](#_ENREF_123) |  |  |  |  | 6 |
| **ANK2** Ankyrin 2 | 202921_s_at | (I) DE/4 52.9% | 6 | 1.09E-02/4 Nominal | **Alcohol** [^124^](#_ENREF_124)  **ASD** [^125^](#_ENREF_125)  **Autism** [^91^](#_ENREF_91) **Longevity**  [^122^](#_ENREF_122) **Chronic Fatigue Syndrome** [^98^](#_ENREF_98) | (I) Brain **BP**  [^108^](#_ENREF_108)  (I) PFC (BA46) **SZ** [^126^](#_ENREF_126) | (I) Fibroblast **MDD** [^127^](#_ENREF_127)  (I) Blood **Female Sucide** [^87^](#_ENREF_87)  (I) Blood **Suicide** [^100^](#_ENREF_100) |  | (I) PFC (males) **BP** [^7^](#_ENREF_7) |  | 10 |
| **DDX6** DEAD-Box Helicase 6 | 1562836_at | (I) DE/6 83.8%  (I) AP/6 90.2% | 8 | Not Stepwise | **Alcohol** [^128^](#_ENREF_128)  **Other Substances/Addictions** [^128^](#_ENREF_128) | (I) DLPFC **SZ** [^129^](#_ENREF_129) | (I) Blood **Male Suicide** [^88^](#_ENREF_88)  (I) Blood **Male-BP Suicide** [^100^](#_ENREF_100)  (I) Blood **Suicide** [^100^](#_ENREF_100) |  | (I) AMY **MDD** [^63^](#_ENREF_63)  (I) AMY (males) **BP** [^7^](#_ENREF_7)  (I) AMY **Yohimbine**  [^130^](#_ENREF_130) |  | 10 |
| **HIF1A** Hypoxia Inducible Factor 1 Alpha Subunit | 238869_at | (I) DE/4 54.4% | 8 | 1.11E-02/4 Nominal | **SZ** [^131^](#_ENREF_131) | (I) Frontal and temporal cortex **Autism**  [^132^](#_ENREF_132)  (I) cerebral cortex **Autism,SZ** [^91^](#_ENREF_91) | (I) Blood **Longevity**[^133^](#_ENREF_133)  (I) peripheral white blood cells BP,**MDD** [^134^](#_ENREF_134)  (I) Blood **Huntington's Disease** [^135^](#_ENREF_135)  (I) Vertebral Disc **Pain** [^119^](#_ENREF_119) |  | (I) AMY **Alcohol** [^136^](#_ENREF_136) |  | 10 |
| **DTNBP1** Dystrobrevin Binding Protein 1 | 223446_s_at | (D) DE/6 93.8% | 10 | Not Stepwise | **Autism** [^91^](#_ENREF_91)  **Methamphetamine** [^137^](#_ENREF_137)  **Psychosis**  [^138^](#_ENREF_138),[^139^](#_ENREF_139),[^140^](#_ENREF_140)  **SZ**  [^141^](#_ENREF_141),[^142^](#_ENREF_142),[^143^](#_ENREF_143),[^144^](#_ENREF_144),[^145^](#_ENREF_145)  **BP**  [^146^](#_ENREF_146),[^142^](#_ENREF_142),[^147^](#_ENREF_147),[^148^](#_ENREF_148),[^149^](#_ENREF_149),[^150^](#_ENREF_150)**,**[^151^](#_ENREF_151) | (D) Brain **BP** [^108^](#_ENREF_108)  (D) DLPFC **SZ** [^152^](#_ENREF_152)  (D) Hippocampus (CA3) **SZ** [^153^](#_ENREF_153)  (D) cerebral cortex **Autism** [^91^](#_ENREF_91)  (D) Hippocampus,pSTG **SZ** [^154^](#_ENREF_154) | (D) lymphocytes **SZ** [^155^](#_ENREF_155)  (D) Blood **Suicide** [^100^](#_ENREF_100) | **Intellect** [^156^](#_ENREF_156)    **SZ**  [^157^](#_ENREF_157),[^158^](#_ENREF_158),[^159^](#_ENREF_159),[^160^](#_ENREF_160),[^161^](#_ENREF_161),[^162^](#_ENREF_162),[^163^](#_ENREF_163),[^164^](#_ENREF_164),[^165^](#_ENREF_165) |  |  | 9 |
| **APOL3** Apolipoprotein L3 | 221087_s_at | (D) AP/4 50% | 6 | 2.96E-02/4 Nominal | **ADHD**  [^166^](#_ENREF_166) | (D) cerebral cortex **SZ** [^91^](#_ENREF_91) | (D) Blood **Female Sucide** [^87^](#_ENREF_87)  (D) Blood **Male Suicide** [^88^](#_ENREF_88) |  |  |  | 8 |
| **B2M** Beta-2-Microglobulin | 232311_at | (I) DE/6 91.2% | 11 | Not Stepwise |  | (I) cerebral cortex **Autism** [^91^](#_ENREF_91) | (I) Blood Male Suicide [^88^](#_ENREF_88)  Blood **Male-BP Suicide** [^100^](#_ENREF_100)  (I) Blood **Suicide**  [^100^](#_ENREF_100)  (I) Blood **Atypical depression** [^167^](#_ENREF_167)  (I) CSF **MDD** [^168^](#_ENREF_168)  (I) CSF **Pain** [^169^](#_ENREF_169) |  | (I) Hippocampus **Aging** [^170^](#_ENREF_170)  (I) Hypothalamus **Eating Disorder**[**^171^**](#_ENREF_171)  (I) ventral tegmental area **Alcohol** 23714385 |  | 8 |
| **DCTN5** Dynactin Subunit 5 | 209231_s_at | (D) DE/6 90.8% | 8 | Not Stepwise | **BP**  [^150^](#_ENREF_150), cnv  [^172^](#_ENREF_172) | (D) Brain **BP** [^108^](#_ENREF_108) | (D) Blood **Male Suicide** [^88^](#_ENREF_88)  (D) Blood **Suicide** [^100^](#_ENREF_100) |  |  |  | 8 |
| **HLA-DRB1** Major Histocompatibility Complex, Class II, DR Beta 1 | 209312_x_at | (D) DE/2 41.5% | 6 | 1.22E-02/4 Nominal | **Longevity**[^122^](#_ENREF_122)  [^173^](#_ENREF_173),[^174^](#_ENREF_174), [^175^](#_ENREF_175)   **Alzheimer's Disease** [^176^](#_ENREF_176)  **SZ**  [^177^](#_ENREF_177)  **Pain** [^78^](#_ENREF_78)  **Panic Disorder**[^178^](#_ENREF_178) | (D) Brain **BP** [^108^](#_ENREF_108)  (D) DLPFC **SZ** [^179^](#_ENREF_179)  (D) frontal **Alcohol** [^180^](#_ENREF_180)  (D) PFC **Alcohol** [^181^](#_ENREF_181) |  |  | (D) NAC **Alcohol** [^182^](#_ENREF_182) |  | 8 |
| **MAD1L1** MAD1 Mitotic Arrest Deficient Like 1 | 204857_at | (D) DE/4 72.3% | 6 | 1.47E-02/4 Nominal | **Autism** [^91^](#_ENREF_91)  **SZ**  [^177^](#_ENREF_177),[^183^](#_ENREF_183),[^184^](#_ENREF_184),[^185^](#_ENREF_185),[^186^](#_ENREF_186),[^187^](#_ENREF_187), [^188^](#_ENREF_188)  **BP**[^189^](#_ENREF_189), [^190^](#_ENREF_190),[^191^](#_ENREF_191),[^186^](#_ENREF_186)^,^[^192^](#_ENREF_192), [^193^](#_ENREF_193) [^194^](#_ENREF_194),[^195^](#_ENREF_195) | (D) cerebral cortex **Autism** [^91^](#_ENREF_91) | (D) SH-SY5Y cells (D) **Cocaine**  [^196^](#_ENREF_196)  Blood **BP** [^197^](#_ENREF_197) |  |  |  | 8 |
| **N4BP2L2**  NEDD4 Binding Protein 2 Like 2 | 214388_at | (I) DE/4 69.1% | 8 | 4.40E-02/4 Nominal | **SZ**  [^91^](#_ENREF_91),[^131^](#_ENREF_131) | (I) DLPFC **SZ** [^179^](#_ENREF_179)  (I) Dorsal Lateral PFC **MDD** [^198^](#_ENREF_198)  (I) NAC **Suicide** [^199^](#_ENREF_199)  (I) cerebral cortex **SZ** [^91^](#_ENREF_91) | (I) L neurons **BP**  [^200^](#_ENREF_200)  (I) Blood **Female Sucide** [^87^](#_ENREF_87)  (I) Blood **Male Suicide** [^88^](#_ENREF_88)  (I) Blood **Male-BP Suicide** [^100^](#_ENREF_100)  (I) Blood **Suicide**  [^100^](#_ENREF_100) |  |  |  | 8 |
| **OAS1** 2'-5'-Oligoadenylate Synthetase 1 | 202869_at | (D) DE/4 56.9% | 13 | 1.15E-01/2 Stepwise |  | (D) Hippocampus **Alcohol** [^201^](#_ENREF_201) | (D) Peripheral Blood cells **Panic Disorder** [^202^](#_ENREF_202)  (D) Blood mononuclear cell (BMC) **Alzheimer's Disease** [^203^](#_ENREF_203)  (D) **Depression-Related** [^204^](#_ENREF_204) |  | (D) PFC **MDD** [^205^](#_ENREF_205) |  | 8 |
| **OXA1L** OXA1L, Mitochondrial Inner Membrane Protein | 208717_at | (D) DE/4 56.9% | 10 | 6.40E-03/4 Nominal | **Autism** [^91^](#_ENREF_91) | (D) Frontal and temporal cortex **Autism** [^132^](#_ENREF_132)  (D) PFC (BA46) **BP,SZ** [^126^](#_ENREF_126)  (D) Cerebral Cortex **Autism** [^91^](#_ENREF_91) | (D) Blood **Male Suicide** [^88^](#_ENREF_88)  (D) Blood **Suicide**  [^100^](#_ENREF_100) |  |  |  | 8 |
| **SPON2** Spondin 2 | 218638_s_at | (D) DE/6 93.8% | 8 | Not Stepwise | **Autism** [^91^](#_ENREF_91) | (D) Cerebral Cortex **Autism,BP,SZ** [^91^](#_ENREF_91) | (D) Lymphocyte **Panic Disorder** [^206^](#_ENREF_206)  (D) PBMC cells **BP** [^207^](#_ENREF_207) |  |  |  | 8 |
| **CCL4** C-C Motif Chemokine Ligand 4 | 204103_at | (D) DE/6 96.9% | 8 | Not Stepwise |  | (D) Hippocampus **SZ** [^208^](#_ENREF_208)  (D) PFC (BA9) **MDD** [^209^](#_ENREF_209) | (D) Blood **Depression** [^210^](#_ENREF_210)  (D) CSF, plasma **Suicide** [^211^](#_ENREF_211) |  |  | (D) Plasma **Alcohol** [^212^](#_ENREF_212) | 7 |
| **NKTR** Natural Killer Cell Triggering Receptor | 243055_at | (I) DE/4 50% (I) AP/2 43.1% | 8 | 1.24E-02/4 Nominal |  | (I) AMY and cingulate cortex **MDD** [^213^](#_ENREF_213)  (I) DLPFC **SZ**  [^179^](#_ENREF_179) | (I) L neurons **BP** [^200^](#_ENREF_200)  (I) Blood **Female Sucide** [^87^](#_ENREF_87)  (I) Blood **Male-BP Suicide** [^100^](#_ENREF_100) | Alcohol [^214^](#_ENREF_214) |  |  | 7 |
| **CIRBP** Cold Inducible RNA Binding Protein | 200811_at | (D) DE/4 69.2% | 8 | 3.66E-02/4 Nominal |  | (D) Cerebral Cortex **Autism** [^91^](#_ENREF_91) | Differentially methylated Whole blood DNA **SZ** [^215^](#_ENREF_215) |  |  |  | 6 |
| **FOXK2** Forkhead Box K2 | 220696_at | (I) DE/4 58.8% (I) AP/4 72.5% | 6 | 1.52E-02/4 Nominal | **Autism** [^91^](#_ENREF_91)   **Suicide** [^216^](#_ENREF_216) |  | (I) Blood **Female Sucide** [^87^](#_ENREF_87)  (I) Blood **Male-BP Suicide** [^100^](#_ENREF_100)  (I) Blood **Suicide**  [^100^](#_ENREF_100)  (I) Blood **Delusions, Hallucinations** [^217^](#_ENREF_217) |  | (I) AMY, Hippocampus **Alcohol** [^182^](#_ENREF_182) |  | 6 |
| **UQCC1** Ubiquinol-Cytochrome C Reductase Complex Assembly Factor 1 | 217935_s_at | (D) DE/2 38.5% | 6 | 3.33E-02/4 Nominal |  | (D) Brain **BP** [^108^](#_ENREF_108) | (D) Blood **Female Sucide** [^87^](#_ENREF_87)  (D) Blood **Male-BP Suicide** [^100^](#_ENREF_100)  (D) Blood **Suicide**  [^100^](#_ENREF_100) |  |  |  | 6 |
| **CYP2E1** Cytochrome P450 Family 2 Subfamily E Member 1 | 209976_s_at | (I) DE/2 44.1% | 8 | 1.57E-02/4 Nominal | **Alcohol** [^218^](#_ENREF_218)  **SZ** [^219^](#_ENREF_219) |  | (I) Blood **Suicide**  [^100^](#_ENREF_100) |  |  |  | 4 |
| **LCP2** Lymphocyte Cytosolic Protein 2 | 244251_at | (D) DE/4  53.8% | 6 | 2.01E-02/4 Nominal | **ASD** [**^94^**](#_ENREF_94) |  |  |  | (D) Ventral Hippocampus **MDD** [^205^](#_ENREF_205) |  | 4 |
| **LRRC59** Leucine Rich Repeat Containing 59 | 222231_s_at | (D) DE/4  61.5% | 6 | 3.15E-02/4 Nominal |  | (D) cerebral cortex  **SZ**  [^91^](#_ENREF_91) |  |  |  |  | 4 |
| **NUB1** Negative Regulator Of Ubiquitin Like Proteins 1 | 1560108_at | (I) DE/4 61.8% | 12 | 2.34E-02/4 Nominal | **Autism** [^91^](#_ENREF_91) |  | (I) Blood **Female Sucide** [^87^](#_ENREF_87)  (I) Blood **Male Suicide** [^88^](#_ENREF_88),[^220^](#_ENREF_220)  (I) Blood **Male-BP Suicide** [^100^](#_ENREF_100)  (I) Blood **Suicide** [^100^](#_ENREF_100) |  |  |  | 4 |
| **PLEKHA5** Pleckstrin Homology Domain Containing A5 | 239559_at | (I) DE/2 35.3% | 6 | 3.33E-02/4 Nominal | **BP** [^221^](#_ENREF_221) |  | (I) Blood **Male Suicide** [^88^](#_ENREF_88) |  |  |  | 4 |
| **C1orf123** Chromosome 1 Open Reading Frame 123 | 203197_s_at | (D) DE/4 72.3% | 6 | 2.92E-02/4 Nominal |  |  | (D) Blood **Female Suicide** [^87^](#_ENREF_87) |  |  |  | 2 |
| **DMGDH** Dimethylglycine Dehydrogenase | 231591_at | (I) DE/2 45.6% | 6 | 3.36E-02/4 Nominal |  |  | (I) Blood **Suicide**  [^100^](#_ENREF_100)  (I) Blood **Delusions**[^217^](#_ENREF_217) |  |  |  | 2 |
| **ELMO2** Engulfment And Cell Motility 2 | 220363_s_at | (D) DE/4  60.0%  (D) AP/4  54.7% | 6 | 1.30E-02/4 Nominal |  |  | (D) Blood **Suicide**  [^100^](#_ENREF_100) |  |  |  | 2 |
| **GJB2** Gap Junction Protein Beta 2 | 223278_at | (I) DE/2 48.5% | 8 | 2.42E-02/4 Nominal |  |  |  |  | (I) Ventral Hippocampus **MDD** [^205^](#_ENREF_205) |  | 2 |
| **LAIR1**  Leukocyte Associated Immunoglobulin Like Receptor 1 | 210644_s_at | (D) DE/6 86.2% | 10 | 1.12E-02/4 Nominal |  |  | (D) Blood **Suicide** [^100^](#_ENREF_100) |  |  |  | 2 |
| **LAIR2** Leukocyte Associated Immunoglobulin Like Receptor 2 | 207509_s_at | (D) DE/6 98.5% | 6 | Not Stepwise |  |  | (D) Blood **Male Suicide** [^88^](#_ENREF_88) |  |  |  | 2 |
| **MKL2** MKL1/Myocardin Like 2 | 1562497_at | (I) AP/4 60.8% | 6 | 4.58E-02/4 Nominal | **Autism** [^91^](#_ENREF_91)  **SZ** [^222^](#_ENREF_222) |  |  |  |  |  | 2 |
| **PCDHB6** Protocadherin Beta 6 | 239443_at | (I) DE/2 38.2% | 8 | 1.17E-02/4 Nominal |  |  | (I) Blood **Female Sucide** [^87^](#_ENREF_87) |  |  |  | 2 |
| **UBE2E2** Ubiquitin Conjugating Enzyme E2 E2 | 225651_at | (D) DE/4 53.8% | 8 | 4.41E-02/4 Nominal | **Psychosis** [^223^](#_ENREF_223) |  |  |  |  |  | 2 |
| **HLA-B**  Major Histocompatibility Complex, Class I, B | 211911_x_at | (D) DE/4  52.3% | 6 | 4.85E-02/4 Nominal |  |  |  |  |  |  | 0 |
| **LOC105378349** Uncharacterized LOC105378349 | 241143_at | (D) AP/6 90.6% | 6 | Not Stepwise |  |  |  |  |  |  | 0 |
| **STX11** Syntaxin 11 | 210190_at | (D) DE/2 49.2% | 6.5 | 2.74E-02/4 Nominal |  |  |  |  |  |  | 0 |

**Table S4. Pharmacogenomics of Top Predictive Biomarkers for Stress (from Figure 2) (n=41 genes, 42 probesets)** Top predictive biomarkers in our datasets that are targets of existing drugs and are modulated by them in opposite direction.

| **Gene Symbol/ Gene Name** | **Probeset** | **Discovery (Change) Method/Score  6pts** | **Prioritization Total CFG Score For Stress** | **Validation Anova p-value  6 pts** | **Omega-3** | **Antidepressants** | **Mood Stabilizers** | **Antipsychotics** | **Other Treatments** |
| --- | --- | --- | --- | --- | --- | --- | --- | --- | --- |
| **TL**  Telomere Length  Reference marker from literature |  | (D) |  | Not Stepwise | (I)  Peripheral Blood Mononuclearcytes  **Omega-3 fatty acids**  [^224^](#_ENREF_224) | (I)  C. Elegans  **Mianserin**  [^225^](#_ENREF_225) | (I)  Saliva  **Lithium**  [^226^](#_ENREF_226)  (I)  Blood  **Lithium**[**^227^**](#_ENREF_227) | (I)  Peripheral Blood Leukocytes  **Olanzapine**  [^228^](#_ENREF_228) | (I)  Peripheral Blood Leukocytes  **Meditation**  [^229^](#_ENREF_229),[^230^](#_ENREF_230) |
|  |  |  |  |  |  |  |  |  |  |
| **FKBP5** FK506 Binding Protein 5 | 224856_at | (D) DE/4 53.8% | 16 | **1.22E-02/4 Nominal** |  |  | (I) Cerebral Cortex (right) **Lithium** [^231^](#_ENREF_231) |  | (I) Blood **Psychotherapy** [^44^](#_ENREF_44) |
| **FKBP5** FK506 Binding Protein 5 | 224840_at | (D) DE/2 41.5% | 14 | Not Stepwise |  |  | (I) Cerebral Cortex (right) **Lithium**[^231^](#_ENREF_231) |  | (I) Blood **Psychotherapy** [^44^](#_ENREF_44) |
| **RTN4**  Reticulon 4 | 1556049_at | (I) DE/4 54.4% | 13 | Not Stepwise | (D)  Lymphocytes (females)  **Omega-3**[^11^](#_ENREF_11) |  | (D) VT **Valproate**[^232^](#_ENREF_232) |  |  |
| **OAS1** 2'-5'-Oligoadenylate Synthetase 1 | 202869_at | (D) DE/4 56.9% | 13 | 1.15E-01/2 Stepwise |  |  | (I) Blood mononuclear cells **Lithium** [^233^](#_ENREF_233) |  |  |
| **SNCA** Synuclein Alpha | 215811_at | (D) AP/2 37.5% | 13 | Not Stepwise | (I) Lymphocytes (males) DBP KO-Stressed mice**, Omega-3 fatty acids** [^11^](#_ENREF_11) |  | (I) NT2.D1 cells **Lithium** [^234^](#_ENREF_234) |  |  |
| **B2M** Beta-2-Microglobulin | 232311_at | (I) DE/6 91.2% | 11 | Not Stepwise | (D) NAC (females) DBP KO-Stressed mice, **Omega-3 fatty acids** [^11^](#_ENREF_11) |  |  |  | 4'-iodo-4'-deoxydoxorubicin |
| **NUB1** Negative Regulator Of Ubiquitin Like Proteins 1 | 1560108_at | (I) DE/4 61.8% | 12 | **2.34E-02/4 Nominal** |  |  |  | (D) VT **Clozapine** [^121^](#_ENREF_121) |  |
| **GJB2** Gap Junction Protein Beta 2 | 223278_at | (I) DE/2 48.5% | 8 | **2.42E-02/4 Nominal** |  |  |  | (D)  VT **Clozapine** [^121^](#_ENREF_121) |  |
| **HIF1A** Hypoxia Inducible Factor 1 Alpha Subunit | 238869_at | (I) DE/4 54.4% | 8 | **1.11E-02/4 Nominal** |  |  |  |  | EZN 2968 |
| **LRRC59** Leucine Rich Repeat Containing 59 | 222231_s_at | (D) DE/4  61.5% | 6 | **3.15E-02/4 Nominal** |  |  |  | (I) CP **Valproate** [^232^](#_ENREF_232) |  |
| **PSD3** Pleckstrin And Sec7 Domain Containing 3 | 218613_at | (D) AP/6 100% | 8 | Not Stepwise |  |  |  | (I) VT **Clozapine** [^121^](#_ENREF_121) |  |
| **STX11** Syntaxin 11 | 210190_at | (D) DE/2 49.2% | 6.5 | **2.74E-02/4 Nominal** |  | (I) MNC **Antidepressants**[^235^](#_ENREF_235) | (I) Lymphoblastoid cell cultures **Lithium** [^236^](#_ENREF_236)  (I) Lymphoblastoid cell cultures **Valproate** [^236^](#_ENREF_236) |  |  |
| **ANK2** Ankyrin 2 | 202921_s_at | (I) DE/4 52.9% | 6 | **1.09E-02/4 Nominal** |  | (D) C.elegans **Mianserin** [^237^](#_ENREF_237) |  |  |  |
| **HLA-DRB1** Major Histocompatibility Complex, Class II, DR Beta 1 | 209312_x_at | (D) DE/2 41.5% | 6 | **1.22E-02/4 Nominal** |  |  |  |  | apolizumab |
| **LAIR2** Leukocyte Associated Immunoglobulin Like Receptor 2 | 207509_s_at | (D) DE/6 98.5% | 6 | Not Stepwise |  | (I) Blood **Antidepressants**[^238^](#_ENREF_238) |  |  |  |

1. Boks, M.P. *et al.* Longitudinal changes of telomere length and epigenetic age related to traumatic stress and post-traumatic stress disorder. *Psychoneuroendocrinology* **51**, 506-12 (2015).

2. Kim, T.Y. *et al.* The effect of trauma and PTSD on telomere length: An exploratory study in people exposed to combat trauma. *Sci Rep* **7**, 4375 (2017).

3. Bersani, F.S. *et al.* Association of dimensional psychological health measures with telomere length in male war veterans. *J Affect Disord* **190**, 537-542 (2016).

4. Blaze, J. *et al.* Intrauterine exposure to maternal stress alters Bdnf IV DNA methylation and telomere length in the brain of adult rat offspring. *Int J Dev Neurosci* **62**, 56-62 (2017).

5. Cram, D.L., Monaghan, P., Gillespie, R. & Clutton-Brock, T. Effects of early-life competition and maternal nutrition on telomere lengths in wild meerkats. *Proc Biol Sci* **284**(2017).

6. Dong, Y., Zhang, G., Yuan, X., Zhang, Y. & Hu, M. Telomere length and telomere repeating factors: Cellular markers for post-traumatic stress disorder-like model. *J Affect Disord* **195**, 156-62 (2016).

7. Le-Niculescu, H. *et al.* Phenomic, convergent functional genomic, and biomarker studies in a stress-reactive genetic animal model of bipolar disorder and co-morbid alcoholism. *Am J Med Genet B Neuropsychiatr Genet* **147B**, 134-66 (2008).

8. Daskalakis, N.P., Cohen, H., Cai, G., Buxbaum, J.D. & Yehuda, R. Expression profiling associates blood and brain glucocorticoid receptor signaling with trauma-related individual differences in both sexes. *Proc Natl Acad Sci U S A* **111**, 13529-34 (2014).

9. Muhie, S. *et al.* Brain transcriptome profiles in mouse model simulating features of post-traumatic stress disorder. *Mol Brain* **8**, 14 (2015).

10. Canli, T. *et al.* Differential transcriptome expression in human nucleus accumbens as a function of loneliness. *Mol Psychiatry* **22**, 1069-1078 (2017).

11. Le-Niculescu, H. *et al.* Convergent functional genomic studies of omega-3 fatty acids in stress reactivity, bipolar disorder and alcoholism. *Transl Psychiatry* **1**, e4 (2011).

12. Breen, M.S. *et al.* PTSD Blood Transcriptome Mega-Analysis: Shared Inflammatory Pathways across Biological Sex and Modes of Trauma. *Neuropsychopharmacology* **43**, 469-481 (2018).

13. Neylan, T.C. *et al.* Suppressed monocyte gene expression profile in men versus women with PTSD. *Brain Behav Immun* **25**, 524-31 (2011).

14. Guardado, P. *et al.* Altered gene expression of the innate immune, neuroendocrine, and nuclear factor-kappa B (NF-kappaB) systems is associated with posttraumatic stress disorder in military personnel. *J Anxiety Disord* **38**, 9-20 (2016).

15. Dalgard, C. *et al.* The MCP-4/MCP-1 ratio in plasma is a candidate circadian biomarker for chronic post-traumatic stress disorder. *Transl Psychiatry* **7**, e1025 (2017).

16. Miller, G.E. *et al.* A functional genomic fingerprint of chronic stress in humans: blunted glucocorticoid and increased NF-kappaB signaling. *Biol Psychiatry* **64**, 266-72 (2008).

17. Malki, K. *et al.* The endogenous and reactive depression subtypes revisited: integrative animal and human studies implicate multiple distinct molecular mechanisms underlying major depressive disorder. *BMC Med* **12**, 73 (2014).

18. Huttenrauch, M., Salinas, G. & Wirths, O. Effects of Long-Term Environmental Enrichment on Anxiety, Memory, Hippocampal Plasticity and Overall Brain Gene Expression in C57BL6 Mice. *Front Mol Neurosci* **9**, 62 (2016).

19. Kumsta, R. *et al.* Severe psychosocial deprivation in early childhood is associated with increased DNA methylation across a region spanning the transcription start site of CYP2E1. *Transl Psychiatry* **6**, e830 (2016).

20. Bhasin, M.K. *et al.* Relaxation response induces temporal transcriptome changes in energy metabolism, insulin secretion and inflammatory pathways. *PLoS One* **8**, e62817 (2013).

21. Descalzi, G. *et al.* Neuropathic pain promotes adaptive changes in gene expression in brain networks involved in stress and depression. *Sci Signal* **10**(2017).

22. Mehta, D. *et al.* Childhood maltreatment is associated with distinct genomic and epigenetic profiles in posttraumatic stress disorder. *Proc Natl Acad Sci U S A* **110**, 8302-7 (2013).

23. Su, Y.A. *et al.* Dysregulated mitochondrial genes and networks with drug targets in postmortem brain of patients with posttraumatic stress disorder (PTSD) revealed by human mitochondria-focused cDNA microarrays. *Int J Biol Sci* **4**, 223-35 (2008).

24. Zhang, K. *et al.* An overview of posttraumatic stress disorder genetic studies by analyzing and integrating genetic data into genetic database PTSDgene. *Neurosci Biobehav Rev* **83**, 647-656 (2017).

25. Tylee, D.S. *et al.* Blood-based gene-expression biomarkers of post-traumatic stress disorder among deployed marines: A pilot study. *Psychoneuroendocrinology* **51**, 472-94 (2015).

26. Labonte, B. *et al.* Sex-specific transcriptional signatures in human depression. *Nat Med* **23**, 1102-1111 (2017).

27. Mehta, D. *et al.* Using polymorphisms in FKBP5 to define biologically distinct subtypes of posttraumatic stress disorder: evidence from endocrine and gene expression studies. *Arch Gen Psychiatry* **68**, 901-10 (2011).

28. Sarapas, C. *et al.* Genetic markers for PTSD risk and resilience among survivors of the World Trade Center attacks. *Dis Markers* **30**, 101-10 (2011).

29. van Zuiden, M. *et al.* Glucocorticoid receptor pathway components predict posttraumatic stress disorder symptom development: a prospective study. *Biol Psychiatry* **71**, 309-16 (2012).

30. Koenen, K.C. *et al.* Polymorphisms in FKBP5 are associated with peritraumatic dissociation in medically injured children. *Mol Psychiatry* **10**, 1058-9 (2005).

31. Ising, M. *et al.* Polymorphisms in the FKBP5 gene region modulate recovery from psychosocial stress in healthy controls. *Eur J Neurosci* **28**, 389-98 (2008).

32. Velders, F.P. *et al.* Genetics of cortisol secretion and depressive symptoms: a candidate gene and genome wide association approach. *Psychoneuroendocrinology* **36**, 1053-61 (2011).

33. Binder, E.B. *et al.* Association of FKBP5 polymorphisms and childhood abuse with risk of posttraumatic stress disorder symptoms in adults. *JAMA* **299**, 1291-305 (2008).

34. Xie, P. *et al.* Interaction of FKBP5 with childhood adversity on risk for post-traumatic stress disorder. *Neuropsychopharmacology* **35**, 1684-92 (2010).

35. Klengel, T. *et al.* Allele-specific FKBP5 DNA demethylation mediates gene-childhood trauma interactions. *Nat Neurosci* **16**, 33-41 (2013).

36. Dunn, E.C. *et al.* Interaction between genetic variants and exposure to Hurricane Katrina on post-traumatic stress and post-traumatic growth: a prospective analysis of low income adults. *J Affect Disord* **152-154**, 243-9 (2014).

37. Holmes, S.E. *et al.* Altered metabotropic glutamate receptor 5 markers in PTSD: In vivo and postmortem evidence. *Proc Natl Acad Sci U S A* **114**, 8390-8395 (2017).

38. Kuan, P.F. *et al.* Gene expression associated with PTSD in World Trade Center responders: An RNA sequencing study. *Transl Psychiatry* **7**, 1297 (2017).

39. Segman, R.H. *et al.* Peripheral blood mononuclear cell gene expression profiles identify emergent post-traumatic stress disorder among trauma survivors. *Mol Psychiatry* **10**, 500-13, 425 (2005).

40. Yehuda, R. *et al.* Gene expression patterns associated with posttraumatic stress disorder following exposure to the World Trade Center attacks. *Biol Psychiatry* **66**, 708-11 (2009).

41. Schmidt, U. *et al.* A role for synapsin in FKBP51 modulation of stress responsiveness: Convergent evidence from animal and human studies. *Psychoneuroendocrinology* **52**, 43-58 (2015).

42. Kuo, B. *et al.* Genomic and clinical effects associated with a relaxation response mind-body intervention in patients with irritable bowel syndrome and inflammatory bowel disease. *PLoS One* **10**, e0123861 (2015).

43. Cole, S.W. *et al.* Social regulation of gene expression in human leukocytes. *Genome Biol* **8**, R189 (2007).

44. Yehuda, R. *et al.* Epigenetic Biomarkers as Predictors and Correlates of Symptom Improvement Following Psychotherapy in Combat Veterans with PTSD. *Front Psychiatry* **4**, 118 (2013).

45. Hartmann, J. *et al.* The involvement of FK506-binding protein 51 (FKBP5) in the behavioral and neuroendocrine effects of chronic social defeat stress. *Neuropharmacology* **62**, 332-9 (2012).

46. Lee, H.C. *et al.* Gene expression profiling in hypothalamus of immobilization-stressed mouse using cDNA microarray. *Brain Res Mol Brain Res* **135**, 293-300 (2005).

47. Wohleb, E.S. *et al.* beta-Adrenergic receptor antagonism prevents anxiety-like behavior and microglial reactivity induced by repeated social defeat. *J Neurosci* **31**, 6277-88 (2011).

48. Skrzypiec, A.E. *et al.* Stress-induced lipocalin-2 controls dendritic spine formation and neuronal activity in the amygdala. *PLoS One* **8**, e61046 (2013).

49. Toth, M. *et al.* Overexpression of Forebrain CRH During Early Life Increases Trauma Susceptibility in Adulthood. *Neuropsychopharmacology* **41**, 1681-90 (2016).

50. Yang, X. *et al.* Glucocorticoid-induced loss of DNA methylation in non-neuronal cells and potential involvement of DNMT1 in epigenetic regulation of Fkbp5. *Biochem Biophys Res Commun* **420**, 570-5 (2012).

51. Lee, R.S. *et al.* A measure of glucocorticoid load provided by DNA methylation of Fkbp5 in mice. *Psychopharmacology (Berl)* **218**, 303-12 (2011).

52. Morita, K. *et al.* Expression analysis of psychological stress-associated genes in peripheral blood leukocytes. *Neurosci Lett* **381**, 57-62 (2005).

53. Ohmori, T. *et al.* Assessment of human stress and depression by DNA microarray analysis. *J Med Invest* **52 Suppl**, 266-71 (2005).

54. Suri, D., Bhattacharya, A. & Vaidya, V.A. Early stress evokes temporally distinct consequences on the hippocampal transcriptome, anxiety and cognitive behaviour. *Int J Neuropsychopharmacol* **17**, 289-301 (2014).

55. Reyes, T.M., Walker, J.R., DeCino, C., Hogenesch, J.B. & Sawchenko, P.E. Categorically distinct acute stressors elicit dissimilar transcriptional profiles in the paraventricular nucleus of the hypothalamus. *J Neurosci* **23**, 5607-16 (2003).

56. Muhie, S. *et al.* Molecular indicators of stress-induced neuroinflammation in a mouse model simulating features of post-traumatic stress disorder. *Transl Psychiatry* **7**, e1135 (2017).

57. Breen, M.S. *et al.* Gene networks specific for innate immunity define post-traumatic stress disorder. *Mol Psychiatry* **20**, 1538-45 (2015).

58. Glatt, S.J. *et al.* Blood-based gene-expression predictors of PTSD risk and resilience among deployed marines: a pilot study. *Am J Med Genet B Neuropsychiatr Genet* **162B**, 313-26 (2013).

59. Hodes, G.E. *et al.* Sex Differences in Nucleus Accumbens Transcriptome Profiles Associated with Susceptibility versus Resilience to Subchronic Variable Stress. *J Neurosci* **35**, 16362-76 (2015).

60. Jakobsson, J. *et al.* KAP1-mediated epigenetic repression in the forebrain modulates behavioral vulnerability to stress. *Neuron* **60**, 818-31 (2008).

61. Guillot, C.R., Fanning, J.R., Liang, T., Leventhal, A.M. & Berman, M.E. An alpha-synuclein gene (SNCA) polymorphism moderates the association of PTSD symptomatology with hazardous alcohol use, but not with aggression-related measures. *J Anxiety Disord* **30**, 41-7 (2015).

62. Dimatelis, J.J. *et al.* Exercise partly reverses the effect of maternal separation on hippocampal proteins in 6-hydroxydopamine-lesioned rat brain. *Exp Physiol* **98**, 233-44 (2013).

63. Andrus, B.M. *et al.* Gene expression patterns in the hippocampus and amygdala of endogenous depression and chronic stress models. *Mol Psychiatry* **17**, 49-61 (2012).

64. Lu, A.T. *et al.* GWAS of epigenetic aging rates in blood reveals a critical role for TERT. *Nat Commun* **9**, 387 (2018).

65. Kang, J.I. *et al.* Telomere length in alcohol dependence: A role for impulsive choice and childhood maltreatment. *Psychoneuroendocrinology* **83**, 72-78 (2017).

66. Vasconcelos-Moreno, M.P. *et al.* Telomere Length, Oxidative Stress, Inflammation and BDNF Levels in Siblings of Patients with Bipolar Disorder: Implications for Accelerated Cellular Aging. *Int J Neuropsychopharmacol* **20**, 445-454 (2017).

67. Kose Cinar, R. Telomere length and hTERT in mania and subsequent remission. *Rev Bras Psiquiatr*, 0 (2017).

68. Rao, S. *et al.* Accelerated leukocyte telomere erosion in schizophrenia: Evidence from the present study and a meta-analysis. *J Psychiatr Res* **79**, 50-56 (2016).

69. Xie, X. *et al.* Major depressive disorder mediates accelerated aging in rats subjected to chronic mild stress. *Behav Brain Res* **329**, 96-103 (2017).

70. Simons, C.J., van Winkel, R. & Group. Intermediate phenotype analysis of patients, unaffected siblings, and healthy controls identifies VMAT2 as a candidate gene for psychotic disorder and neurocognition. *Schizophr Bull* **39**, 848-56 (2013).

71. Willour, V.L. *et al.* Family-based association of FKBP5 in bipolar disorder. *Mol Psychiatry* **14**, 261-8 (2009).

72. Lavebratt, C., Aberg, E., Sjoholm, L.K. & Forsell, Y. Variations in FKBP5 and BDNF genes are suggestively associated with depression in a Swedish population-based cohort. *J Affect Disord* **125**, 249-55 (2010).

73. Binder, E.B. *et al.* Polymorphisms in FKBP5 are associated with increased recurrence of depressive episodes and rapid response to antidepressant treatment. *Nat Genet* **36**, 1319-25 (2004).

74. Brent, D. *et al.* Association of FKBP5 polymorphisms with suicidal events in the Treatment of Resistant Depression in Adolescents (TORDIA) study. *Am J Psychiatry* **167**, 190-7 (2010).

75. Szczepankiewicz, A. *et al.* FKBP5 polymorphism is associated with major depression but not with bipolar disorder. *J Affect Disord* **164**, 33-7 (2014).

76. Zobel, A. *et al.* DNA sequence variants of the FKBP5 gene are associated with unipolar depression. *Int J Neuropsychopharmacol* **13**, 649-60 (2010).

77. Bortsov, A.V. *et al.* Polymorphisms in the glucocorticoid receptor co-chaperone FKBP5 predict persistent musculoskeletal pain after traumatic stress exposure. *Pain* **154**, 1419-26 (2013).

78. Zorina-Lichtenwalter, K., Meloto, C.B., Khoury, S. & Diatchenko, L. Genetic predictors of human chronic pain conditions. *Neuroscience* **338**, 36-62 (2016).

79. Perroud, N. *et al.* Clinical and genetic correlates of suicidal ideation during antidepressant treatment in a depressed outpatient sample. *Pharmacogenomics* **12**, 365-77 (2011).

80. Supriyanto, I. *et al.* Association of FKBP5 gene haplotypes with completed suicide in the Japanese population. *Prog Neuropsychopharmacol Biol Psychiatry* **35**, 252-6 (2011).

81. Roy, A., Gorodetsky, E., Yuan, Q., Goldman, D. & Enoch, M.A. Interaction of FKBP5, a stress-related gene, with childhood trauma increases the risk for attempting suicide. *Neuropsychopharmacology* **35**, 1674-83 (2010).

82. Roy, A., Hodgkinson, C.A., Deluca, V., Goldman, D. & Enoch, M.A. Two HPA axis genes, CRHBP and FKBP5, interact with childhood trauma to increase the risk for suicidal behavior. *J Psychiatr Res* **46**, 72-9 (2012).

83. Yin, H. *et al.* Glucocorticoid Receptor-Related Genes: Genotype and Brain Gene Expression Relationships to Suicide and Major Depressive Disorder. *Depress Anxiety* **33**, 531-540 (2016).

84. Forero, D.A., Guio-Vega, G.P. & Gonzalez-Giraldo, Y. A comprehensive regional analysis of genome-wide expression profiles for major depressive disorder. *J Affect Disord* **218**, 86-92 (2017).

85. Perez-Ortiz, J.M., Garcia-Gutierrez, M.S., Navarrete, F., Giner, S. & Manzanares, J. Gene and protein alterations of FKBP5 and glucocorticoid receptor in the amygdala of suicide victims. *Psychoneuroendocrinology* **38**, 1251-8 (2013).

86. Kupfer, D.M., White, V.L., Strayer, D.L., Crouch, D.J. & Burian, D. Microarray characterization of gene expression changes in blood during acute ethanol exposure. *BMC Med Genomics* **6**, 26 (2013).

87. Levey, D.F. *et al.* Towards understanding and predicting suicidality in women: biomarkers and clinical risk assessment. *Mol Psychiatry* **21**, 768-85 (2016).

88. Niculescu, A.B. *et al.* Understanding and predicting suicidality using a combined genomic and clinical risk assessment approach. *Mol Psychiatry* **20**, 1266-85 (2015).

89. Hartmann, J. *et al.* Pharmacological Inhibition of the Psychiatric Risk Factor FKBP51 Has Anxiolytic Properties. *J Neurosci* **35**, 9007-16 (2015).

90. Bell, R.L. *et al.* Gene expression changes in the nucleus accumbens of alcohol-preferring rats following chronic ethanol consumption. *Pharmacol Biochem Behav* **94**, 131-47 (2009).

91. Gandal, M.J. *et al.* Shared molecular neuropathology across major psychiatric disorders parallels polygenic overlap. *Science* **359**, 693-697 (2018).

92. Gonzalez, D.A. *et al.* The Arf6 activator Efa6/PSD3 confers regional specificity and modulates ethanol consumption in Drosophila and humans. *Mol Psychiatry* (2017).

93. Uhl, G.R. *et al.* Genome-wide association for methamphetamine dependence: convergent results from 2 samples. *Arch Gen Psychiatry* **65**, 345-55 (2008).

94. Ma, D. *et al.* A genome-wide association study of autism reveals a common novel risk locus at 5p14.1. *Ann Hum Genet* **73**, 263-73 (2009).

95. Athanasiu, L. *et al.* Gene variants associated with schizophrenia in a Norwegian genome-wide study are replicated in a large European cohort. *J Psychiatr Res* **44**, 748-53 (2010).

96. Mozhui, K. *et al.* Genetic regulation of Nrxn1 [corrected] expression: an integrative cross-species analysis of schizophrenia candidate genes. *Transl Psychiatry* **1**, e25 (2011).

97. Chang, L.C. *et al.* A conserved BDNF, glutamate- and GABA-enriched gene module related to human depression identified by coexpression meta-analysis and DNA variant genome-wide association studies. *PLoS One* **9**, e90980 (2014).

98. Schlauch, K.A. *et al.* Genome-wide association analysis identifies genetic variations in subjects with myalgic encephalomyelitis/chronic fatigue syndrome. *Transl Psychiatry* **6**, e730 (2016).

99. Enwright Iii, J.F. *et al.* Transcriptome alterations of prefrontal cortical parvalbumin neurons in schizophrenia. *Mol Psychiatry* (2017).

100. Niculescu, A.B. *et al.* Precision medicine for suicidality: from universality to subtypes and personalization. *Mol Psychiatry* **22**, 1250-1273 (2017).

101. Gonzalez, D.A. *et al.* The Arf6 activator Efa6/PSD3 confers regional specificity and modulates ethanol consumption in Drosophila and humans. *Mol Psychiatry* **23**, 621-628 (2018).

102. McBride, W.J. *et al.* Gene expression in the ventral tegmental area of 5 pairs of rat lines selectively bred for high or low ethanol consumption. *Pharmacol Biochem Behav* **102**, 275-85 (2012).

103. Janeczek, P., MacKay, R.K., Lea, R.A., Dodd, P.R. & Lewohl, J.M. Reduced expression of alpha-synuclein in alcoholic brain: influence of SNCA-Rep1 genotype. *Addict Biol* **19**, 509-15 (2014).

104. Foroud, T. *et al.* Association of alcohol craving with alpha-synuclein (SNCA). *Alcohol Clin Exp Res* **31**, 537-45 (2007).

105. Kobayashi, H. *et al.* Study of association between alpha-synuclein gene polymorphism and methamphetamine psychosis/dependence. *Ann N Y Acad Sci* **1025**, 325-34 (2004).

106. Mata, I.F. *et al.* SNCA variant associated with Parkinson disease and plasma alpha-synuclein level. *Arch Neurol* **67**, 1350-6 (2010).

107. Chang, X. *et al.* RNA-seq analysis of amygdala tissue reveals characteristic expression profiles in schizophrenia. *Transl Psychiatry* **7**, e1203 (2017).

108. Chen, H. *et al.* Gene expression alterations in bipolar disorder postmortem brains. *Bipolar Disord* **15**, 177-87 (2013).

109. Beyer, K. *et al.* Low alpha-synuclein 126 mRNA levels in dementia with Lewy bodies and Alzheimer disease. *Neuroreport* **17**, 1327-30 (2006).

110. Mayfield, R.D. *et al.* Patterns of gene expression are altered in the frontal and motor cortices of human alcoholics. *J Neurochem* **81**, 802-13 (2002).

111. Lewohl, J.M. *et al.* The application of proteomics to the human alcoholic brain. *Ann N Y Acad Sci* **1025**, 14-26 (2004).

112. Martins-de-Souza, D. *et al.* Identification of proteomic signatures associated with depression and psychotic depression in post-mortem brains from major depression patients. *Transl Psychiatry* **2**, e87 (2012).

113. Sainz, J. *et al.* Inflammatory and immune response genes have significantly altered expression in schizophrenia. *Mol Psychiatry* **18**, 1056-7 (2013).

114. Kuzman, M.R., Medved, V., Terzic, J. & Krainc, D. Genome-wide expression analysis of peripheral blood identifies candidate biomarkers for schizophrenia. *J Psychiatr Res* **43**, 1073-7 (2009).

115. Liang, T. *et al.* Candidate genes for alcohol preference identified by expression profiling in alcohol-preferring and -nonpreferring reciprocal congenic rats. *Genome Biol* **11**, R11 (2010).

116. Mukherjee, S. *et al.* Knockdown of Clock in the ventral tegmental area through RNA interference results in a mixed state of mania and depression-like behavior. *Biol Psychiatry* **68**, 503-11 (2010).

117. Le-Niculescu, H. *et al.* Identifying blood biomarkers for mood disorders using convergent functional genomics. *Mol Psychiatry* **14**, 156-74 (2009).

118. Gottschalk, M.G., Wesseling, H., Guest, P.C. & Bahn, S. Proteomic enrichment analysis of psychotic and affective disorders reveals common signatures in presynaptic glutamatergic signaling and energy metabolism. *Int J Neuropsychopharmacol* **18**(2014).

119. Gruber, H.E., Hoelscher, G.L., Ingram, J.A. & Hanley, E.N., Jr. Genome-wide analysis of pain-, nerve- and neurotrophin -related gene expression in the degenerating human annulus. *Mol Pain* **8**, 63 (2012).

120. Lesscher, H.M., Houthuijzen, J.M., Groot Koerkamp, M.J., Holstege, F.C. & Vanderschuren, L.J. Amygdala 14-3-3zeta as a novel modulator of escalating alcohol intake in mice. *PLoS One* **7**, e37999 (2012).

121. Le-Niculescu, H. *et al.* Towards understanding the schizophrenia code: an expanded convergent functional genomics approach. *Am J Med Genet B Neuropsychiatr Genet* **144B**, 129-58 (2007).

122. Levine, M.E. & Crimmins, E.M. A Genetic Network Associated With Stress Resistance, Longevity, and Cancer in Humans. *J Gerontol A Biol Sci Med Sci* **71**, 703-12 (2016).

123. Chu, T.T., Liu, Y. & Kemether, E. Thalamic transcriptome screening in three psychiatric states. *J Hum Genet* **54**, 665-75 (2009).

124. Kalsi, G. *et al.* A systematic gene-based screen of chr4q22-q32 identifies association of a novel susceptibility gene, DKK2, with the quantitative trait of alcohol dependence symptom counts. *Hum Mol Genet* **19**, 2497-506 (2010).

125. Iossifov, I. *et al.* The contribution of de novo coding mutations to autism spectrum disorder. *Nature* **515**, 216-21 (2014).

126. Iwamoto, K., Bundo, M. & Kato, T. Altered expression of mitochondria-related genes in postmortem brains of patients with bipolar disorder or schizophrenia, as revealed by large-scale DNA microarray analysis. *Hum Mol Genet* **14**, 241-53 (2005).

127. Garbett, K.A. *et al.* Fibroblasts from patients with major depressive disorder show distinct transcriptional response to metabolic stressors. *Transl Psychiatry* **5**, e523 (2015).

128. Lind, P.A. *et al.* A genomewide association study of nicotine and alcohol dependence in Australian and Dutch populations. *Twin Res Hum Genet* **13**, 10-29 (2010).

129. Fromer, M. *et al.* Gene expression elucidates functional impact of polygenic risk for schizophrenia. *Nat Neurosci* **19**, 1442-1453 (2016).

130. Le-Niculescu, H. *et al.* Convergent functional genomics of anxiety disorders: translational identification of genes, biomarkers, pathways and mechanisms. *Transl Psychiatry* **1**, e9 (2011).

131. Gulsuner, S. *et al.* Spatial and temporal mapping of de novo mutations in schizophrenia to a fetal prefrontal cortical network. *Cell* **154**, 518-29 (2013).

132. Parikshak, N.N. *et al.* Genome-wide changes in lncRNA, splicing, and regional gene expression patterns in autism. *Nature* **540**, 423-427 (2016).

133. Passtoors, W.M. *et al.* Gene expression analysis of mTOR pathway: association with human longevity. *Aging Cell* **12**, 24-31 (2013).

134. Shibata, T. *et al.* The alteration of hypoxia inducible factor-1 (HIF-1) and its target genes in mood disorder patients. *Prog Neuropsychopharmacol Biol Psychiatry* **43**, 222-9 (2013).

135. Borovecki, F. *et al.* Genome-wide expression profiling of human blood reveals biomarkers for Huntington's disease. *Proc Natl Acad Sci U S A* **102**, 11023-8 (2005).

136. Rimondini, R., Arlinde, C., Sommer, W. & Heilig, M. Long-lasting increase in voluntary ethanol consumption and transcriptional regulation in the rat brain after intermittent exposure to alcohol. *Faseb J* **16**, 27-35 (2002).

137. Kishimoto, M. *et al.* The dysbindin gene (DTNBP1) is associated with methamphetamine psychosis. *Biol Psychiatry* **63**, 191-6 (2008).

138. Cheah, S.Y., Lawford, B.R., Young, R.M., Morris, C.P. & Voisey, J. Dysbindin (DTNBP1) variants are associated with hallucinations in schizophrenia. *Eur Psychiatry* **30**, 486-91 (2015).

139. Fatjo-Vilas, M. *et al.* Dysbindin-1 gene contributes differentially to early- and adult-onset forms of functional psychosis. *Am J Med Genet B Neuropsychiatr Genet* **156B**, 322-33 (2011).

140. Bernardo, M. *et al.* Modelling gene-environment interaction in first episodes of psychosis. *Schizophr Res* **189**, 181-189 (2017).

141. Schwab, S.G. *et al.* Support for association of schizophrenia with genetic variation in the 6p22.3 gene, dysbindin, in sib-pair families with linkage and in an additional sample of triad families. *Am J Hum Genet* **72**, 185-90 (2003).

142. Fallin, M.D. *et al.* Bipolar I disorder and schizophrenia: a 440-single-nucleotide polymorphism screen of 64 candidate genes among Ashkenazi Jewish case-parent trios. *Am J Hum Genet* **77**, 918-36 (2005).

143. Wirgenes, K.V. *et al.* Dysbindin and d-amino-acid-oxidase gene polymorphisms associated with positive and negative symptoms in schizophrenia. *Neuropsychobiology* **60**, 31-6 (2009).

144. Rethelyi, J.M. *et al.* Association study of NRG1, DTNBP1, RGS4, G72/G30, and PIP5K2A with schizophrenia and symptom severity in a Hungarian sample. *Am J Med Genet B Neuropsychiatr Genet* **153B**, 792-801 (2010).

145. Voisey, J. *et al.* Analysis of HapMap tag-SNPs in dysbindin (DTNBP1) reveals evidence of consistent association with schizophrenia. *Eur Psychiatry* **25**, 314-9 (2010).

146. Raybould, R. *et al.* Bipolar disorder and polymorphisms in the dysbindin gene (DTNBP1). *Biol Psychiatry* **57**, 696-701 (2005).

147. Breen, G. *et al.* Association of the dysbindin gene with bipolar affective disorder. *Am J Psychiatry* **163**, 1636-8 (2006).

148. Pae, C.U. *et al.* Effect of 5-haplotype of dysbindin gene (DTNBP1) polymorphisms for the susceptibility to bipolar I disorder. *Am J Med Genet B Neuropsychiatr Genet* **144B**, 701-3 (2007).

149. Joo, E.J. *et al.* Dysbindin gene variants are associated with bipolar I disorder in a Korean population. *Neurosci Lett* **418**, 272-5 (2007).

150. Wellcome Trust Case Control, C. Genome-wide association study of 14,000 cases of seven common diseases and 3,000 shared controls. *Nature* **447**, 661-78 (2007).

151. Gaysina, D. *et al.* Association of the dystrobrevin binding protein 1 gene (DTNBP1) in a bipolar case-control study (BACCS). *Am J Med Genet B Neuropsychiatr Genet* **150B**, 836-44 (2009).

152. Weickert, C.S. *et al.* Human dysbindin (DTNBP1) gene expression in normal brain and in schizophrenic prefrontal cortex and midbrain. *Arch Gen Psychiatry* **61**, 544-55 (2004).

153. Weickert, C.S., Rothmond, D.A., Hyde, T.M., Kleinman, J.E. & Straub, R.E. Reduced DTNBP1 (dysbindin-1) mRNA in the hippocampal formation of schizophrenia patients. *Schizophr Res* **98**, 105-10 (2008).

154. Talbot, K. *et al.* Synaptic dysbindin-1 reductions in schizophrenia occur in an isoform-specific manner indicating their subsynaptic location. *PLoS One* **6**, e16886 (2011).

155. Chagnon, Y.C., Roy, M.A., Bureau, A., Merette, C. & Maziade, M. Differential RNA expression between schizophrenic patients and controls of the dystrobrevin binding protein 1 and neuregulin 1 genes in immortalized lymphocytes. *Schizophr Res* **100**, 281-90 (2008).

156. Carr, G.V., Jenkins, K.A., Weinberger, D.R. & Papaleo, F. Loss of dysbindin-1 in mice impairs reward-based operant learning by increasing impulsive and compulsive behavior. *Behav Brain Res* **241**, 173-84 (2013).

157. Chen, X.W. *et al.* DTNBP1, a schizophrenia susceptibility gene, affects kinetics of transmitter release. *J Cell Biol* **181**, 791-801 (2008).

158. Hattori, S. *et al.* Behavioral abnormalities and dopamine reductions in sdy mutant mice with a deletion in Dtnbp1, a susceptibility gene for schizophrenia. *Biochem Biophys Res Commun* **373**, 298-302 (2008).

159. Feng, Y.Q. *et al.* Dysbindin deficiency in sandy mice causes reduction of snapin and displays behaviors related to schizophrenia. *Schizophr Res* **106**, 218-28 (2008).

160. Bhardwaj, S.K. *et al.* Behavioral characterization of dysbindin-1 deficient sandy mice. *Behav Brain Res* **197**, 435-41 (2009).

161. Talbot, K. The sandy (sdy) mouse: a dysbindin-1 mutant relevant to schizophrenia research. *Prog Brain Res* **179**, 87-94 (2009).

162. Kobayashi, K. *et al.* Correlated alterations in serotonergic and dopaminergic modulations at the hippocampal mossy fiber synapse in mice lacking dysbindin. *PLoS One* **6**, e18113 (2011).

163. Bhardwaj, S.K., Stojkovic, K., Kiessling, S., Srivastava, L.K. & Cermakian, N. Constant light uncovers behavioral effects of a mutation in the schizophrenia risk gene Dtnbp1 in mice. *Behav Brain Res* **284**, 58-68 (2015).

164. Takao, K. *et al.* Impaired long-term memory retention and working memory in sdy mutant mice with a deletion in Dtnbp1, a susceptibility gene for schizophrenia. *Mol Brain* **1**, 11 (2008).

165. Murotani, T. *et al.* High dopamine turnover in the brains of Sandy mice. *Neurosci Lett* **421**, 47-51 (2007).

166. Elia, J. *et al.* Rare structural variants found in attention-deficit hyperactivity disorder are preferentially associated with neurodevelopmental genes. *Mol Psychiatry* **15**, 637-46 (2010).

167. Lamers, F. *et al.* Serum proteomic profiles of depressive subtypes. *Transl Psychiatry* **6**, e851 (2016).

168. Ditzen, C. *et al.* Cerebrospinal fluid biomarkers for major depression confirm relevance of associated pathophysiology. *Neuropsychopharmacology* **37**, 1013-25 (2012).

169. Olausson, P., Ghafouri, B., Backryd, E. & Gerdle, B. Clear differences in cerebrospinal fluid proteome between women with chronic widespread pain and healthy women - a multivariate explorative cross-sectional study. *J Pain Res* **10**, 575-590 (2017).

170. Terao, A. *et al.* Immune response gene expression increases in the aging murine hippocampus. *J Neuroimmunol* **132**, 99-112 (2002).

171. Lachuer, J., Ouyang, L., Legras, C., Del Rio, J. & Barlow, C. Gene expression profiling reveals an inflammatory process in the anx/anx mutant mice. *Brain Res Mol Brain Res* **139**, 372-6 (2005).

172. Rao, A.R., Yourshaw, M., Christensen, B., Nelson, S.F. & Kerner, B. Rare deleterious mutations are associated with disease in bipolar disorder families. *Mol Psychiatry* **22**, 1009-1014 (2017).

173. Ivanova, R. *et al.* HLA-DR alleles display sex-dependent effects on survival and discriminate between individual and familial longevity. *Hum Mol Genet* **7**, 187-94 (1998).

174. Akisaka, M., Suzuki, M. & Inoko, H. Molecular genetic studies on DNA polymorphism of the HLA class II genes associated with human longevity. *Tissue Antigens* **50**, 489-93 (1997).

175. Listi, F. *et al.* HLA and KIR frequencies in Sicilian Centenarians. *Rejuvenation Res* **13**, 314-8 (2010).

176. Wang, X. *et al.* Genetic determinants of disease progression in Alzheimer's disease. *J Alzheimers Dis* **43**, 649-55 (2015).

177. International Schizophrenia, C. *et al.* Common polygenic variation contributes to risk of schizophrenia and bipolar disorder. *Nature* **460**, 748-52 (2009).

178. Shimada-Sugimoto, M. *et al.* Immune-related pathways including HLA-DRB1( *)13:02 are associated with panic disorder. *Brain Behav Immun* **46**, 96-103 (2015).

179. Glatt, S.J. *et al.* Comparative gene expression analysis of blood and brain provides concurrent validation of SELENBP1 up-regulation in schizophrenia. *Proc Natl Acad Sci U S A* **102**, 15533-8 (2005).

180. Lewohl, J.M. *et al.* Gene expression in human alcoholism: microarray analysis of frontal cortex. *Alcohol Clin Exp Res* **24**, 1873-82 (2000).

181. Flatscher-Bader, T. *et al.* Alcohol-responsive genes in the frontal cortex and nucleus accumbens of human alcoholics. *J Neurochem* **93**, 359-70 (2005).

182. Rodd, Z.A. *et al.* Candidate genes, pathways and mechanisms for alcoholism: an expanded convergent functional genomics approach. *Pharmacogenomics J* **7**, 222-56 (2007).

183. Shi, J. *et al.* Common variants on chromosome 6p22.1 are associated with schizophrenia. *Nature* **460**, 753-7 (2009).

184. Schizophrenia Working Group of the Psychiatric Genomics, C. Biological insights from 108 schizophrenia-associated genetic loci. *Nature* **511**, 421-7 (2014).

185. O'Dushlaine, C. *et al.* Molecular pathways involved in neuronal cell adhesion and membrane scaffolding contribute to schizophrenia and bipolar disorder susceptibility. *Mol Psychiatry* **16**, 286-92 (2011).

186. Ruderfer, D.M. *et al.* Polygenic dissection of diagnosis and clinical dimensions of bipolar disorder and schizophrenia. *Mol Psychiatry* **19**, 1017-1024 (2014).

187. Ripke, S. *et al.* Genome-wide association analysis identifies 13 new risk loci for schizophrenia. *Nat Genet* **45**, 1150-9 (2013).

188. Romme, I.A., de Reus, M.A., Ophoff, R.A., Kahn, R.S. & van den Heuvel, M.P. Connectome Disconnectivity and Cortical Gene Expression in Patients With Schizophrenia. *Biol Psychiatry* **81**, 495-502 (2017).

189. Hou, L. *et al.* Genome-wide association study of 40,000 individuals identifies two novel loci associated with bipolar disorder. *Hum Mol Genet* **25**, 3383-3394 (2016).

190. Psychiatric, G.C.B.D.W.G. Large-scale genome-wide association analysis of bipolar disorder identifies a new susceptibility locus near ODZ4. *Nat Genet* **43**, 977-83 (2011).

191. Nurnberger, J.I., Jr. *et al.* Identification of pathways for bipolar disorder: a meta-analysis. *JAMA Psychiatry* **71**, 657-64 (2014).

192. Belmonte Mahon, P. *et al.* Genome-wide association analysis of age at onset and psychotic symptoms in bipolar disorder. *Am J Med Genet B Neuropsychiatr Genet* **156B**, 370-8 (2011).

193. Cichon, S. *et al.* Genome-wide association study identifies genetic variation in neurocan as a susceptibility factor for bipolar disorder. *Am J Hum Genet* **88**, 372-81 (2011).

194. Andreassen, O.A. *et al.* Improved detection of common variants associated with schizophrenia and bipolar disorder using pleiotropy-informed conditional false discovery rate. *PLoS Genet* **9**, e1003455 (2013).

195. Trost, S. *et al.* Investigating the Impact of a Genome-Wide Supported Bipolar Risk Variant of MAD1L1 on the Human Reward System. *Neuropsychopharmacology* **41**, 2679-87 (2016).

196. Fernandez-Castillo, N. *et al.* Transcriptomic and genetic studies identify NFAT5 as a candidate gene for cocaine dependence. *Transl Psychiatry* **5**, e667 (2015).

197. Beech, R.D. *et al.* Increased peripheral blood expression of electron transport chain genes in bipolar depression. *Bipolar Disord* **12**, 813-24 (2010).

198. Kang, H.J. *et al.* Gene expression profiling in postmortem prefrontal cortex of major depressive disorder. *J Neurosci* **27**, 13329-40 (2007).

199. Sequeira, A. *et al.* Gene expression changes in the prefrontal cortex, anterior cingulate cortex and nucleus accumbens of mood disorders subjects that committed suicide. *PLoS One* **7**, e35367 (2012).

200. Kim, K.H. *et al.* Transcriptomic Analysis of Induced Pluripotent Stem Cells Derived from Patients with Bipolar Disorder from an Old Order Amish Pedigree. *PLoS One* **10**, e0142693 (2015).

201. McClintick, J.N. *et al.* Stress-response pathways are altered in the hippocampus of chronic alcoholics. *Alcohol* **47**, 505-15 (2013).

202. Maron, E. *et al.* Peripheral gene expression profiling of CCK-4-induced panic in healthy subjects. *Am J Med Genet B Neuropsychiatr Genet* **153B**, 269-74 (2010).

203. Maes, O.C. *et al.* Transcriptional profiling of Alzheimer blood mononuclear cells by microarray. *Neurobiol Aging* **28**, 1795-809 (2007).

204. Cole, S.W. *et al.* Loneliness, eudaimonia, and the human conserved transcriptional response to adversity. *Psychoneuroendocrinology* **62**, 11-7 (2015).

205. Bagot, R.C. *et al.* Circuit-wide Transcriptional Profiling Reveals Brain Region-Specific Gene Networks Regulating Depression Susceptibility. *Neuron* **90**, 969-83 (2016).

206. Philibert, R.A. *et al.* Transcriptional profiling of lymphoblast lines from subjects with panic disorder. *Am J Med Genet B Neuropsychiatr Genet* **144B**, 674-82 (2007).

207. Begemann, M. *et al.* Episode-specific differential gene expression of peripheral blood mononuclear cells in rapid cycling supports novel treatment approaches. *Mol Med* **14**, 546-52 (2008).

208. Hwang, Y. *et al.* Gene expression profiling by mRNA sequencing reveals increased expression of immune/inflammation-related genes in the hippocampus of individuals with schizophrenia. *Transl Psychiatry* **3**, e321 (2013).

209. Pantazatos, S.P. *et al.* Whole-transcriptome brain expression and exon-usage profiling in major depression and suicide: evidence for altered glial, endothelial and ATPase activity. *Mol Psychiatry* **22**, 760-773 (2017).

210. Leighton, S.P. *et al.* Chemokines in depression in health and in inflammatory illness: a systematic review and meta-analysis. *Mol Psychiatry* **23**, 48-58 (2018).

211. Janelidze, S. *et al.* Altered chemokine levels in the cerebrospinal fluid and plasma of suicide attempters. *Psychoneuroendocrinology* **38**, 853-62 (2013).

212. Freeman, W.M., Salzberg, A.C., Gonzales, S.W., Grant, K.A. & Vrana, K.E. Classification of alcohol abuse by plasma protein biomarkers. *Biol Psychiatry* **68**, 219-22 (2010).

213. Gaiteri, C., Guilloux, J.P., Lewis, D.A. & Sibille, E. Altered gene synchrony suggests a combined hormone-mediated dysregulated state in major depression. *PLoS One* **5**, e9970 (2010).

214. Lo, C.L. *et al.* High Resolution Genomic Scans Reveal Genetic Architecture Controlling Alcohol Preference in Bidirectionally Selected Rat Model. *PLoS Genet* **12**, e1006178 (2016).

215. Dempster, E.L. *et al.* Disease-associated epigenetic changes in monozygotic twins discordant for schizophrenia and bipolar disorder. *Hum Mol Genet* **20**, 4786-96 (2011).

216. Sokolowski, M., Wasserman, J. & Wasserman, D. Polygenic associations of neurodevelopmental genes in suicide attempt. *Mol Psychiatry* **21**, 1381-90 (2016).

217. Kurian, S.M. *et al.* Identification of blood biomarkers for psychosis using convergent functional genomics. *Mol Psychiatry* **16**, 37-58 (2011).

218. Guo, W. *et al.* Interaction among genes influencing ethanol metabolism and sex is association with alcohol use disorders in a Tibet population. *Am J Med Genet B Neuropsychiatr Genet* **153B**, 561-569 (2010).

219. Huo, R. *et al.* Genetic polymorphisms in CYP2E1: association with schizophrenia susceptibility and risperidone response in the Chinese Han population. *PLoS One* **7**, e34809 (2012).

220. Le-Niculescu, H. *et al.* Discovery and validation of blood biomarkers for suicidality. *Mol Psychiatry* **18**, 1249-64 (2013).

221. Jamain, S. *et al.* Common and rare variant analysis in early-onset bipolar disorder vulnerability. *PLoS One* **9**, e104326 (2014).

222. McCarthy, S.E. *et al.* De novo mutations in schizophrenia implicate chromatin remodeling and support a genetic overlap with autism and intellectual disability. *Mol Psychiatry* **19**, 652-8 (2014).

223. Kanazawa, T. *et al.* Genome-wide association study of atypical psychosis. *Am J Med Genet B Neuropsychiatr Genet* **162B**, 679-86 (2013).

224. Barden, A. *et al.* n-3 Fatty Acid Supplementation and Leukocyte Telomere Length in Patients with Chronic Kidney Disease. *Nutrients* **8**, 175 (2016).

225. Rangaraju, S. *et al.* Atypical antidepressants extend lifespan of Caenorhabditis elegans by activation of a non-cell-autonomous stress response. *Aging Cell* **14**, 971-81 (2015).

226. Powell, T.R., Dima, D., Frangou, S. & Breen, G. Telomere Length and Bipolar Disorder. *Neuropsychopharmacology* **43**, 445-453 (2018).

227. Martinsson, L. *et al.* Long-term lithium treatment in bipolar disorder is associated with longer leukocyte telomeres. *Transl Psychiatry* **3**, e261 (2013).

228. Monroy-Jaramillo, N. *et al.* Leukocyte telomere length in Hispanic schizophrenia patients under treatment with olanzapine. *J Psychiatr Res* **90**, 26-30 (2017).

229. Hoge, E.A. *et al.* Loving-Kindness Meditation practice associated with longer telomeres in women. *Brain Behav Immun* **32**, 159-63 (2013).

230. Jacobs, T.L. *et al.* Intensive meditation training, immune cell telomerase activity, and psychological mediators. *Psychoneuroendocrinology* **36**, 664-81 (2011).

231. McQuillin, A., Rizig, M. & Gurling, H.M. A microarray gene expression study of the molecular pharmacology of lithium carbonate on mouse brain mRNA to understand the neurobiology of mood stabilization and treatment of bipolar affective disorder. *Pharmacogenet Genomics* **17**, 605-17 (2007).

232. Ogden, C.A. *et al.* Candidate genes, pathways and mechanisms for bipolar (manic-depressive) and related disorders: an expanded convergent functional genomics approach. *Mol Psychiatry* **9**, 1007-29 (2004).

233. Breen, M.S. *et al.* Lithium-responsive genes and gene networks in bipolar disorder patient-derived lymphoblastoid cell lines. *Pharmacogenomics J* **16**, 446-53 (2016).

234. Hill, E.J. *et al.* Effects of lithium and valproic acid on gene expression and phenotypic markers in an NT2 neurosphere model of neural development. *PLoS One* **8**, e58822 (2013).

235. Martins-de-Souza, D. *et al.* Blood mononuclear cell proteome suggests integrin and Ras signaling as critical pathways for antidepressant treatment response. *Biol Psychiatry* **76**, e15-7 (2014).

236. Sugawara, H. *et al.* Effect of mood stabilizers on gene expression in lymphoblastoid cells. *J Neural Transm (Vienna)* **117**, 155-64 (2010).

237. Rangaraju, S. *et al.* Mood, stress and longevity: convergence on ANK3. *Mol Psychiatry* **21**, 1037-49 (2016).

238. Hennings, J.M. *et al.* RNA expression profiling in depressed patients suggests retinoid-related orphan receptor alpha as a biomarker for antidepressant response. *Transl Psychiatry* **5**, e538 (2015).
