# Supplementary material for: Towards precision medicine for stress disorders: diagnostic biomarkers and targeted drugs
Source: Mol Psychiatry. 2019 Mar 12;25(5):918–38. doi: 10.1038/s41380-019-0370-z (PMC7192849; doi:10.1038/s41380-019-0370-z)
Supplement: Supplementary file 2 — Supplementary Information - Detailed Demographics Table S1 [file 41380_2019_370_MOESM2_ESM.docx]

| **Table S1 Detailed Demographics** | | | | | | | | | |
| --- | --- | --- | --- | --- | --- | --- | --- | --- | --- |
|  | **A. Independent Discovery Cohort (n=36) (91 visits)** | | | | | | | | |
| Participant ID visit number | Diagnosis | Gender  (M/F) | Age at testing (Years) | Ethnicity | VAS  SSS1 Life Stress (1-100) | **Discovery**  **Cohort** | Test Cohort Stress State (VAS) | Test Cohort First Year Hospitalizations with Stress | Test Cohort All Future Hospitalizations with Stress |
| phchp052v2 | SZ | M | 60 | Caucasian | 86 | Yes | No | No | No |
| phchp052v3 | SZ | M | 60 | Caucasian | 17 | Yes | No | No | No |
| phchp109v1 | BP | M | 22 | Caucasian | 84 | Yes | No | No | No |
| phchp109v2 | BP | M | 25 | Caucasian | 0 | Yes | No | No | No |
| phchp124v1 | BP | M | 53 | Caucasian | 71 | Yes | No | No | No |
| phchp124v2 | BP | M | 54 | Caucasian | 11 | Yes | No | No | No |
| phchp132v1 | BP | M | 51 | Caucasian | 19 | Yes | No | No | No |
| phchp132v6 | BP | M | 55 | Caucasian | 74 | Yes | No | No | No |
| phchp134v2 | BP | M | 59 | Caucasian | 74 | Yes | No | No | No |
| phchp134v3 | BP | M | 59 | Caucasian | 2 | Yes | No | No | No |
| phchp134v4 | BP | M | 61 | Caucasian | 6 | Yes | No | No | No |
| phchp134v6 | BP | M | 62 | Caucasian | 6 | Yes | No | No | No |
| phchp151v1 | SZ | M | 24 | Caucasian | 95 | Yes | No | No | No |
| phchp151v2 | SZ | M | 24 | Caucasian | 2 | Yes | No | No | No |
| phchp153v1 | BP | M | 55 | Caucasian | 85 | Yes | No | No | No |
| phchp153v2 | BP | M | 55 | Caucasian | 98 | Yes | No | No | No |
| phchp153v4 | BP | M | 57 | Caucasian | 76 | Yes | No | No | No |
| phchp153v6 | BP | M | 58 | Caucasian | 2 | Yes | No | No | No |
| phchp154v1 | PSYCH | M | 51 | African American | 10 | Yes | No | No | No |
| phchp154v2 | PSYCH | M | 51 | African American | 76 | Yes | No | No | No |
| phchp154v3 | PSYCH | M | 52 | African American | 3 | Yes | No | No | No |
| phchp157v2 | BP | M | 57 | African American | 82 | Yes | No | No | No |
| phchp157v3 | BP | M | 58 | African American | 5 | Yes | No | No | No |
| phchp160v1 | SZA | F | 41 | Caucasian | 7 | Yes | No | No | No |
| phchp160v2 | SZA | F | 41 | Caucasian | 94 | Yes | No | No | No |
| phchp160v3 | SZA | F | 41 | Caucasian | 8 | Yes | No | No | No |
| phchp164v2 | MDD | F | 49 | Caucasian | 2 | Yes | No | No | No |
| phchp164v3 | MDD | F | 49 | Caucasian | 73 | Yes | No | No | No |
| phchp166v1 | BP | M | 56 | Caucasian | 75 | Yes | No | No | No |
| phchp166v2 | BP | M | 56 | Caucasian | 21 | Yes | No | No | No |
| phchp166v4 | BP | M | 58 | Caucasian | 5 | Yes | No | No | No |
| phchp173v1 | MDD | M | 48 | Caucasian | 72 | Yes | No | No | No |
| phchp173v2 | MDD | M | 49 | Caucasian | 4 | Yes | No | No | No |
| phchp176v1 | SZ | M | 23 | African American | 98 | Yes | No | No | No |
| phchp176v2 | SZ | M | 24 | African American | 29 | Yes | No | No | No |
| phchp193v1 | BP | M | 39 | Hispanic | 70 | Yes | No | No | No |
| phchp193v3 | BP | M | 39 | Hispanic | 16 | Yes | No | No | No |
| phchp193v4 | BP | M | 40 | Hispanic | 21 | Yes | No | No | No |
| phchp196v1 | MDD | M | 56 | African American | 80 | Yes | No | No | No |
| phchp196v3 | MDD | M | 57 | African American | 13 | Yes | No | No | No |
| phchp204v1 | BP | F | 49 | Caucasian | 17 | Yes | No | No | No |
| phchp204v2 | BP | F | 49 | Caucasian | 79 | Yes | No | No | No |
| phchp204v3 | BP | F | 49 | Caucasian | 72 | Yes | No | No | No |
| phchp209v1 | PTSD | M | 54 | African American | 10 | Yes | No | No | No |
| phchp209v3 | PTSD | M | 54 | African American | 76 | Yes | No | No | No |
| phchp213v2 | PTSD | M | 62 | Caucasian | 32 | Yes | No | No | No |
| phchp213v3 | PTSD | M | 62 | Caucasian | 78 | Yes | No | No | No |
| phchp214v1 | PTSD | F | 52 | Caucasian | 32 | Yes | No | No | No |
| phchp214v2 | PTSD | F | 53 | Caucasian | 81 | Yes | No | No | No |
| phchp214v3 | PTSD | F | 57 | Caucasian | 78 | Yes | No | No | No |
| phchp226v1 | MDD | M | 29 | Caucasian | 22 | Yes | No | No | No |
| phchp226v2 | MDD | M | 29 | Caucasian | 81 | Yes | No | No | No |
| phchp226v3 | MDD | M | 30 | Caucasian | 82 | Yes | No | No | No |
| phchp243v1 | PTSD | M | 50 | African American | 84 | Yes | No | No | No |
| phchp243v2 | PTSD | M | 50 | African American | 33 | Yes | No | No | No |
| phchp243v4 | PTSD | M | 54 | African American | 20 | Yes | No | No | No |
| phchp248v2 | SZ | M | 52 | African American | 88 | Yes | No | No | No |
| phchp248v5 | SZ | M | 55 | African American | 29 | Yes | No | No | No |
| phchp258v1 | BP | F | 52 | Caucasian | 0 | Yes | No | No | No |
| phchp258v3 | BP | F | 54 | Caucasian | 100 | Yes | No | No | No |
| phchp274v1 | BP | M | 48 | Caucasian | 82 | Yes | No | No | No |
| phchp274v2 | BP | M | 48 | Caucasian | 93 | Yes | No | No | No |
| phchp274v3 | BP | M | 48 | Caucasian | 9 | Yes | No | No | No |
| phchp274v4 | BP | M | 50 | Caucasian | 6 | Yes | No | No | No |
| phchp279v1 | SZ | M | 60 | African American | 88 | Yes | No | No | No |
| phchp279v2 | SZ | M | 61 | African American | 89 | Yes | No | No | No |
| phchp279v3 | SZ | M | 61 | African American | 4 | Yes | No | No | No |
| phchp279v4 | SZ | M | 61 | African American | 96 | Yes | No | No | No |
| phchp282v1 | SZ | M | 56 | Caucasian | 85 | Yes | No | No | No |
| phchp282v5 | SZ | M | 59 | Caucasian | 0 | Yes | No | No | No |
| phchp285v1 | BP | F | 56 | Caucasian | 13 | Yes | No | No | No |
| phchp285v3 | BP | F | 57 | Caucasian | 68 | Yes | No | No | No |
| phchp292v1 | BP | M | 42 | Caucasian | 0 | Yes | No | No | No |
| phchp292v2 | BP | M | 42 | Caucasian | 89 | Yes | No | No | No |
| phchp302v1 | BP | M | 61 | Caucasian | 0 | Yes | No | No | No |
| phchp302v3 | BP | M | 61 | Caucasian | 26 | Yes | No | No | No |
| phchp302v5 | BP | M | 62 | Caucasian | 84 | Yes | No | No | No |
| phchp329v2 | SZA | M | 50 | African American | 91 | Yes | No | No | No |
| phchp329v3 | SZA | M | 51 | African American | 4 | Yes | No | No | No |
| phchp333v1 | PTSD | M | 38 | Caucasian | 90 | Yes | No | No | No |
| phchp333v2 | PTSD | M | 38 | Caucasian | 87 | Yes | No | No | No |
| phchp333v3 | PTSD | M | 39 | Caucasian | 93 | Yes | No | No | No |
| phchp333v4 | PTSD | M | 39 | Caucasian | 12 | Yes | No | No | No |
| phchp340v1 | MDD | F | 51 | Caucasian | 77 | Yes | No | No | No |
| phchp340v3 | MDD | F | 52 | Caucasian | 31 | Yes | No | No | No |
| phchp351v1 | MDD | M | 44 | Caucasian | 2 | Yes | No | No | No |
| phchp351v2 | MDD | M | 44 | Caucasian | 69 | Yes | No | No | No |
| phchp361v1 | PTSD | F | 59 | African American | 89 | Yes | No | No | No |
| phchp361v3 | PTSD | F | 60 | African American | 0 | Yes | No | No | No |
| phchp362v2 | MDD | M | 54 | Caucasian | 14 | Yes | No | No | No |
| phchp362v3 | MDD | M | 55 | Caucasian | 79 | Yes | No | No | No |

| **B. Independent Validation Cohort with Clinically Severe Stress(n=48) (75 visits)** | | | | | | | | | | | |
| --- | --- | --- | --- | --- | --- | --- | --- | --- | --- | --- | --- |
| Participant ID visit | Diagnosis | Gender  (M/F) | Age at testing (Years) | Ethnicity | VAS) | PTSD  Scale  (PCL-C) | Discovery Cohort | **Validation cohort** | Test Cohort Stress State (VAS STRESS Scale) | Test Cohort First Year Hospitalizations with Stress | Test Cohort All Future Hospitalizations with Stress |
| phchp291v1 | SZ | F | 45 | Caucasian | 74 | 51 | No | Validation Clinically Severe Stress | No | No | No |
| phchp084v4 | BP | F | 57 | Caucasian | 85 | 61 | No | Validation Clinically Severe Stress | No | No | No |
| phchp089v4 | SZA | M | 38 | Caucasian | 67 | 50 | No | Validation Clinically Severe Stress | No | No | No |
| phchp096v1 | SZ | M | 55 | African American | 80 | 50 | No | Validation Clinically Severe Stress | No | No | No |
| phchp101v1 | SZA | M | 74 | Caucasian | 95 | 67 | No | Validation Clinically Severe Stress | No | No | No |
| phchp102v1 | SZA | M | 56 | Caucasian | 67 | 64 | No | Validation Clinically Severe Stress | No | No | No |
| phchp116v1 | SZA | M | 47 | Caucasian | 69 | 57 | No | Validation Clinically Severe Stress | No | No | No |
| phchp118v1 | SZA | M | 46 | African American | 98 | 59 | No | Validation Clinically Severe Stress | No | No | No |
| phchp118v4 | SZA | M | 50 | African American | 97 | 52 | No | Validation Clinically Severe Stress | No | No | No |
| phchp119v2 | SZA | M | 56 | African American | 74 | 58 | No | Validation Clinically Severe Stress | No | No | No |
| phchp121v2 | MOOD | M | 56 | Caucasian | 79 | 58 | No | Validation Clinically Severe Stress | No | No | No |
| phchp130v1 | MDD | F | 42 | Caucasian | 79 | 64 | No | Validation Clinically Severe Stress | No | No | No |
| phchp133v1 | SZ | M | 55 | Caucasian | 79 | 73 | No | Validation Clinically Severe Stress | No | No | No |
| phchp141v2 | BP | F | 47 | Caucasian | 99 | 55 | No | Validation Clinically Severe Stress | No | No | No |
| phchp141v3 | BP | F | 47 | Caucasian | 98 | 51 | No | Validation Clinically Severe Stress | No | No | No |
| phchp150v1 | SZA | M | 61 | Caucasian | 75 | 68 | No | Validation Clinically Severe Stress | No | No | No |
| phchp150v2 | SZA | M | 61 | Caucasian | 84 | 60 | No | Validation Clinically Severe Stress | No | No | No |
| phchp150v3 | SZA | M | 62 | Caucasian | 93 | 67 | No | Validation Clinically Severe Stress | No | No | No |
| phchp155v1 | MDD | M | 37 | Caucasian | 99 | 68 | No | Validation Clinically Severe Stress | No | No | No |
| phchp162v1 | MDD | M | 57 | Caucasian | 70 | 67 | No | Validation Clinically Severe Stress | No | No | No |
| phchp162v2 | MDD | M | 57 | Caucasian | 71 | 62 | No | Validation Clinically Severe Stress | No | No | No |
| phchp167v1 | MDD | M | 49 | Caucasian | 85 | 70 | No | Validation Clinically Severe Stress | No | No | No |
| phchp182v1 | MDD | M | 39 | Caucasian | 92 | 71 | No | Validation Clinically Severe Stress | No | No | No |
| phchp182v2 | MDD | M | 39 | Caucasian | 92 | 74 | No | Validation Clinically Severe Stress | No | No | No |
| phchp182v3 | MDD | M | 40 | Caucasian | 96 | 67 | No | Validation Clinically Severe Stress | No | No | No |
| phchp205v1 | PTSD | F | 54 | Caucasian | 81 | 56 | No | Validation Clinically Severe Stress | No | No | No |
| phchp205v2 | PTSD | F | 54 | Caucasian | 100 | 66 | No | Validation Clinically Severe Stress | No | No | No |
| phchp205v3 | PTSD | F | 54 | Caucasian | 100 | 79 | No | Validation Clinically Severe Stress | No | No | No |
| phchp215v1 | PTSD | F | 58 | Caucasian | 99 | 78 | No | Validation Clinically Severe Stress | No | No | No |
| phchp215v2 | PTSD | F | 58 | Caucasian | 77 | 54 | No | Validation Clinically Severe Stress | No | No | No |
| phchp216v1 | MOOD | M | 50 | African American | 73 | 68 | No | Validation Clinically Severe Stress | No | No | No |
| phchp216v2 | MOOD | M | 51 | African American | 90 | 76 | No | Validation Clinically Severe Stress | No | No | No |
| phchp231v1 | MDD | M | 55 | Caucasian | 97 | 53 | No | Validation Clinically Severe Stress | No | No | No |
| phchp234v1 | BP | M | 44 | Caucasian | 71 | 64 | No | Validation Clinically Severe Stress | No | No | No |
| phchp237v1 | PTSD | M | 39 | Caucasian | 89 | 79 | No | Validation Clinically Severe Stress | No | No | No |
| phchp266v1 | MOOD | M | 41 | Caucasian | 91 | 61 | No | Validation Clinically Severe Stress | No | No | No |
| phchp286v1 | BP | M | 54 | Caucasian | 70 | 56 | No | Validation Clinically Severe Stress | No | No | No |
| phchp289v1 | PTSD | F | 50 | Caucasian | 72 | 53 | No | Validation Clinically Severe Stress | No | No | No |
| phchp296v1 | BP | M | 48 | Caucasian | 87 | 55 | No | Validation Clinically Severe Stress | No | No | No |
| phchp296v2 | BP | M | 49 | Caucasian | 78 | 52 | No | Validation Clinically Severe Stress | No | No | No |
| phchp299v1 | PTSD | M | 54 | Caucasian | 73 | 66 | No | Validation Clinically Severe Stress | No | No | No |
| phchp299v2 | PTSD | M | 54 | Caucasian | 82 | 64 | No | Validation Clinically Severe Stress | No | No | No |
| phchp299v3 | PTSD | M | 54 | Caucasian | 86 | 74 | No | Validation Clinically Severe Stress | No | No | No |
| phchp304v1 | MDD | M | 52 | Caucasian | 84 | 58 | No | Validation Clinically Severe Stress | No | No | No |
| phchp307v1 | PTSD | F | 53 | Caucasian | 99 | 63 | No | Validation Clinically Severe Stress | No | No | No |
| phchp310v3 | MOOD | M | 54 | African American | 80 | 63 | No | Validation Clinically Severe Stress | No | No | No |
| phchp311v1 | MDD | F | 60 | African American | 72 | 74 | No | Validation Clinically Severe Stress | No | No | No |
| phchp313v1 | PTSD | M | 46 | African American | 77 | 77 | No | Validation Clinically Severe Stress | No | No | No |
| phchp313v2 | PTSD | M | 46 | African American | 82 | 66 | No | Validation Clinically Severe Stress | No | No | No |
| phchp313v3 | PTSD | M | 46 | African American | 89 | 72 | No | Validation Clinically Severe Stress | No | No | No |
| phchp316v1 | BP | M | 50 | Caucasian | 88 | 62 | No | Validation Clinically Severe Stress | No | No | No |
| phchp316v5 | BP | M | 51 | Caucasian | 74 | 85 | No | Validation Clinically Severe Stress | No | No | No |
| phchp316v6 | BP | M | 52 | Caucasian | 100 | 56 | No | Validation Clinically Severe Stress | No | No | No |
| phchp319v2 | PTSD | M | 42 | African American | 75 | 61 | No | Validation Clinically Severe Stress | No | No | No |
| phchp319v3 | PTSD | M | 43 | African American | 70 | 54 | No | Validation Clinically Severe Stress | No | No | No |
| phchp319v6 | PTSD | M | 44 | African American | 86 | 54 | No | Validation Clinically Severe Stress | No | No | No |
| phchp320v1 | BP | M | 58 | African American | 67 | 58 | No | Validation Clinically Severe Stress | No | No | No |
| phchp320v2 | BP | M | 58 | African American | 83 | 56 | No | Validation Clinically Severe Stress | No | No | No |
| phchp320v3 | BP | M | 59 | African American | 96 | 54 | No | Validation Clinically Severe Stress | No | No | No |
| phchp323v1 | PTSD | M | 32 | Caucasian | 92 | 74 | No | Validation Clinically Severe Stress | No | No | No |
| phchp323v2 | PTSD | M | 33 | Caucasian | 88 | 84 | No | Validation Clinically Severe Stress | No | No | No |
| phchp323v3 | PTSD | M | 33 | Caucasian | 94 | 78 | No | Validation Clinically Severe Stress | No | No | No |
| phchp325v2 | PTSD | M | 44 | Caucasian | 68 | 63 | No | Validation Clinically Severe Stress | No | No | No |
| phchp328v1 | MDD | F | 37 | Caucasian | 96 | 73 | No | Validation Clinically Severe Stress | No | No | No |
| phchp328v2 | MDD | F | 38 | Caucasian | 81 | 83 | No | Validation Clinically Severe Stress | No | No | No |
| phchp334v1 | BP | F | 50 | Caucasian | 94 | 62 | No | Validation Clinically Severe Stress | No | No | No |
| phchp337v3 | PTSD | M | 35 | Caucasian | 77 | 51 | No | Validation Clinically Severe Stress | No | No | No |
| phchp341v2 | MDD | M | 45 | Caucasian | 89 | 57 | No | Validation Clinically Severe Stress | No | No | No |
| phchp341v3 | MDD | M | 45 | Caucasian | 87 | 60 | No | Validation Clinically Severe Stress | No | No | No |
| phchp342v1 | MDD | M | 52 | Caucasian | 92 | 62 | No | Validation Clinically Severe Stress | No | No | No |
| phchp345v1 | PTSD | M | 33 | Caucasian | 90 | 75 | No | Validation Clinically Severe Stress | No | No | No |
| phchp345v3 | PTSD | M | 34 | Caucasian | 71 | 67 | No | Validation Clinically Severe Stress | No | No | No |
| phchp359v3 | PTSD | F | 57 | Caucasian | 71 | 58 | No | Validation Clinically Severe Stress | No | No | No |
| phchp363v2 | MDD | M | 48 | African American | 80 | 69 | No | Validation Clinically Severe Stress | No | No | No |
| phchp368v2 | MDD | F | 44 | Caucasian | 84 | 70 | No | Validation Clinically Severe Stress | No | No | No |
|  |  |  |  |  |  |  |  |  |  |  |  |
| phchp052v3 | SZ | M | 60 | Caucasian | 17 | 46 | Discovery Low Stress | No | No | No | No |
| phchp109v2 | BP | M | 25 | Caucasian | 0 | 63 | Discovery Low Stress | No | No | No | No |
| phchp124v2 | BP | M | 54 | Caucasian | 11 | 36 | Discovery Low Stress | No | No | No | No |
| phchp132v1 | BP | M | 51 | Caucasian | 19 | 41 | Discovery Low Stress | No | No | No | No |
| phchp134v3 | BP | M | 59 | Caucasian | 2 | 57 | Discovery Low Stress | No | No | No | No |
| phchp134v4 | BP | M | 61 | Caucasian | 6 | 31 | Discovery Low Stress | No | No | No | No |
| phchp134v6 | BP | M | 62 | Caucasian | 6 | 41 | Discovery Low Stress | No | No | No | No |
| phchp151v2 | SZ | M | 24 | Caucasian | 2 | 43 | Discovery Low Stress | No | No | No | No |
| phchp153v6 | BP | M | 58 | Caucasian | 2 | 21 | Discovery Low Stress | No | No | No | No |
| phchp154v1 | PSYCH | M | 51 | African American | 10 | 49 | Discovery Low Stress | No | No | No | No |
| phchp154v3 | PSYCH | M | 52 | African American | 3 | 27 | Discovery Low Stress | No | No | No | No |
| phchp157v3 | BP | M | 58 | African American | 5 | 37 | Discovery Low Stress | No | No | No | No |
| phchp160v1 | SZA | F | 41 | Caucasian | 7 | 41 | Discovery Low Stress | No | No | No | No |
| phchp160v3 | SZA | F | 41 | Caucasian | 8 | 66 | Discovery Low Stress | No | No | No | No |
| phchp164v2 | MDD | F | 49 | Caucasian | 2 | 22 | Discovery Low Stress | No | No | No | No |
| phchp166v2 | BP | M | 56 | Caucasian | 21 | 55 | Discovery Low Stress | No | No | No | No |
| phchp166v4 | BP | M | 58 | Caucasian | 5 | 58 | Discovery Low Stress | No | No | No | No |
| phchp173v2 | MDD | M | 49 | Caucasian | 4 | 17 | Discovery Low Stress | No | No | No | No |
| phchp176v2 | SZ | M | 24 | African American | 29 | 29 | Discovery Low Stress | No | No | No | No |
| phchp193v3 | BP | M | 39 | Hispanic | 16 | 24 | Discovery Low Stress | No | No | No | No |
| phchp193v4 | BP | M | 40 | Hispanic | 21 | 29 | Discovery Low Stress | No | No | No | No |
| phchp196v3 | MDD | M | 57 | African American | 13 | 28 | Discovery Low Stress | No | No | No | No |
| phchp204v1 | BP | F | 49 | Caucasian | 17 | 42 | Discovery Low Stress | No | No | No | No |
| phchp209v1 | PTSD | M | 54 | African American | 10 | 26 | Discovery Low Stress | No | No | No | No |
| phchp213v2 | PTSD | M | 62 | Caucasian | 32 | 68 | Discovery Low Stress | No | No | No | No |
| phchp214v1 | PTSD | F | 52 | Caucasian | 32 | 49 | Discovery Low Stress | No | No | No | No |
| phchp226v1 | MDD | M | 29 | Caucasian | 22 | 37 | Discovery Low Stress | No | No | No | No |
| phchp243v2 | PTSD | M | 50 | African American | 33 | 40 | Discovery Low Stress | No | No | No | No |
| phchp243v4 | PTSD | M | 54 | African American | 20 | 44 | Discovery Low Stress | No | No | No | No |
| phchp248v5 | SZ | M | 55 | African American | 29 | 69 | Discovery Low Stress | No | No | No | No |
| phchp258v1 | BP | F | 52 | Caucasian | 0 | 52 | Discovery Low Stress | No | No | No | No |
| phchp274v3 | BP | M | 48 | Caucasian | 9 | 36 | Discovery Low Stress | No | No | No | No |
| phchp274v4 | BP | M | 50 | Caucasian | 6 | 41 | Discovery Low Stress | No | No | No | No |
| phchp279v3 | SZ | M | 61 | African American | 4 | 43 | Discovery Low Stress | No | No | No | No |
| phchp282v5 | SZ | M | 59 | Caucasian | 0 | 27 | Discovery Low Stress | No | No | No | No |
| phchp285v1 | BP | F | 56 | Caucasian | 13 | 43 | Discovery Low Stress | No | No | No | No |
| phchp292v1 | BP | M | 42 | Caucasian | 0 | 36 | Discovery Low Stress | No | No | No | No |
| phchp302v1 | BP | M | 61 | Caucasian | 0 | 28 | Discovery Low Stress | No | No | No | No |
| phchp302v3 | BP | M | 61 | Caucasian | 26 | 40 | Discovery Low Stress | No | No | No | No |
| phchp329v3 | SZA | M | 51 | African American | 4 | 82 | Discovery Low Stress | No | No | No | No |
| phchp333v4 | PTSD | M | 39 | Caucasian | 12 | 68 | Discovery Low Stress | No | No | No | No |
| phchp340v3 | MDD | F | 52 | Caucasian | 31 | 63 | Discovery Low Stress | No | No | No | No |
| phchp351v1 | MDD | M | 44 | Caucasian | 2 | 73 | Discovery Low Stress | No | No | No | No |
| phchp361v3 | PTSD | F | 60 | African American | 0 | 71 | Discovery Low Stress | No | No | No | No |
| phchp362v2 | MDD | M | 54 | Caucasian | 14 | 77 | Discovery Low Stress | No | No | No | No |
| phchp109v1 | BP | M | 22 | Caucasian | 84 | 49 | Discovery High Stress | No | No | No | No |
| phchp132v6 | BP | M | 55 | Caucasian | 74 | 35 | Discovery High Stress | No | No | No | No |
| phchp134v2 | BP | M | 59 | Caucasian | 74 | 35 | Discovery High Stress | No | No | No | No |
| phchp151v1 | SZ | M | 24 | Caucasian | 95 | 42 | Discovery High Stress | No | No | No | No |
| phchp153v1 | BP | M | 55 | Caucasian | 85 | 37 | Discovery High Stress | No | No | No | No |
| phchp153v2 | BP | M | 55 | Caucasian | 98 | 41 | Discovery High Stress | No | No | No | No |
| phchp153v4 | BP | M | 57 | Caucasian | 76 | 41 | Discovery High Stress | No | No | No | No |
| phchp154v2 | PSYCH | M | 51 | African American | 76 | 24 | Discovery High Stress | No | No | No | No |
| phchp157v2 | BP | M | 57 | African American | 82 | 44 | Discovery High Stress | No | No | No | No |
| phchp173v1 | MDD | M | 48 | Caucasian | 72 | 28 | Discovery High Stress | No | No | No | No |
| phchp193v1 | BP | M | 39 | Hispanic | 70 |  | Discovery High Stress | No | No | No | No |
| phchp196v1 | MDD | M | 56 | African American | 80 | 43 | Discovery High Stress | No | No | No | No |
| phchp204v2 | BP | F | 49 | Caucasian | 79 | 47 | Discovery High Stress | No | No | No | No |
| phchp209v3 | PTSD | M | 54 | African American | 76 | 42 | Discovery High Stress | No | No | No | No |
| phchp226v2 | MDD | M | 29 | Caucasian | 81 | 48 | Discovery High Stress | No | No | No | No |
| phchp226v3 | MDD | M | 30 | Caucasian | 82 | 40 | Discovery High Stress | No | No | No | No |
| phchp274v1 | BP | M | 48 | Caucasian | 82 | 44 | Discovery High Stress | No | No | No | No |
| phchp274v2 | BP | M | 48 | Caucasian | 93 | 48 | Discovery High Stress | No | No | No | No |
| phchp279v1 | SZ | M | 60 | African American | 88 | 45 | Discovery High Stress | No | No | No | No |
| phchp279v2 | SZ | M | 61 | African American | 89 | 45 | Discovery High Stress | No | No | No | No |
| phchp282v1 | SZ | M | 56 | Caucasian | 85 | 43 | Discovery High Stress | No | No | No | No |
| phchp285v3 | BP | F | 57 | Caucasian | 68 | 24 | Discovery High Stress | No | No | No | No |
| phchp292v2 | BP | M | 42 | Caucasian | 89 | 31 | Discovery High Stress | No | No | No | No |
| phchp302v5 | BP | M | 62 | Caucasian | 84 | 39 | Discovery High Stress | No | No | No | No |

| **C. Independent Test Cohorts (n=201) (505 visits)** | | | | | | | | | | | | | | |
| --- | --- | --- | --- | --- | --- | --- | --- | --- | --- | --- | --- | --- | --- | --- |
| **Participant ID visit Number** | **Diagnosis** | **Gender(M/F)** | **Age at testing (Years)** | **Ethnicity** | **Discovery Cohort** | **Validation cohort** | **Test Cohort Stress State (VAS)** | **VAS**  **(0-100)** | **Test Cohort First Year Hospitalizations with Stress** | **Number of hospitalizations for Stress in the first year** | **Test Cohort All Future Hospitalizations with Stress** | **Number of All Future Hosp with Stress** | **Stress Hospit. Frequency** | **Time to First Future Hospitalization with Stress (days)** |
| phchp079v4 | BP | M | 49 | Caucasian | No | No | Yes | 0 | Yes | 0 | Yes | 0 | 0 | 803 |
| phchp079v5 | BP | M | 50 | Caucasian | No | No | Yes | 0 | Yes | 0 | Yes | 0 | 0 | 668 |
| phchp079v6 | BP | M | 50 | Caucasian | No | No | Yes | 2 | Yes | 0 | Yes | 0 | 0 | 535 |
| phchp088v4 | BP | M | 49 | Caucasian | No | No | Yes | 3 | Yes | 1 | Yes | 3 | 0.002072 | 316 |
| phchp088v5 | BP | M | 50 | Caucasian | No | No | Yes | 50 | Yes | 1 | Yes | 2 | 0.00177 | 171 |
| phchp088v6 | BP | M | 51 | Caucasian | No | No | Yes | 14 | Yes | 0 | Yes | 1 | 0.001078 | 749 |
| phchp092v3 | BP | M | 46 | African American | No | No | Yes | 27 | Yes | 1 | Yes | 2 | 0.000711 | 80 |
| phchp093v2 | BP | M | 51 | Caucasian | No | No | Yes | 51 | Yes | 1 | Yes | 7 | 0.002548 | 82 |
| phchp093v3 | BP | M | 52 | Caucasian | No | No | Yes | 66 | Yes | 0 | Yes | 6 | 0.002257 | 471 |
| phchp095v2 | BP | M | 29 | Caucasian | No | No | Yes | 15 | Yes | 0 | Yes | 1 | 0.000363 | 2222 |
| phchp095v3 | BP | M | 29 | Caucasian | No | No | Yes | 30 | Yes | 0 | Yes | 1 | 0.000376 | 2124 |
| phchp099v1 | SZ | M | 49 | Caucasian | No | No | Yes | 69 | Yes | 0 | Yes | 0 | 0 | 2751 |
| phchp099v3 | SZ | M | 49 | Caucasian | No | No | Yes | 44 | Yes | 0 | Yes | 0 | 0 | 2550 |
| phchp100v1 | BP | M | 28 | Caucasian | No | No | Yes | 71 | Yes | 0 | Yes | 1 | 0.000368 | 2544 |
| phchp106v1 | BP | F | 28 | Mixed | No | No | Yes | 58 | Yes | 0 | Yes | 0 | 0 | 2640 |
| phchp106v2 | BP | F | 28 | Mixed | No | No | Yes | 43 | Yes | 0 | Yes | 0 | 0 | 2552 |
| phchp106v3 | BP | F | 29 | Mixed | No | No | Yes | 33 | Yes | 0 | Yes | 0 | 0 | 2463 |
| phchp108v1 | SZ | M | 42 | Caucasian | No | No | Yes | 22 | Yes | 0 | Yes | 0 | 0 | 2617 |
| phchp108v2 | SZ | M | 42 | Caucasian | No | No | Yes | 18 | Yes | 0 | Yes | 0 | 0 | 2526 |
| phchp108v3 | SZ | M | 43 | Caucasian | No | No | Yes | 13 | Yes | 0 | Yes | 0 | 0 | 2430 |
| phchp112v1 | BP | M | 46 | Caucasian/Native Australian | No | No | Yes | 49 | Yes | 0 | Yes | 0 | 0 | 1569 |
| phchp112v2 | BP | M | 46 | Caucasian | No | No | Yes | 29 | Yes | 0 | Yes | 0 | 0 | 1480 |
| phchp112v3 | BP | M | 47 | Caucasian | No | No | Yes | 2 | Yes | 0 | Yes | 0 | 0 | 1359 |
| phchp113v1 | BP | M | 37 | Caucasian | No | No | Yes | 45 | Yes | 0 | Yes | 0 | 0 | 2517 |
| phchp114v1 | SZA | M | 54 | African American | No | No | Yes | 47 | Yes | 0 | Yes | 1 | 0.000411 | 2163 |
| phchp117v1 | BP | M | 43 | Caucasian | No | No | Yes | 63 | Yes | 0 | Yes | 0 | 0 | 2589 |
| phchp117v2 | BP | M | 43 | Caucasian | No | No | Yes | 8 | Yes | 0 | Yes | 0 | 0 | 2505 |
| phchp117v3 | BP | M | 43 | Caucasian | No | No | Yes | 24 | Yes | 0 | Yes | 0 | 0 | 2414 |
| phchp120v1 | SZ | M | 51 | Caucasian | No | No | Yes | 49 | Yes | 0 | Yes | 0 | 0 | 2476 |
| phchp120v2 | SZ | M | 51 | Caucasian | No | No | Yes | 98 | Yes | 0 | Yes | 0 | 0 | 2392 |
| phchp120v3 | SZ | M | 51 | Caucasian | No | No | Yes | 94 | Yes | 0 | Yes | 0 | 0 | 2301 |
| phchp122v1 | BP | M | 51 | Caucasian | No | No | Yes | 57 | Yes | 1 | Yes | 2 | 0.000838 | 334 |
| phchp122v2 | BP | M | 51 | Caucasian | No | No | Yes | 24 | Yes | 1 | Yes | 2 | 0.000867 | 255 |
| phchp127v1 | SZA | F | 58 | Caucasian | No | No | Yes | 68 | Yes | 0 | Yes | 0 | 0 | 747 |
| phchp128v1 | BP | M | 45 | Caucasian | No | No | Yes | 6 | Yes | 0 | Yes | 0 | 0 | 2349 |
| phchp128v2 | BP | M | 45 | Caucasian | No | No | Yes | 46 | Yes | 0 | Yes | 0 | 0 | 2246 |
| phchp129v1 | SZA | M | 22 | Caucasian | No | No | Yes | 3 | Yes | 0 | Yes | 1 | 0.000421 | 1937 |
| phchp129v2 | SZA | M | 25 | Caucasian | No | No | Yes | 8 | Yes | 0 | Yes | 1 | 0.000934 | 635 |
| phchp129v3 | SZA | M | 27 | Caucasian | No | No | Yes | 0 | Yes | 0 | Yes | 0 | 0 | 434 |
| phchp131v1 | SZ | F | 54 | African American | No | No | Yes | 42 | Yes | 0 | Yes | 1 | 0.000419 | 902 |
| phchp131v2 | SZ | F | 55 | African American | No | No | Yes | 3 | Yes | 0 | Yes | 1 | 0.00044 | 788 |
| phchp131v3 | SZ | F | 56 | African American | No | No | Yes | 29 | Yes | 0 | Yes | 1 | 0.000459 | 698 |
| phchp136v1 | BP | M | 41 | Caucasian | No | No | Yes | 42 | Yes | 0 | Yes | 0 | 0 | 2359 |
| phchp136v2 | BP | M | 41 | Caucasian | No | No | Yes | 56 | Yes | 0 | Yes | 0 | 0 | 2259 |
| phchp136v3 | BP | M | 41 | Caucasian | No | No | Yes | 72 | Yes | 0 | Yes | 0 | 0 | 2166 |
| phchp139v1 | SZ | M | 24 | Caucasian | No | No | Yes | 53 | Yes | 0 | Yes | 0 | 0 | 2332 |
| phchp140v1 | BP | M | 38 | Caucasian | No | No | Yes | 55 | Yes | 0 | Yes | 0 | 0 | 2288 |
| phchp140v2 | BP | M | 38 | Caucasian | No | No | Yes | 54 | Yes | 0 | Yes | 0 | 0 | 2191 |
| phchp140v3 | BP | M | 38 | Caucasian | No | No | Yes | 48 | Yes | 0 | Yes | 0 | 0 | 2101 |
| phchp140v4 | BP | M | 40 | Caucasian | No | No | Yes | 1 | Yes | 0 | Yes | 0 | 0 | 1287 |
| phchp142v1 | BP | M | 55 | Caucasian | No | No | Yes | 3 | Yes | 0 | Yes | 0 | 0 | 2302 |
| phchp142v2 | BP | M | 55 | Caucasian | No | No | Yes | 4 | Yes | 0 | Yes | 0 | 0 | 2212 |
| phchp142v3 | BP | M | 55 | Caucasian | No | No | Yes | 5 | Yes | 0 | Yes | 0 | 0 | 2125 |
| phchp147v1 | BP | M | 38 | Caucasian | No | No | Yes | 31 | Yes | 0 | Yes | 0 | 0 | 2231 |
| phchp147v2 | BP | M | 38 | Caucasian | No | No | Yes | 58 | Yes | 0 | Yes | 0 | 0 | 2141 |
| phchp147v3 | BP | M | 38 | Caucasian | No | No | Yes | 29 | Yes | 0 | Yes | 0 | 0 | 2054 |
| phchp148v1 | SZ | M | 25 | Caucasian | No | No | Yes | 14 | Yes | 0 | Yes | 0 | 0 | 2168 |
| phchp149v1 | MOOD | M | 45 | Caucasian | No | No | Yes | 13 | Yes | 0 | Yes | 0 | 0 | 1355 |
| phchp149v2 | MOOD | M | 45 | Caucasian | No | No | Yes | 48 | Yes | 0 | Yes | 0 | 0 | 1264 |
| phchp149v3 | MOOD | M | 46 | Caucasian | No | No | Yes | 45 | Yes | 0 | Yes | 0 | 0 | 1129 |
| phchp152v1 | BP | M | 45 | Caucasian | No | No | Yes | 48 | Yes | 1 | Yes | 1 | 0.000437 | 7 |
| phchp156v1 | BP | F | 35 | Caucasian | No | No | Yes | 56 | Yes | 0 | Yes | 0 | 0 | 2142 |
| phchp158v1 | BP | M | 23 | African American | No | No | Yes | 100 | Yes | 0 | Yes | 1 | 0.000462 | 1111 |
| phchp161v1 | MDD | M | 54 | African American | No | No | Yes | 57 | Yes | 0 | Yes | 1 | 0.000464 | 1575 |
| phchp161v2 | MDD | M | 54 | African American | No | No | Yes | 4 | Yes | 0 | Yes | 1 | 0.000487 | 1477 |
| phchp161v3 | MDD | M | 54 | African American | No | No | Yes | 5 | Yes | 0 | Yes | 1 | 0.000501 | 1417 |
| phchp168v1 | MDD | M | 48 | African American | No | No | Yes | 58 | Yes | 0 | Yes | 0 | 0 | 2153 |
| phchp168v2 | MDD | M | 48 | African American | No | No | Yes | 63 | Yes | 0 | Yes | 0 | 0 | 2062 |
| phchp168v3 | MDD | M | 49 | African American | No | No | Yes | 17 | Yes | 0 | Yes | 0 | 0 | 1970 |
| phchp169v1 | SZA | M | 50 | African American | No | No | Yes | 0 | Yes | 0 | Yes | 0 | 0 | 2003 |
| phchp171v1 | BP | M | 36 | Caucasian | No | No | Yes | 60 | Yes | 0 | Yes | 0 | 0 | 2402 |
| phchp171v2 | BP | M | 36 | Caucasian | No | No | Yes | 73 | Yes | 0 | Yes | 0 | 0 | 2276 |
| phchp174v1 | MDD | M | 54 | Caucasian | No | No | Yes | 65 | Yes | 0 | Yes | 1 | 0.001175 | 604 |
| phchp175v1 | SZA | M | 42 | Caucasian | No | No | Yes | 0 | Yes | 1 | Yes | 1 | 0.00041 | 181 |
| phchp177v1 | SZ | F | 39 | Caucasian | No | No | Yes | 13 | Yes | 0 | Yes | 0 | 0 | 2339 |
| phchp177v2 | SZ | F | 39 | Caucasian | No | No | Yes | 8 | Yes | 0 | Yes | 0 | 0 | 2230 |
| phchp178v1 | BP | M | 49 | Caucasian | No | No | Yes | 45 | Yes | 0 | Yes | 1 | 0.000421 | 2107 |
| phchp183v1 | BP | M | 48 | Caucasian | No | No | Yes | 16 | Yes | 0 | Yes | 0 | 0 | 2374 |
| phchp183v2 | BP | M | 48 | Caucasian | No | No | Yes | 69 | Yes | 0 | Yes | 0 | 0 | 2278 |
| phchp185v1 | SZA | M | 51 | African American | No | No | Yes | 40 | Yes | 0 | Yes | 0 | 0 | 2352 |
| phchp185v2 | SZA | M | 51 | African American | No | No | Yes | 9 | Yes | 0 | Yes | 0 | 0 | 2258 |
| phchp185v3 | SZA | M | 52 | African American | No | No | Yes | 8 | Yes | 0 | Yes | 0 | 0 | 2121 |
| phchp186v1 | BP | M | 43 | Caucasian | No | No | Yes | 8 | Yes | 0 | Yes | 0 | 0 | 2310 |
| phchp186v2 | BP | M | 44 | Caucasian | No | No | Yes | 5 | Yes | 0 | Yes | 0 | 0 | 2206 |
| phchp186v3 | BP | M | 44 | Caucasian | No | No | Yes | 2 | Yes | 0 | Yes | 0 | 0 | 2128 |
| phchp186v4 | BP | M | 46 | Caucasian | No | No | Yes | 62 | Yes | 0 | Yes | 0 | 0 | 1319 |
| phchp186v5 | BP | M | 48 | Caucasian | No | No | Yes | 1 | Yes | 0 | Yes | 0 | 0 | 469 |
| phchp187v1 | SZ | M | 49 | African American | No | No | Yes | 7 | Yes | 2 | Yes | 7 | 0.002961 | 14 |
| phchp187v2 | SZ | M | 49 | African American | No | No | Yes | 36 | Yes | 1 | Yes | 6 | 0.002673 | 181 |
| phchp188v3 | PSYCH | M | 54 | African American | No | No | Yes | 11 | Yes | 0 | Yes | 1 | 0.000483 | 650 |
| phchp190v1 | BP | M | 49 | Caucasian | No | No | Yes | 52 | Yes | 0 | Yes | 2 | 0.000853 | 1845 |
| phchp190v2 | BP | M | 49 | Caucasian | No | No | Yes | 48 | Yes | 0 | Yes | 2 | 0.00089 | 1749 |
| phchp190v3 | BP | M | 50 | Caucasian | No | No | Yes | 37 | Yes | 0 | Yes | 2 | 0.000935 | 1640 |
| phchp190v4 | BP | M | 54 | Caucasian | No | No | Yes | 10 | Yes | 1 | Yes | 1 | 0.002012 | 90 |
| phchp192v1 | SZA | M | 55 | African American | No | No | Yes | 55 | Yes | 0 | Yes | 0 | 0 | 2276 |
| phchp194v1 | MDD | M | 47 | Caucasian | No | No | Yes | 11 | Yes | 0 | Yes | 0 | 0 | 2227 |
| phchp194v2 | MDD | M | 47 | Caucasian | No | No | Yes | 38 | Yes | 0 | Yes | 0 | 0 | 2138 |
| phchp194v3 | MDD | M | 47 | Caucasian | No | No | Yes | 21 | Yes | 0 | Yes | 0 | 0 | 2046 |
| phchp195v1 | SZ | M | 52 | Caucasian | No | No | Yes | 47 | Yes | 0 | Yes | 0 | 0 | 1241 |
| phchp195v2 | SZ | M | 53 | Caucasian | No | No | Yes | 64 | Yes | 0 | Yes | 0 | 0 | 1150 |
| phchp195v3 | SZ | M | 53 | Caucasian | No | No | Yes | 45 | Yes | 0 | Yes | 0 | 0 | 1061 |
| phchp197v1 | SZ | M | 56 | Caucasian | No | No | Yes | 53 | Yes | 0 | Yes | 0 | 0 | 2247 |
| phchp199v1 | SZ | M | 49 | African American | No | No | Yes | 7 | Yes | 0 | Yes | 0 | 0 | 2259 |
| phchp199v2 | SZ | M | 49 | African American | No | No | Yes | 3 | Yes | 0 | Yes | 0 | 0 | 2161 |
| phchp199v3 | SZ | M | 50 | African American | No | No | Yes | 4 | Yes | 0 | Yes | 0 | 0 | 2047 |
| phchp200v1 | MDD | M | 56 | Caucasian | No | No | Yes | 49 | Yes | 0 | Yes | 0 | 0 | 2092 |
| phchp207v1 | SZ | M | 48 | African American | No | No | Yes | 33 | Yes | 0 | Yes | 0 | 0 | 1991 |
| phchp210v1 | BP | M | 43 | Caucasian | No | No | Yes | 13 | Yes | 0 | Yes | 0 | 0 | 2141 |
| phchp210v2 | BP | M | 43 | Caucasian | No | No | Yes | 8 | Yes | 0 | Yes | 0 | 0 | 2038 |
| phchp210v3 | BP | M | 44 | Caucasian | No | No | Yes | 26 | Yes | 0 | Yes | 0 | 0 | 1833 |
| phchp212v1 | MDD | M | 56 | African American | No | No | Yes | 13 | Yes | 0 | Yes | 0 | 0 | 1876 |
| phchp217v1 | PTSD | M | 36 | African American | No | No | Yes | 45 | Yes | 0 | Yes | 0 | 0 | 1783 |
| phchp221v1 | MDD | M | 51 | African American | No | No | Yes | 27 | Yes | 0 | Yes | 0 | 0 | 1799 |
| phchp221v2 | MDD | M | 51 | African American | No | No | Yes | 38 | Yes | 0 | Yes | 0 | 0 | 1707 |
| phchp221v3 | MDD | M | 52 | African American | No | No | Yes | 0 | Yes | 0 | Yes | 0 | 0 | 1583 |
| phchp224v1 | BP | M | 59 | Caucasian | No | No | Yes | 92 | Yes | 2 | Yes | 3 | 0.001458 | 307 |
| phchp227v1 | MDD | M | 55 | Caucasian | No | No | Yes | 17 | Yes | 0 | Yes | 0 | 0 | 2046 |
| phchp227v2 | MDD | M | 55 | Caucasian | No | No | Yes | 4 | Yes | 0 | Yes | 0 | 0 | 1959 |
| phchp227v3 | MDD | M | 55 | Caucasian | No | No | Yes | 1 | Yes | 0 | Yes | 0 | 0 | 1856 |
| phchp228v1 | PTSD | M | 43 | African American | No | No | Yes | 35 | Yes | 0 | Yes | 0 | 0 | 2057 |
| phchp229v1 | PTSD | M | 55 | African American | No | No | Yes | 58 | Yes | 0 | Yes | 0 | 0 | 2015 |
| phchp235v1 | MDD | M | 54 | African American | No | No | Yes | 40 | Yes | 0 | Yes | 0 | 0 | 2040 |
| phchp235v2 | MDD | M | 55 | African American | No | No | Yes | 46 | Yes | 0 | Yes | 0 | 0 | 1948 |
| phchp235v3 | MDD | M | 55 | African American | No | No | Yes | 1 | Yes | 0 | Yes | 0 | 0 | 1829 |
| phchp236v1 | MDD | M | 51 | Caucasian | No | No | Yes | 68 | Yes | 0 | Yes | 0 | 0 | 2031 |
| phchp236v2 | MDD | M | 51 | Caucasian | No | No | Yes | 98 | Yes | 0 | Yes | 0 | 0 | 1939 |
| phchp236v3 | MDD | M | 54 | Caucasian | No | No | Yes | 49 | Yes | 0 | Yes | 0 | 0 | 1173 |
| phchp240v1 | MDD | F | 55 | Caucasian | No | No | Yes | 53 | Yes | 0 | Yes | 0 | 0 | 1799 |
| phchp240v2 | MDD | F | 55 | Caucasian | No | No | Yes | 5 | Yes | 0 | Yes | 0 | 0 | 1576 |
| phchp240v3 | MDD | F | 56 | Caucasian | No | No | Yes | 13 | Yes | 0 | Yes | 0 | 0 | 1445 |
| phchp242v1 | MDD | M | 55 | African American | No | No | Yes | 37 | Yes | 0 | Yes | 0 | 0 | 1912 |
| phchp247v1 | MDD | M | 55 | African American | No | No | Yes | 20 | Yes | 0 | Yes | 0 | 0 | 2024 |
| phchp253v1 | BP | M | 25 | Caucasian | No | No | Yes | 2 | Yes | 0 | Yes | 0 | 0 | 1993 |
| phchp253v2 | BP | M | 26 | Caucasian | No | No | Yes | 51 | Yes | 0 | Yes | 0 | 0 | 1686 |
| phchp253v3 | BP | M | 26 | Caucasian | No | No | Yes | 0 | Yes | 0 | Yes | 0 | 0 | 1530 |
| phchp254v1 | MDD | F | 49 | Caucasian | No | No | Yes | 4 | Yes | 0 | Yes | 0 | 0 | 1644 |
| phchp254v2 | MDD | F | 49 | Caucasian | No | No | Yes | 18 | Yes | 0 | Yes | 0 | 0 | 1420 |
| phchp254v3 | MDD | F | 50 | Caucasian | No | No | Yes | 28 | Yes | 0 | Yes | 0 | 0 | 1273 |
| phchp259v1 | MDD | M | 56 | Caucasian | No | No | Yes | 43 | Yes | 0 | Yes | 0 | 0 | 702 |
| phchp265v1 | PTSD | M | 43 | Caucasian | No | No | Yes | 84 | Yes | 0 | Yes | 0 | 0 | 1478 |
| phchp270v3 | BP | M | 41 | Caucasian | No | No | Yes | 9 | Yes | 0 | Yes | 0 | 0 | 1586 |
| phchp270v4 | BP | M | 41 | Caucasian | No | No | Yes | 2 | Yes | 0 | Yes | 0 | 0 | 1476 |
| phchp270v5 | BP | M | 42 | Caucasian | No | No | Yes | 10 | Yes | 0 | Yes | 0 | 0 | 1259 |
| phchp270v6 | BP | M | 44 | Caucasian | No | No | Yes | 95 | Yes | 0 | Yes | 0 | 0 | 539 |
| phchp273v1 | BP | M | 27 | Caucasian | No | No | Yes | 46 | Yes | 0 | Yes | 0 | 0 | 1881 |
| phchp273v2 | BP | M | 28 | Caucasian | No | No | Yes | 57 | Yes | 0 | Yes | 0 | 0 | 1790 |
| phchp277v1 | SZ | M | 49 | Caucasian | No | No | Yes | 48 | Yes | 0 | Yes | 0 | 0 | 1876 |
| phchp277v2 | SZ | M | 50 | Caucasian | No | No | Yes | 55 | Yes | 0 | Yes | 0 | 0 | 1783 |
| phchp277v3 | SZ | M | 50 | Caucasian | No | No | Yes | 43 | Yes | 0 | Yes | 0 | 0 | 1690 |
| phchp277v4 | SZ | M | 52 | Caucasian | No | No | Yes | 48 | Yes | 0 | Yes | 0 | 0 | 836 |
| phchp277v5 | SZ | M | 52 | Caucasian | No | No | Yes | 47 | Yes | 0 | Yes | 0 | 0 | 731 |
| phchp283v1 | SZ | M | 51 | Caucasian | No | No | Yes | 20 | Yes | 0 | Yes | 0 | 0 | 1666 |
| phchp290v1 | BP | M | 55 | Caucasian | No | No | Yes | 15 | Yes | 0 | Yes | 0 | 0 | 1608 |
| phchp290v2 | BP | M | 55 | Caucasian | No | No | Yes | 61 | Yes | 0 | Yes | 0 | 0 | 1473 |
| phchp290v3 | BP | M | 55 | Caucasian | No | No | Yes | 7 | Yes | 0 | Yes | 0 | 0 | 1376 |
| phchp293v1 | BP | M | 43 | Caucasian | No | No | Yes | 62 | Yes | 0 | Yes | 0 | 0 | 1746 |
| phchp293v2 | BP | M | 44 | Caucasian | No | No | Yes | 45 | Yes | 0 | Yes | 0 | 0 | 1644 |
| phchp295v1 | SZ | M | 52 | African American | No | No | Yes | 0 | Yes | 0 | Yes | 0 | 0 | 375 |
| phchp297v1 | SZA | M | 54 | African American | No | No | Yes | 0 | Yes | 0 | Yes | 0 | 0 | 1422 |
| phchp297v2 | SZA | M | 55 | African American | No | No | Yes | 0 | Yes | 0 | Yes | 0 | 0 | 1314 |
| phchp297v3 | SZA | M | 55 | African American | No | No | Yes | 0 | Yes | 0 | Yes | 0 | 0 | 1204 |
| phchp300v1 | BP | M | 47 | Caucasian | No | No | Yes | 42 | Yes | 0 | Yes | 0 | 0 | 1393 |
| phchp300v2 | BP | M | 47 | Caucasian | No | No | Yes | 10 | Yes | 0 | Yes | 0 | 0 | 1300 |
| phchp300v3 | BP | M | 48 | Caucasian | No | No | Yes | 11 | Yes | 0 | Yes | 0 | 0 | 1111 |
| phchp300v4 | BP | M | 49 | Caucasian | No | No | Yes | 8 | Yes | 0 | Yes | 0 | 0 | 630 |
| phchp308v1 | SZA | M | 47 | African American | No | No | Yes | 38 | Yes | 0 | Yes | 0 | 0 | 1437 |
| phchp308v2 | SZA | M | 47 | African American | No | No | Yes | 5 | Yes | 0 | Yes | 0 | 0 | 1200 |
| phchp308v3 | SZA | M | 48 | African American | No | No | Yes | 5 | Yes | 0 | Yes | 0 | 0 | 842 |
| phchp308v4 | SZA | M | 49 | African American | No | No | Yes | 2 | Yes | 0 | Yes | 0 | 0 | 395 |
| phchp309v1 | PTSD | F | 27 | Caucasian | No | No | Yes | 93 | Yes | 0 | Yes | 0 | 0 | 1423 |
| phchp309v2 | PTSD | F | 28 | Caucasian | No | No | Yes | 98 | Yes | 0 | Yes | 0 | 0 | 1235 |
| phchp309v3 | PTSD | F | 28 | Caucasian | No | No | Yes | 99 | Yes | 0 | Yes | 0 | 0 | 1093 |
| phchp314v1 | BP | M | 54 | Caucasian | No | No | Yes | 34 | Yes | 0 | Yes | 0 | 0 | 1217 |
| phchp314v2 | BP | M | 54 | Caucasian | No | No | Yes | 53 | Yes | 0 | Yes | 0 | 0 | 1113 |
| phchp314v3 | BP | M | 54 | Caucasian | No | No | Yes | 17 | Yes | 0 | Yes | 0 | 0 | 1022 |
| phchp322v1 | BP | M | 26 | Caucasian | No | No | Yes | 51 | Yes | 0 | Yes | 0 | 0 | 1137 |
| phchp324v1 | MDD | M | 33 | African American | No | No | Yes | 17 | Yes | 0 | Yes | 0 | 0 | 840 |
| phchp327v1 | MDD | M | 42 | Caucasian | No | No | Yes | 100 | Yes | 0 | Yes | 0 | 0 | 1197 |
| phchp330v1 | BP | F | 45 | Caucasian | No | No | Yes | 100 | Yes | 0 | Yes | 0 | 0 | 1215 |
| phchp335v1 | MDD | M | 25 | Caucasian | No | No | Yes | 92 | Yes | 0 | Yes | 0 | 0 | 1133 |
| phchp338v1 | BP | F | 51 | Caucasian | No | No | Yes | 50 | Yes | 0 | Yes | 0 | 0 | 1102 |
| phchp338v2 | BP | F | 51 | Caucasian | No | No | Yes | 14 | Yes | 0 | Yes | 0 | 0 | 977 |
| phchp338v3 | BP | F | 51 | Caucasian | No | No | Yes | 1 | Yes | 0 | Yes | 0 | 0 | 878 |
| phchp338v4 | BP | F | 52 | Caucasian | No | No | Yes | 2 | Yes | 0 | Yes | 0 | 0 | 774 |
| phchp338v5 | BP | F | 52 | Caucasian | No | No | Yes | 3 | Yes | 0 | Yes | 0 | 0 | 647 |
| phchp343v1 | MDD | M | 52 | Caucasian | No | No | Yes | 11 | Yes | 0 | Yes | 0 | 0 | 917 |
| phchp343v2 | MDD | M | 52 | Caucasian | No | No | Yes | 5 | Yes | 0 | Yes | 0 | 0 | 825 |
| phchp343v3 | MDD | M | 53 | Caucasian | No | No | Yes | 15 | Yes | 0 | Yes | 0 | 0 | 659 |
| phchp344v1 | PTSD | M | 33 | Caucasian | No | No | Yes | 2 | Yes | 0 | Yes | 0 | 0 | 1048 |
| phchp346v1 | PTSD | F | 36 | African American | No | No | Yes | 50 | Yes | 0 | Yes | 0 | 0 | 1035 |
| phchp346v2 | PTSD | F | 37 | African American | No | No | Yes | 43 | Yes | 0 | Yes | 0 | 0 | 921 |
| phchp346v3 | PTSD | F | 37 | African American | No | No | Yes | 62 | Yes | 0 | Yes | 0 | 0 | 739 |
| phchp348v1 | BP | M | 52 | Caucasian | No | No | Yes | 20 | Yes | 0 | Yes | 0 | 0 | 904 |
| phchp349v2 | PSYCH | M | 58 | Caucasian | No | No | Yes | 70 | Yes | 0 | Yes | 0 | 0 | 897 |
| phchp350v1 | BP | M | 48 | Caucasian | No | No | Yes | 12 | Yes | 1 | Yes | 1 | 0.001133 | 96 |
| phchp350v2 | BP | M | 48 | Caucasian | No | No | Yes | 14 | Yes | 0 | Yes | 0 | 0 | 779 |
| phchp350v3 | BP | M | 49 | Caucasian | No | No | Yes | 2 | Yes | 0 | Yes | 0 | 0 | 416 |
| phchp352v1 | MDD | M | 24 | African American | No | No | Yes | 100 | Yes | 1 | Yes | 2 | 0.002336 | 250 |
| phchp353v1 | MDD | F | 45 | Caucasian | No | No | Yes | 8 | Yes | 0 | Yes | 0 | 0 | 836 |
| phchp355v1 | MDD | F | 50 | Caucasian | No | No | Yes | 30 | Yes | 0 | Yes | 0 | 0 | 867 |
| phchp355v2 | MDD | F | 50 | Caucasian | No | No | Yes | 5 | Yes | 0 | Yes | 0 | 0 | 727 |
| phchp356v1 | BP | M | 40 | Caucasian | No | No | Yes | 23 | Yes | 0 | Yes | 0 | 0 | 812 |
| phchp357v1 | BP | M | 45 | Caucasian | No | No | Yes | 23 | Yes | 0 | Yes | 0 | 0 | 799 |
| phchp357v2 | BP | M | 45 | Caucasian | No | No | Yes | 34 | Yes | 0 | Yes | 0 | 0 | 705 |
| phchp357v3 | BP | M | 45 | Caucasian | No | No | Yes | 12 | Yes | 0 | Yes | 0 | 0 | 566 |
| phchp357v4 | BP | M | 45 | Caucasian | No | No | Yes | 7 | Yes | 0 | Yes | 0 | 0 | 474 |
| phchp358v1 | PTSD | M | 52 | Hispanic | No | No | Yes | 13 | Yes | 0 | Yes | 0 | 0 | 657 |
| phchp358v2 | PTSD | M | 52 | Hispanic | No | No | Yes | 2 | Yes | 0 | Yes | 0 | 0 | 562 |
| phchp358v3 | PTSD | M | 53 | Hispanic | No | No | Yes | 3 | Yes | 0 | Yes | 0 | 0 | 425 |
| phchp360v1 | BP | F | 56 | Caucasian | No | No | Yes | 49 | Yes | 0 | Yes | 0 | 0 | 708 |
| phchp367v1 | BP | M | 48 | Caucasian | No | No | Yes | 12 | Yes | 0 | Yes | 1 | 0.00188 | 467 |
| phchp367v2 | BP | M | 49 | Caucasian | No | No | Yes | 49 | Yes | 1 | Yes | 1 | 0.002488 | 337 |
| phchp003v1 | SZ | M | 50 | African American | No | No | No |  | Yes | 0 | Yes | 2 | 0.000486 | 2345 |
| phchp003v2 | SZ | M | 50 | African American | No | No | No |  | Yes | 0 | Yes | 2 | 0.000507 | 2177 |
| phchp003v3 | SZ | M | 50 | African American | No | No | No |  | Yes | 0 | Yes | 2 | 0.000519 | 2086 |
| phchp003v4 | SZ | M | 57 | African American | No | No | No | 41 | Yes | 0 | Yes | 1 | 0.000895 | 553 |
| phchp004v1 | SZA | M | 55 | African American | No | No | No |  | Yes | 0 | Yes | 0 | 0 | 4086 |
| phchp005v1 | SZA | M | 45 | Caucasian | No | No | No |  | Yes | 0 | Yes | 7 | 0.001733 | 2593 |
| phchp005v2 | SZA | M | 45 | Caucasian | No | No | No |  | Yes | 0 | Yes | 7 | 0.001791 | 2462 |
| phchp005v3 | SZA | M | 45 | Caucasian | No | No | No |  | Yes | 0 | Yes | 7 | 0.001835 | 2367 |
| phchp006v1 | SZA | M | 52 | African American | No | No | No |  | Yes | 0 | Yes | 2 | 0.000498 | 2720 |
| phchp006v2 | SZA | M | 52 | African American | No | No | No |  | Yes | 0 | Yes | 2 | 0.000509 | 2635 |
| phchp008v1 | SZ | M | 47 | African American | No | No | No |  | Yes | 0 | Yes | 0 | 0 | 1902 |
| phchp009v1 | SZ | M | 55 | African American | No | No | No |  | Yes | 0 | Yes | 0 | 0 | 2375 |
| phchp009v3 | SZ | M | 56 | African American | No | No | No |  | Yes | 0 | Yes | 0 | 0 | 2195 |
| phchp010v1 | SZA | M | 45 | Caucasian | No | No | No |  | Yes | 0 | Yes | 0 | 0 | 3993 |
| phchp010v2 | SZA | M | 45 | Caucasian | No | No | No |  | Yes | 0 | Yes | 0 | 0 | 3909 |
| phchp010v3 | SZA | M | 45 | Caucasian | No | No | No |  | Yes | 0 | Yes | 0 | 0 | 3818 |
| phchp012v1 | SZA | M | 55 | Caucasian | No | No | No |  | Yes | 0 | Yes | 0 | 0 | 3972 |
| phchp012v2 | SZA | M | 55 | Caucasian | No | No | No |  | Yes | 0 | Yes | 0 | 0 | 3897 |
| phchp012v3 | SZA | M | 55 | Caucasian | No | No | No |  | Yes | 0 | Yes | 0 | 0 | 3806 |
| phchp013v1 | SZA | M | 53 | African American | No | No | No |  | Yes | 0 | Yes | 0 | 0 | 3962 |
| phchp013v3 | SZA | M | 54 | African American | No | No | No |  | Yes | 0 | Yes | 0 | 0 | 3780 |
| phchp014v1 | PSYCH | M | 55 | African American | No | No | No |  | Yes | 0 | Yes | 1 | 0.000253 | 2949 |
| phchp016v1 | SZ | M | 54 | African American | No | No | No |  | Yes | 0 | Yes | 0 | 0 | 2022 |
| phchp016v2 | SZ | M | 54 | African American | No | No | No |  | Yes | 0 | Yes | 0 | 0 | 1924 |
| phchp016v3 | SZ | M | 54 | African American | No | No | No |  | Yes | 0 | Yes | 0 | 0 | 1831 |
| phchp017v2 | SZA | M | 53 | African American | No | No | No |  | Yes | 0 | Yes | 0 | 0 | 556 |
| phchp017v3 | SZA | M | 54 | African American | No | No | No |  | Yes | 0 | Yes | 0 | 0 | 385 |
| phchp019v1 | SZ | M | 50 | African American | No | No | No |  | Yes | 0 | Yes | 1 | 0.000256 | 1511 |
| phchp019v2 | SZ | M | 51 | African American | No | No | No |  | Yes | 0 | Yes | 1 | 0.000264 | 1397 |
| phchp019v3 | SZ | M | 51 | African American | No | No | No |  | Yes | 0 | Yes | 1 | 0.000274 | 1250 |
| phchp021v1 | SZA | M | 48 | Hispanic | No | No | No |  | Yes | 0 | Yes | 0 | 0 | 3895 |
| phchp021v2 | SZA | M | 49 | Hispanic | No | No | No |  | Yes | 0 | Yes | 0 | 0 | 3808 |
| phchp021v3 | SZA | M | 49 | Hispanic | No | No | No |  | Yes | 0 | Yes | 0 | 0 | 3694 |
| phchp022v1 | SZ | M | 48 | Caucasian | No | No | No |  | Yes | 0 | Yes | 0 | 0 | 3864 |
| phchp022v2 | SZ | M | 48 | Caucasian | No | No | No |  | Yes | 0 | Yes | 0 | 0 | 3761 |
| phchp024v1 | SZA | M | 49 | African American | No | No | No |  | Yes | 1 | Yes | 7 | 0.001804 | 358 |
| phchp025v1 | SZ | M | 42 | Caucasian | No | No | No |  | Yes | 0 | Yes | 0 | 0 | 3858 |
| phchp026v1 | SZA | M | 49 | African American | No | No | No |  | Yes | 0 | Yes | 0 | 0 | 3892 |
| phchp026v2 | SZA | M | 49 | African American | No | No | No |  | Yes | 0 | Yes | 0 | 0 | 3798 |
| phchp026v3 | SZA | M | 49 | African American | No | No | No |  | Yes | 0 | Yes | 0 | 0 | 3693 |
| phchp027v1 | SZA | M | 40 | Caucasian | No | No | No |  | Yes | 1 | Yes | 2 | 0.000518 | 4 |
| phchp030v1 | BP | M | 49 | Caucasian | No | No | No |  | Yes | 1 | Yes | 4 | 0.00104 | 94 |
| phchp030v3 | BP | M | 49 | Caucasian | No | No | No |  | Yes | 0 | Yes | 3 | 0.000829 | 456 |
| phchp031v1 | BP | M | 51 | Caucasian | No | No | No |  | Yes | 0 | Yes | 1 | 0.000259 | 1674 |
| phchp031v2 | BP | M | 51 | Caucasian | No | No | No |  | Yes | 0 | Yes | 1 | 0.000265 | 1576 |
| phchp031v3 | BP | M | 52 | Caucasian | No | No | No |  | Yes | 0 | Yes | 1 | 0.000273 | 1465 |
| phchp033v1 | SZA | M | 48 | Caucasian | No | No | No |  | Yes | 0 | Yes | 25 | 0.006879 | 865 |
| phchp038v1 | SZA | M | 58 | African American | No | No | No |  | Yes | 0 | Yes | 1 | 0.000375 | 880 |
| phchp038v2 | SZA | M | 58 | African American | No | No | No |  | Yes | 0 | Yes | 1 | 0.000391 | 773 |
| phchp039v1 | BP | M | 52 | Caucasian | No | No | No |  | Yes | 0 | Yes | 0 | 0 | 3801 |
| phchp039v3 | BP | M | 52 | Caucasian | No | No | No |  | Yes | 0 | Yes | 0 | 0 | 3563 |
| phchp040v1 | SZA | M | 50 | Caucasian | No | No | No |  | Yes | 0 | Yes | 0 | 0 | 2110 |
| phchp040v2 | SZA | M | 50 | Caucasian | No | No | No |  | Yes | 0 | Yes | 0 | 0 | 2026 |
| phchp040v3 | SZA | M | 50 | Caucasian | No | No | No |  | Yes | 0 | Yes | 0 | 0 | 1921 |
| phchp041v1 | SZ | M | 62 | African American | No | No | No |  | Yes | 1 | Yes | 1 | 0.000268 | 14 |
| phchp042v1 | SZA | M | 43 | Caucasian | No | No | No |  | Yes | 0 | Yes | 0 | 0 | 3713 |
| phchp042v2 | SZA | M | 43 | Caucasian | No | No | No |  | Yes | 0 | Yes | 0 | 0 | 3615 |
| phchp042v3 | SZA | M | 44 | Caucasian | No | No | No |  | Yes | 0 | Yes | 0 | 0 | 3530 |
| phchp046v1 | SZA | M | 45 | Caucasian | No | No | No |  | Yes | 0 | Yes | 2 | 0.000547 | 3178 |
| phchp046v2 | SZA | M | 45 | Caucasian | No | No | No |  | Yes | 0 | Yes | 2 | 0.000558 | 3106 |
| phchp046v3 | SZA | M | 45 | Caucasian | No | No | No |  | Yes | 0 | Yes | 2 | 0.000573 | 3015 |
| phchp047v1 | SZA | M | 57 | African American | No | No | No |  | Yes | 0 | Yes | 1 | 0.000355 | 1463 |
| phchp047v2 | SZA | M | 57 | African American | No | No | No |  | Yes | 0 | Yes | 1 | 0.000367 | 1371 |
| phchp047v3 | SZA | M | 58 | African American | No | No | No |  | Yes | 0 | Yes | 1 | 0.00038 | 1279 |
| phchp048v1 | SZA | M | 56 | African American | No | No | No |  | Yes | 0 | Yes | 0 | 0 | 1925 |
| phchp048v2 | SZA | M | 57 | African American | No | No | No |  | Yes | 0 | Yes | 0 | 0 | 1837 |
| phchp048v3 | SZA | M | 57 | African American | No | No | No |  | Yes | 0 | Yes | 0 | 0 | 1732 |
| phchp049v1 | SZA | M | 46 | Caucasian | No | No | No |  | Yes | 0 | Yes | 0 | 0 | 3494 |
| phchp049v2 | SZA | M | 47 | Caucasian | No | No | No |  | Yes | 0 | Yes | 0 | 0 | 3413 |
| phchp051v1 | SZA | M | 52 | Caucasian | No | No | No |  | Yes | 0 | Yes | 0 | 0 | 3659 |
| phchp053v1 | BP | M | 58 | Caucasian | No | No | No |  | Yes | 0 | Yes | 2 | 0.000555 | 686 |
| phchp053v2 | BP | M | 58 | Caucasian | No | No | No |  | Yes | 0 | Yes | 2 | 0.000571 | 585 |
| phchp053v3 | BP | M | 58 | Caucasian | No | No | No |  | Yes | 0 | Yes | 2 | 0.000589 | 483 |
| phchp055v1 | BP | F | 46 | Caucasian | No | No | No |  | Yes | 1 | Yes | 1 | 0.000282 | 59 |
| phchp055v2 | BP | F | 46 | Caucasian | No | No | No |  | Yes | 0 | Yes | 0 | 0 | 3433 |
| phchp055v3 | BP | F | 46 | Caucasian | No | No | No |  | Yes | 0 | Yes | 0 | 0 | 3330 |
| phchp057v1 | SZA | M | 47 | Caucasian | No | No | No |  | Yes | 0 | Yes | 0 | 0 | 3576 |
| phchp058v1 | SZ | M | 56 | African American | No | No | No |  | Yes | 0 | Yes | 0 | 0 | 2593 |
| phchp058v2 | SZ | M | 56 | African American | No | No | No |  | Yes | 0 | Yes | 0 | 0 | 2501 |
| phchp058v3 | SZ | M | 56 | African American | No | No | No |  | Yes | 0 | Yes | 0 | 0 | 2395 |
| phchp061v1 | SZ | M | 49 | Caucasian | No | No | No |  | Yes | 0 | Yes | 0 | 0 | 3501 |
| phchp061v2 | SZ | M | 49 | Caucasian | No | No | No |  | Yes | 0 | Yes | 0 | 0 | 3415 |
| phchp061v3 | SZ | M | 50 | Caucasian | No | No | No |  | Yes | 0 | Yes | 0 | 0 | 3193 |
| phchp062v1 | SZ | M | 56 | Caucasian | No | No | No |  | Yes | 0 | Yes | 0 | 0 | 3554 |
| phchp062v2 | SZ | M | 56 | Caucasian | No | No | No |  | Yes | 0 | Yes | 0 | 0 | 3467 |
| phchp062v3 | SZ | M | 57 | Caucasian | No | No | No |  | Yes | 0 | Yes | 0 | 0 | 3372 |
| phchp067v1 | BP | M | 39 | Caucasian | No | No | No |  | Yes | 0 | Yes | 0 | 0 | 3459 |
| phchp067v3 | BP | M | 40 | Caucasian | No | No | No |  | Yes | 0 | Yes | 0 | 0 | 3249 |
| phchp068v1 | SZA | M | 57 | African American | No | No | No |  | Yes | 0 | Yes | 0 | 0 | 3481 |
| phchp068v2 | SZA | M | 57 | African American | No | No | No |  | Yes | 0 | Yes | 0 | 0 | 3350 |
| phchp068v3 | SZA | M | 57 | African American | No | No | No |  | Yes | 0 | Yes | 0 | 0 | 3255 |
| phchp069v1 | SZ | M | 47 | Caucasian | No | No | No |  | Yes | 0 | Yes | 0 | 0 | 3523 |
| phchp069v2 | SZ | M | 47 | Caucasian | No | No | No |  | Yes | 0 | Yes | 0 | 0 | 3414 |
| phchp069v3 | SZ | M | 48 | Caucasian | No | No | No |  | Yes | 0 | Yes | 0 | 0 | 3323 |
| phchp070v1 | SZ | M | 52 | African American | No | No | No |  | Yes | 0 | Yes | 0 | 0 | 3452 |
| phchp070v2 | SZ | M | 52 | African American | No | No | No |  | Yes | 0 | Yes | 0 | 0 | 3329 |
| phchp070v3 | SZ | M | 52 | African American | No | No | No |  | Yes | 0 | Yes | 0 | 0 | 3238 |
| phchp070v4 | SZ | M | 56 | African American | No | No | No | 1 | Yes | 0 | Yes | 0 | 0 | 1813 |
| phchp070v5 | SZ | M | 56 | African American | No | No | No | 1 | Yes | 0 | Yes | 0 | 0 | 1726 |
| phchp070v6 | SZ | M | 57 | African American | No | No | No | 0 | Yes | 0 | Yes | 0 | 0 | 1637 |
| phchp073v1 | SZA | M | 50 | Caucasian | No | No | No |  | Yes | 0 | Yes | 14 | 0.004016 | 1251 |
| phchp073v2 | SZA | M | 50 | Caucasian | No | No | No |  | Yes | 0 | Yes | 14 | 0.004167 | 1125 |
| phchp073v3 | SZA | M | 50 | Caucasian | No | No | No |  | Yes | 0 | Yes | 14 | 0.00428 | 1036 |
| phchp074v1 | SZA | F | 46 | African American | No | No | No |  | Yes | 0 | Yes | 0 | 0 | 3390 |
| phchp074v2 | SZA | F | 46 | African American | No | No | No |  | Yes | 0 | Yes | 0 | 0 | 3281 |
| phchp074v3 | SZA | F | 46 | African American | No | No | No |  | Yes | 0 | Yes | 0 | 0 | 3187 |
| phchp075v1 | SZA | M | 57 | Caucasian | No | No | No |  | Yes | 0 | Yes | 7 | 0.002106 | 1304 |
| phchp075v2 | SZA | M | 58 | Caucasian | No | No | No |  | Yes | 0 | Yes | 7 | 0.002167 | 1211 |
| phchp075v3 | SZA | M | 58 | Caucasian | No | No | No |  | Yes | 0 | Yes | 7 | 0.002219 | 1134 |
| phchp076v1 | SZA | F | 41 | African American | No | No | No |  | Yes | 1 | Yes | 1 | 0.000299 | 32 |
| phchp076v2 | SZA | F | 41 | African American | No | No | No |  | Yes | 0 | Yes | 0 | 0 | 3247 |
| phchp076v3 | SZA | F | 41 | African American | No | No | No |  | Yes | 0 | Yes | 0 | 0 | 3167 |
| phchp079v1 | BP | M | 44 | Caucasian | No | No | No |  | Yes | 0 | Yes | 0 | 0 | 2691 |
| phchp079v2 | BP | M | 44 | Caucasian | No | No | No |  | Yes | 0 | Yes | 0 | 0 | 2596 |
| phchp079v3 | BP | M | 45 | Caucasian | No | No | No |  | Yes | 0 | Yes | 0 | 0 | 2502 |
| phchp080v1 | BP | M | 44 | Caucasian | No | No | No |  | Yes | 0 | Yes | 1 | 0.000305 | 2698 |
| phchp081v1 | SZA | M | 53 | African American | No | No | No |  | Yes | 1 | Yes | 3 | 0.000897 | 319 |
| phchp081v3 | SZA | M | 53 | African American | No | No | No |  | Yes | 2 | Yes | 3 | 0.000961 | 97 |
| phchp083v1 | SZ | M | 50 | African American | No | No | No |  | Yes | 0 | Yes | 0 | 0 | 3242 |
| phchp083v2 | SZ | M | 50 | African American | No | No | No |  | Yes | 0 | Yes | 0 | 0 | 3142 |
| phchp083v3 | SZ | M | 51 | African American | No | No | No |  | Yes | 0 | Yes | 0 | 0 | 3049 |
| phchp085v1 | SZA | M | 57 | Caucasian | No | No | No |  | Yes | 0 | Yes | 1 | 0.000319 | 3007 |
| phchp085v2 | SZA | M | 57 | Caucasian | No | No | No |  | Yes | 0 | Yes | 1 | 0.000328 | 2919 |
| phchp085v3 | SZA | M | 57 | Caucasian | No | No | No |  | Yes | 0 | Yes | 1 | 0.000336 | 2842 |
| phchp086v1 | SZ | M | 49 | Caucasian | No | No | No |  | Yes | 0 | Yes | 0 | 0 | 3122 |
| phchp086v2 | SZ | M | 49 | Caucasian | No | No | No |  | Yes | 0 | Yes | 0 | 0 | 3013 |
| phchp086v3 | SZ | M | 49 | Caucasian | No | No | No |  | Yes | 0 | Yes | 0 | 0 | 2905 |
| phchp088v1 | BP | M | 44 | Caucasian | No | No | No |  | Yes | 0 | Yes | 3 | 0.000959 | 1997 |
| phchp088v2 | BP | M | 45 | Caucasian | No | No | No |  | Yes | 0 | Yes | 3 | 0.000985 | 1913 |
| phchp088v3 | BP | M | 45 | Caucasian | No | No | No |  | Yes | 0 | Yes | 3 | 0.001031 | 1778 |
| phchp091v1 | SZA | M | 55 | Caucasian | No | No | No |  | Yes | 0 | Yes | 0 | 0 | 2542 |
| phchp091v2 | SZA | M | 55 | Caucasian | No | No | No |  | Yes | 0 | Yes | 0 | 0 | 2462 |
| phchp091v3 | SZA | M | 55 | Caucasian | No | No | No |  | Yes | 0 | Yes | 0 | 0 | 2364 |
| phchp092v1 | BP | M | 45 | African American | No | No | No |  | Yes | 1 | Yes | 2 | 0.000662 | 288 |
| phchp092v2 | BP | M | 46 | African American | No | No | No |  | Yes | 1 | Yes | 2 | 0.000693 | 155 |
| phchp093v1 | BP | M | 51 | Caucasian | No | No | No |  | Yes | 1 | Yes | 7 | 0.00246 | 180 |
| phchp093v4 | BP | M | 56 | Caucasian | No | No | No | 7 | Yes | 1 | Yes | 3 | 0.003492 | 163 |
| phchp093v5 | BP | M | 57 | Caucasian | No | No | No | 24 | Yes | 0 | Yes | 2 | 0.00316 | 416 |
| phchp093v6 | BP | M | 57 | Caucasian | No | No | No | 56 | Yes | 1 | Yes | 2 | 0.004115 | 269 |
| phchp094v1 | BP | M | 41 | African American | No | No | No |  | Yes | 0 | Yes | 0 | 0 | 1696 |
| phchp095v1 | BP | M | 28 | Caucasian | No | No | No |  | Yes | 0 | Yes | 1 | 0.00035 | 2320 |
| phchp099v2 | SZ | M | 49 | Caucasian | No | No | No |  | Yes | 0 | Yes | 0 | 0 | 2661 |
| phchp127v2 | SZA | F | 58 | Caucasian | No | No | No | 47 | Yes | 0 | Yes | 0 | 0 | 626 |
| phchp138v2 | MOOD | M | 59 | African American | No | No | No | 42 | Yes | 0 | Yes | 0 | 0 | 2198 |
| phchp138v3 | MOOD | M | 59 | African American | No | No | No | 41 | Yes | 0 | Yes | 0 | 0 | 2124 |
| phchp142v4 | BP | M | 57 | Caucasian | No | No | No | 3 | Yes | 0 | Yes | 0 | 0 | 1385 |
| phchp142v5 | BP | M | 57 | Caucasian | No | No | No | 3 | Yes | 0 | Yes | 0 | 0 | 1292 |
| phchp142v6 | BP | M | 58 | Caucasian | No | No | No | 10 | Yes | 0 | Yes | 0 | 0 | 1200 |
| phchp144v1 | SZ | M | 56 | African American | No | No | No | 0 | Yes | 0 | Yes | 0 | 0 | 2202 |
| phchp188v1 | PSYCH | M | 48 | African American | No | No | No |  | Yes | 0 | Yes | 3 | 0.000765 | 958 |
| phchp188v2 | PSYCH | M | 49 | African American | No | No | No |  | Yes | 0 | Yes | 3 | 0.000791 | 825 |
| phchp192v2 | SZA | M | 56 | African American | No | No | No | 29 | Yes | 0 | Yes | 0 | 0 | 2182 |
| phchp192v3 | SZA | M | 56 | African American | No | No | No | 32 | Yes | 0 | Yes | 0 | 0 | 2086 |
| phchp197v2 | SZ | M | 57 | Caucasian | No | No | No | 30 | Yes | 0 | Yes | 0 | 0 | 1876 |
| phchp197v3 | SZ | M | 57 | Caucasian | No | No | No | 65 | Yes | 0 | Yes | 0 | 0 | 1778 |
| phchp197v4 | SZ | M | 58 | Caucasian | No | No | No | 19 | Yes | 0 | Yes | 0 | 0 | 1641 |
| phchp200v2 | MDD | M | 57 | Caucasian | No | No | No | 43 | Yes | 0 | Yes | 0 | 0 | 2001 |
| phchp200v3 | MDD | M | 57 | Caucasian | No | No | No | 25 | Yes | 0 | Yes | 0 | 0 | 1904 |
| phchp203v1 | MOOD | M | 59 | African American | No | No | No | 4 | Yes | 0 | Yes | 0 | 0 | 2128 |
| phchp203v2 | MOOD | M | 59 | African American | No | No | No | 2 | Yes | 0 | Yes | 0 | 0 | 2037 |
| phchp206v1 | MDD | M | 59 | African American | No | No | No | 1 | Yes | 0 | Yes | 0 | 0 | 2192 |
| phchp208v1 | MDD | M | 56 | African American | No | No | No | 4 | Yes | 0 | Yes | 0 | 0 | 2171 |
| phchp208v2 | MDD | M | 56 | African American | No | No | No | 10 | Yes | 0 | Yes | 0 | 0 | 2074 |
| phchp208v3 | MDD | M | 58 | African American | No | No | No | 2 | Yes | 0 | Yes | 0 | 0 | 1529 |
| phchp212v2 | MDD | M | 56 | African American | No | No | No | 24 | Yes | 0 | Yes | 0 | 0 | 1796 |
| phchp242v2 | MDD | M | 57 | African American | No | No | No | 60 | Yes | 0 | Yes | 0 | 0 | 1063 |
| phchp242v3 | MDD | M | 57 | African American | No | No | No | 19 | Yes | 0 | Yes | 0 | 0 | 908 |
| phchp259v2 | MDD | M | 57 | Caucasian | No | No | No | 57 | Yes | 0 | Yes | 0 | 0 | 596 |
| phchp259v3 | MDD | M | 57 | Caucasian | No | No | No | 9 | Yes | 0 | Yes | 0 | 0 | 451 |
| phchp270v1 | BP | M | 36 | Caucasian | No | No | No |  | Yes | 0 | Yes | 0 | 0 | 3497 |
| phchp270v2 | BP | M | 36 | Caucasian | No | No | No |  | Yes | 0 | Yes | 0 | 0 | 3280 |
| phchp297v4 | SZA | M | 57 | African American | No | No | No | 0 | Yes | 0 | Yes | 0 | 0 | 590 |
| phchp298v1 | SZA | M | 56 | Caucasian | No | No | No | 3 | Yes | 0 | Yes | 0 | 0 | 1410 |
| phchp298v2 | SZA | M | 56 | Caucasian | No | No | No | 8 | Yes | 0 | Yes | 0 | 0 | 1305 |
| phchp298v3 | SZA | M | 56 | Caucasian | No | No | No | 5 | Yes | 0 | Yes | 0 | 0 | 1206 |
| phchp298v4 | SZA | M | 58 | Caucasian | No | No | No | 53 | Yes | 0 | Yes | 0 | 0 | 616 |
| phchp318v1 | MDD | F | 57 | Caucasian | No | No | No | 66 | Yes | 0 | Yes | 0 | 0 | 1677 |
| phchp318v2 | MDD | F | 57 | Caucasian | No | No | No | 19 | Yes | 0 | Yes | 0 | 0 | 1053 |
| phchp318v3 | MDD | F | 58 | Caucasian | No | No | No | 15 | Yes | 0 | Yes | 0 | 0 | 858 |
| phchp336v2 | MDD | M | 61 | African American | No | No | No | 0 | Yes | 1 | Yes | 1 | 0.001101 | 98 |
| phchp349v1 | PSYCH | M | 58 | Caucasian | No | No | No | 43 | Yes | 0 | Yes | 0 | 0 | 1016 |
| phchp352v2 | MDD | M | 24 | African American | No | No | No | 53 | Yes | 1 | Yes | 2 | 0.002743 | 123 |
| phchp352v3 | MDD | M | 25 | African American | No | No | No | 51 | Yes | 1 | Yes | 1 | 0.001672 | 246 |
| phchp360v3 | BP | F | 57 | Caucasian | No | No | No | 1 | Yes | 0 | Yes | 0 | 0 | 459 |
| phchp365v1 | MDD | M | 59 | African American | No | No | No | 28 | Yes | 1 | Yes | 1 | 0.001701 | 284 |
| phchp366v1 | SZ | M | 56 | Caucasian | No | No | No | 0 | Yes | 0 | Yes | 0 | 0 | 549 |
| phchp115v2 | BP | M | 67 | Caucasian | No | No | Yes | 80 | No | 0 | Yes | 0 | 0 | 2514 |
| phchp115v3 | BP | M | 68 | Caucasian | No | No | Yes | 73 | No | 0 | Yes | 0 | 0 | 2413 |
| phchp143v2 | BP | F | 63 | African American | No | No | Yes | 77 | No | 0 | Yes | 0 | 0 | 1828 |
| phchp287v3 | SZA | M | 60 | Caucasian | No | No | Yes | 80 | No | 0 | Yes | 0 | 0 | 1291 |
| phchp300v5 | BP | M | 50 | Caucasian | No | No | Yes | 5 | No | 0 | Yes | 0 | 0 | 313 |
| phchp300v6 | BP | M | 50 | Caucasian | No | No | Yes | 13 | No | 0 | Yes | 0 | 0 | 236 |
| phchp312v2 | BP | M | 65 | Caucasian | No | No | Yes | 70 | No | 0 | Yes | 0 | 0 | 1258 |
| phchp315v1 | MDD | M | 62 | Caucasian | No | No | Yes | 77 | No | 0 | Yes | 0 | 0 | 1321 |
| phchp332v1 | SZA | F | 47 | African American | No | No | Yes | 56 | No | 2 | Yes | 2 | 0.006289 | 100 |
| phchp332v2 | SZA | F | 48 | African American | No | No | Yes | 28 | No | 2 | Yes | 2 | 0.00885 | 8 |
| phchp332v3 | SZA | F | 48 | African American | No | No | Yes | 24 | No | 0 | Yes | 0 | 0 | 205 |
| phchp349v3 | PSYCH | M | 59 | Caucasian | No | No | Yes | 71 | No | 0 | Yes | 0 | 0 | 771 |
| phchp350v4 | BP | M | 49 | Caucasian | No | No | Yes | 9 | No | 0 | Yes | 0 | 0 | 300 |
| phchp357v5 | BP | M | 46 | Caucasian | No | No | Yes | 56 | No | 0 | Yes | 0 | 0 | 320 |
| phchp003v5 | SZ | M | 59 | African American | No | No | No | 35 | No | 0 | Yes | 0 | 0 | 561 |
| phchp004v2 | SZA | M | 60 | African American | No | No | No | 48 | No | 0 | Yes | 0 | 0 | 1767 |
| phchp004v3 | SZA | M | 60 | African American | No | No | No | 25 | No | 0 | Yes | 0 | 0 | 1657 |
| phchp004v4 | SZA | M | 63 | African American | No | No | No | 42 | No | 0 | Yes | 0 | 0 | 635 |
| phchp019v5 | SZ | M | 59 | African American | No | No | No | 3 | No | 0 | Yes | 0 | 0 | 634 |
| phchp020v1 | BP | M | 62 | Caucasian | No | No | No |  | No | 0 | Yes | 0 | 0 | 3886 |
| phchp020v2 | BP | M | 62 | Caucasian | No | No | No |  | No | 0 | Yes | 0 | 0 | 3805 |
| phchp020v3 | BP | M | 63 | Caucasian | No | No | No |  | No | 0 | Yes | 0 | 0 | 3668 |
| phchp038v3 | SZA | M | 59 | African American | No | No | No |  | No | 0 | Yes | 1 | 0.000404 | 689 |
| phchp060v1 | SZ | M | 62 | Caucasian | No | No | No |  | No | 0 | Yes | 0 | 0 | 2068 |
| phchp065v1 | SZA | M | 62 | Caucasian | No | No | No |  | No | 0 | Yes | 0 | 0 | 3483 |
| phchp065v2 | SZA | M | 62 | Caucasian | No | No | No |  | No | 0 | Yes | 0 | 0 | 3397 |
| phchp065v3 | SZA | M | 62 | Caucasian | No | No | No |  | No | 0 | Yes | 0 | 0 | 3299 |
| phchp072v1 | SZA | M | 60 | Caucasian | No | No | No |  | No | 0 | Yes | 1 | 0.000292 | 774 |
| phchp072v2 | SZA | M | 60 | Caucasian | No | No | No |  | No | 0 | Yes | 1 | 0.000301 | 662 |
| phchp072v3 | SZA | M | 60 | Caucasian | No | No | No |  | No | 0 | Yes | 1 | 0.000312 | 554 |
| phchp087v1 | SZA | M | 65 | Caucasian | No | No | No |  | No | 0 | Yes | 1 | 0.000318 | 2421 |
| phchp087v2 | SZA | M | 66 | Caucasian | No | No | No |  | No | 0 | Yes | 1 | 0.000331 | 2302 |
| phchp087v3 | SZA | M | 66 | Caucasian | No | No | No |  | No | 0 | Yes | 1 | 0.000341 | 2211 |
| phchp098v1 | SZ | M | 59 | African American | No | No | No | 51 | No | 0 | Yes | 0 | 0 | 2780 |
| phchp103v1 | SZA | M | 61 | Caucasian | No | No | No | 28 | No | 0 | Yes | 1 | 0.001066 | 786 |
| phchp105v1 | SZA | M | 59 | Caucasian | No | No | No | 20 | No | 0 | Yes | 0 | 0 | 1030 |
| phchp115v1 | BP | M | 67 | Caucasian | No | No | No | 5 | No | 0 | Yes | 0 | 0 | 2617 |
| phchp127v3 | SZA | F | 59 | Caucasian | No | No | No | 35 | No | 0 | Yes | 0 | 0 | 535 |
| phchp138v1 | MOOD | M | 59 | African American | No | No | No | 51 | No | 0 | Yes | 0 | 0 | 2299 |
| phchp143v1 | BP | F | 62 | African American | No | No | No | 44 | No | 0 | Yes | 0 | 0 | 1928 |
| phchp143v3 | BP | F | 63 | African American | No | No | No | 30 | No | 0 | Yes | 0 | 0 | 1739 |
| phchp165v1 | SZ | M | 60 | African American | No | No | No | 2 | No | 0 | Yes | 0 | 0 | 2160 |
| phchp165v2 | SZ | M | 60 | African American | No | No | No | 2 | No | 0 | Yes | 0 | 0 | 2069 |
| phchp165v3 | SZ | M | 61 | African American | No | No | No | 5 | No | 0 | Yes | 0 | 0 | 1978 |
| phchp184v1 | BP | M | 64 | Caucasian | No | No | No | 21 | No | 0 | Yes | 0 | 0 | 2352 |
| phchp184v2 | BP | M | 64 | Caucasian | No | No | No | 14 | No | 0 | Yes | 0 | 0 | 2259 |
| phchp184v3 | BP | M | 64 | Caucasian | No | No | No | 9 | No | 0 | Yes | 0 | 0 | 2168 |
| phchp191v1 | SZA | M | 58 | African American | No | No | No | 7 | No | 0 | Yes | 0 | 0 | 226 |
| phchp191v2 | SZA | M | 58 | African American | No | No | No | 12 | No | 0 | Yes | 0 | 0 | 131 |
| phchp191v3 | SZA | M | 59 | African American | No | No | No | 3 | No | 0 | Yes | 0 | 0 | 27 |
| phchp198v1 | MDD | M | 61 | Caucasian | No | No | No | 32 | No | 0 | Yes | 0 | 0 | 2227 |
| phchp198v2 | MDD | M | 61 | Caucasian | No | No | No | 12 | No | 0 | Yes | 0 | 0 | 2138 |
| phchp198v4 | MDD | M | 62 | Caucasian | No | No | No | 5 | No | 0 | Yes | 0 | 0 | 1962 |
| phchp211v1 | SZ | M | 62 | Caucasian | No | No | No | 7 | No | 0 | Yes | 0 | 0 | 2127 |
| phchp211v2 | SZ | M | 62 | Caucasian | No | No | No | 29 | No | 0 | Yes | 0 | 0 | 1929 |
| phchp211v3 | SZ | M | 62 | Caucasian | No | No | No | 56 | No | 0 | Yes | 0 | 0 | 1835 |
| phchp219v1 | BP | M | 61 | Caucasian | No | No | No | 39 | No | 0 | Yes | 0 | 0 | 2052 |
| phchp219v2 | BP | M | 61 | Caucasian | No | No | No | 45 | No | 0 | Yes | 0 | 0 | 1943 |
| phchp219v3 | BP | M | 62 | Caucasian | No | No | No | 0 | No | 0 | Yes | 0 | 0 | 1742 |
| phchp222v2 | SZ | M | 60 | Caucasian | No | No | No | 25 | No | 0 | Yes | 0 | 0 | 1810 |
| phchp222v3 | SZ | M | 61 | Caucasian | No | No | No | 14 | No | 0 | Yes | 0 | 0 | 1721 |
| phchp238v1 | MDD | M | 62 | Caucasian | No | No | No | 65 | No | 0 | Yes | 0 | 0 | 2028 |
| phchp238v2 | MDD | M | 63 | Caucasian | No | No | No | 31 | No | 0 | Yes | 0 | 0 | 1938 |
| phchp238v3 | MDD | M | 63 | Caucasian | No | No | No | 65 | No | 0 | Yes | 0 | 0 | 1818 |
| phchp275v1 | SZ | M | 63 | Caucasian | No | No | No | 12 | No | 0 | Yes | 1 | 0.000679 | 1326 |
| phchp275v2 | SZ | M | 63 | Caucasian | No | No | No | 4 | No | 0 | Yes | 1 | 0.000734 | 1217 |
| phchp275v3 | SZ | M | 63 | Caucasian | No | No | No | 10 | No | 0 | Yes | 1 | 0.000803 | 1100 |
| phchp276v1 | SZ | M | 59 | African American | No | No | No | 0 | No | 0 | Yes | 0 | 0 | 1662 |
| phchp276v2 | SZ | M | 59 | African American | No | No | No | 0 | No | 0 | Yes | 0 | 0 | 1568 |
| phchp276v3 | SZ | M | 59 | African American | No | No | No | 0 | No | 0 | Yes | 0 | 0 | 1483 |
| phchp276v4 | SZ | M | 61 | African American | No | No | No | 3 | No | 0 | Yes | 0 | 0 | 777 |
| phchp287v1 | SZA | M | 59 | Caucasian | No | No | No | 2 | No | 0 | Yes | 0 | 0 | 1648 |
| phchp287v2 | SZA | M | 60 | Caucasian | No | No | No | 0 | No | 0 | Yes | 0 | 0 | 1546 |
| phchp312v1 | BP | M | 64 | Caucasian | No | No | No | 55 | No | 0 | Yes | 0 | 0 | 1398 |
| phchp312v3 | BP | M | 65 | caucasian | No | No | No | 7 | No | 0 | Yes | 0 | 0 | 1163 |
| phchp336v3 | MDD | M | 62 | African American | No | No | No | 25 | No | 0 | Yes | 0 | 0 | 803 |
| phchp097v1 | SZA | F | 25 | Caucasian | No | No | Yes | 49 | No |  | No |  |  |  |
| phchp097v3 | SZA | F | 26 | Caucasian | No | No | Yes | 77 | No |  | No |  |  |  |
| phchp170v1 | MDD | F | 26 | Caucasian | No | No | Yes | 37 | No |  | No |  |  |  |
| phchp170v2 | MDD | F | 26 | Caucasian | No | No | Yes | 58 | No |  | No |  |  |  |
| phchp170v3 | MDD | F | 26 | Caucasian | No | No | Yes | 34 | No |  | No |  |  |  |
| phchp172v1 | BP | F | 24 | Caucasian | No | No | Yes | 10 | No |  | No |  |  |  |
| phchp172v2 | BP | F | 24 | Caucasian | No | No | Yes | 24 | No |  | No |  |  |  |
| phchp172v3 | BP | F | 25 | Caucasian | No | No | Yes | 43 | No |  | No |  |  |  |
| phchp179v1 | BP | M | 36 | Caucasian | No | No | Yes | 29 | No |  | No |  |  |  |
| phchp179v2 | BP | M | 37 | Caucasian | No | No | Yes | 17 | No |  | No |  |  |  |
| phchp179v4 | BP | M | 37 | Caucasian | No | No | Yes | 60 | No |  | No |  |  |  |
| phchp180v1 | BP | F | 47 | Caucasian | No | No | Yes | 66 | No |  | No |  |  |  |
| phchp180v2 | BP | F | 47 | Caucasian | No | No | Yes | 25 | No |  | No |  |  |  |
| phchp180v3 | BP | F | 47 | Caucasian | No | No | Yes | 9 | No |  | No |  |  |  |
| phchp181v1 | BP | F | 28 | Caucasian | No | No | Yes | 92 | No |  | No |  |  |  |
| phchp181v3 | BP | F | 28 | Caucasian | No | No | Yes | 85 | No |  | No |  |  |  |
| phchp181v4 | BP | F | 29 | Caucasian | No | No | Yes | 72 | No |  | No |  |  |  |
| phchp189v1 | SZ | M | 25 | Caucasian | No | No | Yes | 72 | No |  | No |  |  |  |
| phchp189v3 | SZ | M | 25 | Caucasian | No | No | Yes | 18 | No |  | No |  |  |  |
| phchp223v2 | SZA | F | 60 | Caucasian | No | No | Yes | 77 | No |  | No |  |  |  |
| phchp225v1 | PSYCH | M | 58 | African American | No | No | Yes | 67 | No |  | No |  |  |  |
| phchp232v1 | SZA | F | 38 | Caucasian | No | No | Yes | 32 | No |  | No |  |  |  |
| phchp232v2 | SZA | F | 38 | Caucasian | No | No | Yes | 15 | No |  | No |  |  |  |
| phchp232v3 | SZA | F | 38 | Caucasian | No | No | Yes | 6 | No |  | No |  |  |  |
| phchp239v1 | SZA | F | 54 | African American | No | No | Yes | 7 | No |  | No |  |  |  |
| phchp239v2 | SZA | F | 54 | African American | No | No | Yes | 7 | No |  | No |  |  |  |
| phchp239v3 | SZA | F | 54 | African American | No | No | Yes | 54 | No |  | No |  |  |  |
| phchp241v1 | BP | M | 52 | Caucasian | No | No | Yes | 8 | No |  | No |  |  |  |
| phchp294v1 | BP | F | 20 | Caucasian | No | No | Yes | 81 | No |  | No |  |  |  |
| phchp331v1 | BP | M | 53 | Caucasian | No | No | Yes | 12 | No |  | No |  |  |  |
